# Systematic Evaluation of How Indicators of Inequity and Disadvantage Are Measured and Reported in Population Health Evidence Syntheses

**DOI:** 10.3390/ijerph22060851

**Published:** 2025-05-29

**Authors:** Christopher J. Gidlow, Aman S. Mankoo, Kate Jolly, Ameeta Retzer

**Affiliations:** 1 School of Medicine, Keele University, University Road, Newcastle under Lyme ST5 5BG, UK; 2 Research and Innovation Department, Midlands Partnership University NHS Foundation Trust, St Georges Hospital, Corporation Street, Stafford ST16 3AG, UK; 3 Centre for Health and Development (CHAD), University of Staffordshire, Leek Road, Stoke-on-Trent ST4 4DF, UK; a_mankoo@outlook.com; 4 Institute of Applied Health Research, Murray Learning Centre, University of Birmingham, Birmingham B15 2TT, UK; c.b.jolly@bham.ac.uk (K.J.); a.retzer@bham.ac.uk (A.R.); 5 National Institute for Health and Care Research (NIHR) West Midlands Applied Research Collaboration (ARC), Birmingham B15 2TT, UK; 6 Centre for Patient Reported Outcomes Research, Institute of Applied Health Research, Murray Learning Centre, University of Birmingham, Birmingham B15 2TT, UK; 7 NIHR Birmingham Biomedical Research Centre, Birmingham B15 2TT, UK

**Keywords:** population health, health inequality, health inequity, evidence synthesis, methodological review

## Abstract

We present a systematic evaluation of population health reviews from the Cochrane Database (January 2013–February 2023) to evaluate how indicators of inequity or disadvantage are considered and reported in population health evidence syntheses. Descriptive analyses explored a representation of reviews across health-determinant categories (primary and secondary categories), summarised equity-focused reviews, and examined proportions and types of reviews that planned/completed a subgroup analysis using ≥1 indicators from the PROGRESS-Plus framework. Of 363 reviews included, a minority focused on interventions targeting wider determinants of health (n = 83, 22.9% as primary category), with a predominance related to individual lifestyle factors (n = 155, 42.7%) or health care services intervention (n = 97, 26.7%). An explicit equity focus was evident in 21 (5.8%) reviews that used PROGRESS/PROGRESS-Plus, and 28 (7.7%) targeting vulnerable groups. Almost half (n = 165, 45.6%) planned a subgroup analysis by ≥1 PROGRESS-Plus indicator, which was completed in 63 reviews (38.2% of 165). The non-completion of planned subgroup analyses was attributed to insufficient data (too few primary studies, data not reported by subgroups). Among the 165 reviews planning a subgroup analysis, age was the most cited indicator (n = 91, 55.2%), followed by gender/sex (n = 67, 40.6%), place (n = 47, 28.5%) and socio-economic status (n = 37, 22.4%). This study highlighted missed opportunities for learning about the impacts of health equity in population health evidence syntheses due to insufficient data. We recommend routine use of PROGRESS-Plus and greater consistency in socio-economic proxies (occupation, education, income, disadvantage measures) to facilitate meta-analyses and subgroup analyses.

## 1. Introduction

Understanding and addressing the differential distribution of health outcomes and intervention effects across population groups are a public health priority [1], recognised by public health researchers, practitioners and policy makers [2,3].

The consideration of health inequity is central to the concept of population health, notwithstanding the debate regarding definitions [4,5]. Population health is determined by the wider environmental context, and different population groups vary in exposure and vulnerability to health risks, thus creating inequities in health [6]. Population health interventions can, therefore, tackle the wider determinants, or target vulnerable populations (e.g., low-income groups, less educated, homeless) who have a greater risk of poor health given their physical, economic, and social circumstance [1,7]. This aligns with Marmot’s principle of proportionate universalism, of reducing health inequity through providing support proportionate to need, whilst also addressing the wider determinants [8].

Health *inequalities* are the observed differences in outcomes across individuals and groups defined on the basis of socio-economic factors (e.g., income, education), geography, individual characteristics (e.g., age, sex, ethnicity), and other social factors (e.g., homelessness [9]). These also serve as an indirect means of evaluating health *inequity* or *disparity* [10]: when differences in health are considered unfair and avoidable because they result from some kind of injustice [11]. Some health differences are unavoidable, such as those linked to genetic predisposition or age [2,9]. But public health policy to reduce population health differences are contingent on them being avoidable (inequitable). Therefore, consistent with a public health remit of reducing avoidable differences within the population’s health, and with terminology used in measurement frameworks [12,13] and guidance [2,12,14], this study considers the measurement and reporting of equity/inequity.

Evidence syntheses are the primary sources of evidence used to inform public health practice and policy [15]. By combining data from large numbers of subgroups across diverse populations and settings, such syntheses should facilitate explorations of equity [16]. Yet, meta-analyses are often undermined by heterogeneity of indicators to examine equity [17,18]. The last 20 years have seen a concerted effort in this area [19]. A 2003 framework of (in)equity indicators, PROGRESS [20], was later augmented to PROGRESS-Plus: Place of residence, Race/ethnicity, Occupation, Gender/sex, Religion, Education, Socioeconomic status, Social capital, and personal characteristics [21]. Subsequent guidance for systematic reviewers is offered in the 2012 PRISMA-Equity (or PRISMA-E) extension [12,14] and Cochrane handbook [2]. Yet there is scope for varied practice: which population characteristic to use and the potential conceptual overlap between them (e.g., place can be a proxy for various area-level characteristics), which indicators best represent a given characteristic (e.g., educational attainment or years completed) and how to categorise them (e.g., low/high; primary/secondary/tertiary education). The relevance and measurement of characteristics might also vary with geographical, social or cultural context (e.g., low–middle- vs. high-income countries).

Others examining equity reporting in evidence syntheses have confirmed inconsistency in how inequality/inequity is reported in health inequality/inequity-focused reviews. Hollands et al. [22] found many reviews that used or intended to use PROGRESS-Plus (through targeted searches). Others have observed varied use of PROGRESS/PROGRESS/Plus and PRISMA-E, and variation in the extent of subgroup analyses [23] and how checklists could or should be applied [22]. Welch et al. [24] reviewed 158 methodological studies that examined how systematic reviews assessed health equity. Most used a descriptive assessment of equity reporting and analysis (140, 88.6%), with 58 studies assessing whether reviewers conducted subgroup analysis using one or more PROGRESS-Plus characteristics.

The present study builds on these reviews, evaluating population health evidence syntheses, where health equity *should* be a routine consideration. Our aims were to: (i) explore the representation of population health reviews across health-determinant categories; (ii) describe those with an equity focus; (iii) examine the proportions and types of reviews that planned/completed subgroup analyses using ≥1 indicators from the PROGRESS-Plus framework.

## 2. Materials and Methods

### 2.1. Design and Search

This was a systematic evaluation of a cohort of Cochrane reviews of population health research [25]. It took the form of an overview of reviews [26] following a modified PRISMA reporting format (Supplementary File S1). The data source was the complete Cochrane Database of Systematic Reviews from 1 January 2013 to 19 February 2023 (n = 5953). The full protocol is available at Research Registry (reviewregistry1717).

### 2.2. Inclusion and Exclusion Criteria

Inclusion/exclusion criteria were refined during an initial calibration phase. We aimed to capture evidence syntheses relevant to population health, with potential for sub-analysis (planned and/or completed), or that targeted ‘vulnerable’ populations. This was to ensure that reviews not specifying an equity focus (a noted weakness [12]), but that could or should have, were not missed. Potentially eligible types of syntheses included reviews of non-clinical interventions (e.g., population health, behavioural and educational interventions, mass media campaigns); reviews of reviews; rapid reviews of population health interventions; reviews of prognostic and non-clinical prototype public health studies.

Reasons for exclusion are detailed in Supplementary File S2. Briefly, we excluded reviews of: diagnostic accuracy studies; qualitative studies; clinical intervention studies; clinical populations; other specific populations (e.g., women with multiple births, people undertaking cosmetic procedures); only low- and middle-income countries (LMIC) to avoid additional complexity through differences in the wider economic, geographical and health context between high-income countries (HIC) and LMICs [2], and related differences in equity measures [27]; health service design, organisation of care and healthcare professionals’ practice; and individual-focused intervention studies. We also excluded scoping reviews as they did not include quantitative syntheses with potential for subgroup analyses, and methodological reviews focused on the methods or processes of research (e.g., participant recruitment, retention or randomisation, or different statistical methods).

### 2.3. Screening

Following a calibration phase using 180 consecutive titles and abstracts (by CG/AR), finalised criteria were used to screen titles/abstracts of 5953 Cochrane Reviews (by CG), with independent assessment (by AM) of a 10% sample selected using an MS Excel random number formula (target of 90% agreement). Full texts of potentially eligible reviews were screened, with independent verification (by AM, 10% random sample, target of 90% agreement). Disagreements were resolved through discussion.

### 2.4. Outcome Selection

Key outcomes included: the mention of inequity, inequality or social patterning in the introduction/methods; the explicit use of PROGRESS/PROGRESS-Plus checklists; targeting a vulnerable population group; explicit planning and/or completing subgroup analyses using PROGRESS-Plus indicator types; indicators used; how they were categorised.

### 2.5. Data Extraction and Analysis

All data were extracted by CG using an MS Excel data extraction form (Supplementary File S3) with independent verification by AM (10% random sample). All disagreements were resolved through discussion. Reviews were not quality appraised given the lack of relevant appraisal tools and limited guidance available for methodological reviews [28]. The analysis was descriptive: mapping reviews to the appropriate Dahlgren and Whitehead health-determinant categories [29], assigning primary and secondary categories if more than one was applicable; describing if/how equity was examined (Supplementary File S4).

## 3. Results

### 3.1. Results of Screening

**Figure 1:**
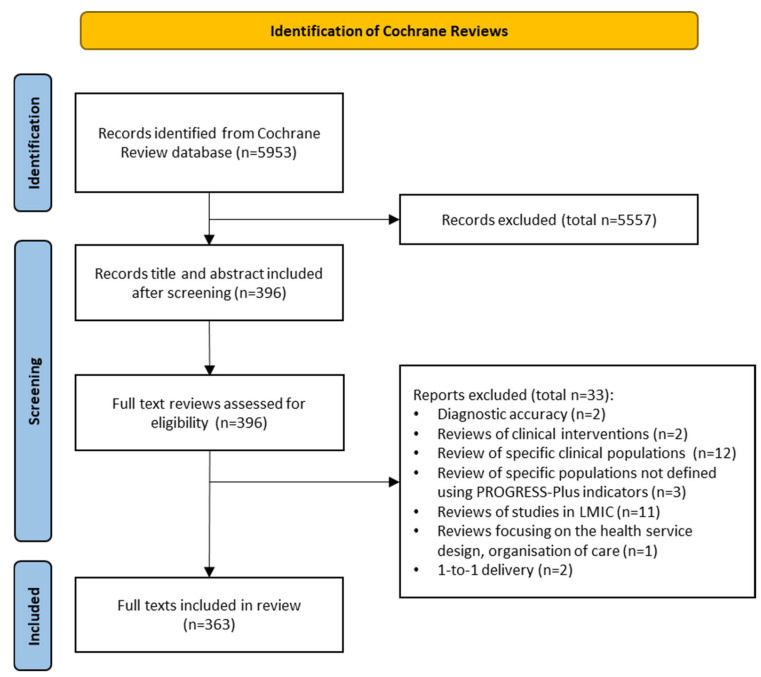
PRISMA flow diagram of study selection (adapted from [39]).

The screening of 5953 review title/abstracts identified 396 full texts that were assessed for eligibility (Figure 1). Thirty-three full texts were excluded, leaving a final sample of 363 reviews (Supplementary File S5). The most common reasons for exclusion were study populations having existing conditions (n = 12) or including only studies from LMIC countries (n = 11) (Supplementary File S6).

### 3.2. Characteristics of Reviews

**Figure 2:**
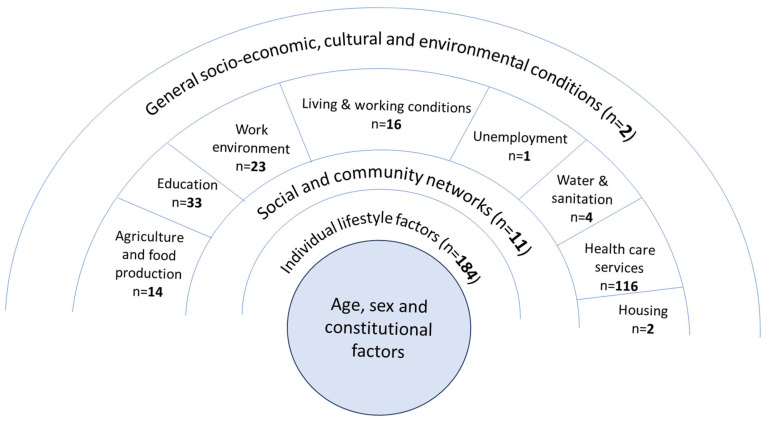
Number of reviews mapped to Dahlgren and Whitehead determinants of health categories (numbers reflect alignment as primary or secondary categories; therefore, the total number (n = 4354) exceeds the number of reviews included (n = 363); see Supplementary File S7.

When mapped to the determinants of health categories, the largest proportions of reviews mapped to individual lifestyle factors (n = 155) or health care services (n = 97) as a primary category, followed by education (n = 30), other (n = 24) and work environment (n = 22). Less than one-quarter of reviews aligned with wider determinants (n = 83 as primary category), reflecting physical living environment (e.g., housing, water sanitation) and broader socio-economic conditions (e.g., general socio-economic conditions, unemployment) or the social environment (social and community networks) (Figure 2; Supplementary File S7).

### 3.3. Measurement and Reporting of Health Inequities or Inequalities

Half the reviews referred to inequalities, inequities or social patterning in the introduction/methods (n = 181, 49.9%). Twenty-eight (7.7%) focused on interventions targeting vulnerable populations, most commonly people with experience of abuse (n = 6), caregivers (n = 4) or people working in environments that expose them to risk (n = 3) (Table 1).

Twenty-one (5.8%) of the 363 reviews used PROGRESS [n = 7, 1.9%] [30,31,32,33,34,35,36] or PROGRESS-Plus [n = 14, 3.9%] [37,38,39,40,41,42,43,44,45,46,47,48,49,50] to extract data and consider equity impacts and disadvantage (Table 2). All were published after the 2012 PRISMA-E extension was introduced [12].

### 3.4. Use of PROGRESS/PROGRESS-Plus

Table 2 summarises the 21 reviews that used PROGRESS or PROGRESS-Plus. All mentioned inequity/inequalities in the introduction/methods, and one focused on a vulnerable group (young people experiencing homelessness [40]). Two reviews did not plan a subgroup analysis by any PROGRESS indicators despite the equity focus [38,46]. A review of psychosocial support for smoking cessation in pregnancy completed most analyses for three PROGRESS-Plus indicators, with a narrative synthesis for others [39].

Eleven reviews reported a narrative synthesis around equity, some with accompanying tables or appendices detailing equity considerations in each study. Of these, six also completed subgroup analyses for some/all of the intended indicators [30,34,36,47,49,50], whereas five planned but did not complete analysis [32,40,42,48,51]. Five reviews included less detailed narratives around equity. Key points were covered, but data were not presented by PROGRESS indicators, often due to a lack of information in primary studies [35,37,41,43,45]. Two reviews, both focused on taxation to improve the health content of food, offered limited consideration of equity impacts [31,33].

Of the 21 reviews that used PROGRESS/PROGRESS-Plus, the predominant determinant of health categories were agriculture and food production (n = 9) and individual lifestyle factors (n = 9) (Table 2).

### 3.5. Subgroup Analysis by PROGRESS-Plus Indicators

Less than half of the 363 reviews (n = 165, 45.7%) planned a subgroup analysis using one or more equity indicators, of which 63 (38.2% of 165, 17.4% of 363) completed analyses. Of the 181 reviews that mentioned inequality, inequity or social patterning in the introduction/methods, 110 (60.8%) planned a subgroup analysis, 71 (39.2%) did not.

**Figure 3:**
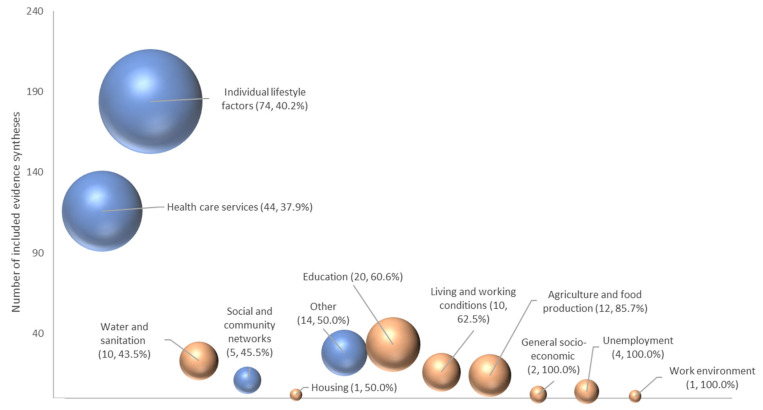
Bubble plot showing the number of reviews in each Dahlgren and Whitehead health-determinant categories (primary or secondary categories; vertical axis) and that planned subgroup analyses (size of bubble and figure in parentheses, percentage as proportion of total in category). Wider health-determinant categories shaded brown; other category types are shaded blue.

Figure 3 illustrates the relative numbers of reviews aligned with each health-determinant category (brown bubbles—wider determinants; blue—other categories) and that planned subgroup analysis. Although relatively few focused on the wider health-determinants, within-category proportions that planned a sub-group analysis were highest for these categories (≥60%, work environment, unemployment, general socio-economic, agriculture and food production, living and working conditions and education). Conversely, for reviews mapped to healthcare services and individual lifestyle factors, the proportions planning a subgroup analysis were lower (≤40%), despite a greater number of reviews.

**Figure 4:**
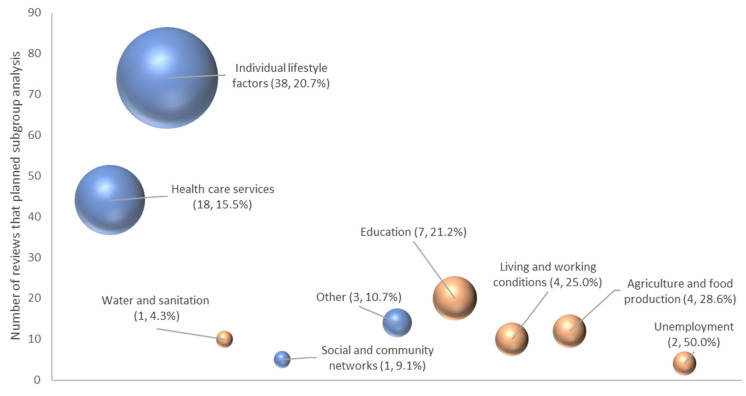
Bubble plot showing the number of reviews that planned (vertical axis) and completed (size of bubble and figure in parentheses, percentage as a proportion of total in category) subgroup analyses by Dahlgren and Whitehead health-determinant categories (primary or secondary categories). Wider health-determinant categories shaded **brown**; other category types are shaded **blue**. Note: housing, general socio-economic and work environment are not represented as no subgroup analyses were completed.

Figure 4 shows the relative numbers of reviews that planned and completed a subgroup analysis, by health-determinant category. Small numbers make the percentages less meaningful, but for categories with 10 or more reviews, the completion rates ranged from 4.3% (water and sanitation) to over 25% (living and working conditions, agriculture and food production). For the most common categories of individual lifestyle factors and healthcare service categories, there were low rates of planned (40.2% and 37.9%) and completed (20.7% and 15.5%) subgroup analyses.

One hundred of the 102 (98.0%) reviews that planned, but did not complete subgroup analyses, cited insufficient data (too few primary studies overall or that reported outcomes by subgroups, or insufficient heterogeneity); two did not specify.

The indicators most intended for/used in subgroup analyses were age, followed by gender/sex, place, socio-economic status (SES) and race/ethnicity (Table 3; Supplementary File S8). There was varied practice. Age categories varied with context and target population, including specific and broad age groups, life stage or school year/stage. Gender/sex groups included a mix of gendered labels (boys/girls, men/women, mothers/fathers) and sex at birth (male/female). Place was mostly country level income (low–middle/high income) or urban/rural location. SES included a range of indicators of disadvantage/social disadvantage/deprivation, income and generic or unspecified proxies. Race/ethnicity groups reflected foci on both ethnicity and race and often compared majority/minority groups, indigenous and non-indigenous groups or groups based on disease risk. In many cases, there was a lack of specificity. Planned and completed subgroup analyses using other equity indicators were uncommon, with few examples of the ‘Plus’ indicators being considered, aside from age (Table 3).

## 4. Discussion

### 4.1. Principal Findings

This systematic evaluation of inequity considerations in population health evidence syntheses confirmed varied practices and limitations, which ultimately limit the evidence base to address health inequity. Although half of the 363 population health reviews (49.9.%) referenced inequities, inequalities or social patterning in their background/methods, few demonstrated a specific equity focus through citing PROGRESS/PROGRESS-Plus (n = 21, 5.8%), in line with the PRISMA-E [2,12], or through targeting vulnerable populations (n = 28, 7.7%).

Compared with other methodological reviews that focused on wider determinants [52] or delimited to health-equity-focused reviews [22,23,24,53], our population health focus was more inclusive. It presents a more critical picture: just 5.8% of the 363 reviews that *should* or *could* have used PROGRESS-Plus for data extraction (as a minimum) did so. This accords with Welch et al. [24] who found that, even among methodological studies of health equity assessments in systematic reviews, just 18 out 158 (11.4%) studies explicitly cited PROGRESS-Plus. A comparison with equivalent proportions reported by Hollands et al. [22] is less useful given their purposive article selection to capture the breadth of approaches (rather than comparing proportions using different approaches). In the present study, it is possible that some review authors, particularly those examining interventions not targeting the wider determinants (77% of included reviews), might not have treated their topic as *population health* and, therefore, not judged equity as a necessary consideration. However, regardless of the authors’ explicit focus, we identified an implicit link to population health and, often, a missed opportunity for equity consideration. Those focused on vulnerable groups explored equity impact through studying populations at greater risk of poor health as a result of unsupportive physical, economic or social circumstances [7]. This might explain why subgroup analysis was planned in only 11 of 28 reviews (completed in just one, by age and gender [54]). There are other examples when certain subgroup analyses would be unnecessary (e.g., by gender if interventions target women; by age if interventions target specific age groups; by occupation if targeting children/older adults/unemployed). These caveats notwithstanding, 39% of reviews that mentioned inequality, inequity or social patterning in the introduction or methods, did not plan any subgroup analysis to examine equity impact. This indicates that equity consideration and reporting are often not routine practice.

We focused on use of subgroup analysis as it allows reviewers to inspect broad patterns of inequity, the limitations aside (e.g., indicators considered in isolation; inability to infer causality [23]). Almost half (45.7%) of the 363 reviews planned subgroup analyses and 17.4% were able to complete some, despite only 5.8% citing PROGRESS/PROGRESS-Plus. Authors of included reviews noted insufficient data to allow analysis through primary studies being too few or not reporting data by subgroups, although the rate of subgroup analyses by one or more PROGRESS-Plus indicator was higher than the 8% (of 262 reviews, across 58 methodological studies) observed by Welch et al. [24].

This deficit in equity reporting among primary studies was reported in a recent assessment of 200 ‘equity-relevant’ studies. Karran et al. [53] found that most studies reported age, sex/gender and education (92%, 78% and 65%, respectively), and approximately half reported other socio-economic proxies (49%), race/ethnicity (45%) and social capital (44%). Yet their analysis identified an overall inadequacy and inconsistency of equity reporting which, they concluded, would likely undermine opportunities to pool data. Indeed, they illustrated that many primary studies fell short of the recommended minimum requirements for sample description by age, gender, ethnicity and a socio-economic measure [55], similar to limitations noted elsewhere [56]. We similarly found that age and gender/sex subgroups were the most used in subgroup analysis, followed by place, race/ethnicity and SES (Table 3). The low rate of planned subgroup analysis by SES (22.4%) and completed analysis in just 4.2% was particularly striking and lower than observed elsewhere [24]. This low rate, in general and relative to other PROGRESS-Plus indicators in the present review, perhaps reflects our broader population health focus, but again shows a deficit in practice.

The relative sparsity of reviews relating to the wider determinants of health speaks to a continued need for further studies and reviews, particularly given that upstream conditions are regarded the key drivers of health and equity [1]. Within this sample of 363 population health evidence syntheses, few aligned with the wider determinants, but were considered ‘population health’ given the potential to affect the social environment (e.g., social support, group-based programmes, family programmes). This under-representation was similar to that observed in a rapid review of population health reviews by Retzer et al. [57]. It reflects a general and longstanding deficit in evidence of how interventions on social determinants impact health inequity [52]. Practical challenges to developing this evidence include the feasibility of intervening in wider environmental conditions. This might require substantial investment (e.g., physical infrastructure), political buy-in (e.g., policy change) or take many years (e.g., housing development), in addition to the complexities of measuring the health effects.

### 4.2. Strengths and Limitations

*Strengths*. The strengths of this systematic evaluation included a population health focus that considered a range of health-determinant categories. The use of the Cochrane Review library as a pragmatic approach offered some advantages regarding quality assurance [58,59] and consistency in the reporting of subgroup analyses.

*Limitations of the evidence included*. This study highlighted the limited reporting and consideration of equity in intervention effects. As few reviews used PROGRESS-Plus or completed a subgroup analysis, there were limited opportunities for comparisons within groups (e.g., by health-determinant category).

*Limitations to the processes*. The single Cochrane data source did not represent the breadth of evidence syntheses. It was not feasible to complete screening and data extraction processes in duplicate for all reviews, and formal quality appraisal was not conducted, a common challenge in methodological reviews [24,28,52]. It was also beyond the scope and resources of this study to consider the full range of approaches to equity assessment [24]. Focusing on use of PROGRESS-Plus, vulnerable populations and subgroup analysis was a feasible approach.

### 4.3. Implications

A more consistent use of tools and processes (PROGRESS-Plus, PRISMA-E) and transparency about which equity indicators were/were not used and why would improve the standards in reporting and subsequent understanding of health inequity. This responsibility falls on both reviewers and primary study authors, for whom PROGRESS-Plus provides a useful framework, in addition to guidance from numerous related articles and reviews published in the last 1–2 years [19,22,23,24,53,55,60,61]. In particular, primary studies in population health should engage with the relevant guidance for collecting and reporting equity data (e.g., [2,60,61,62]), which would give those synthesising evidence options for analysis by population subgroups. Standardisation in approach is not appropriate given the diversity in topics, populations and context. Yet, failure to improve practice to recognise specific groups, as observed with the historical under-representation of women or aggregation of minority ethnic groups in research [63,64], perpetuates invisibility and exclusion from the evidence, leading to evidence-based practice and policy not informed by their interests [65].

There is a need for greater consensus on socio-economic proxies (occupation, education, income, disadvantage measures) to facilitate meta-analyses and subgroup analyses. This is necessary for progress towards governments’ socio-economic equity goals [66,67,68] as reflected in the UN SDGs [to eradicate poverty (#1); achieve good health and well-being for all (#3); reduce inequalities (#10)] [69].

## 5. Conclusions

This systematic evaluation of population health evidence syntheses confirmed deficits in evidence through insufficient and inconsistent practice in reviews and primary studies, which ultimately limit evidence-based public health to address health inequity. Many reviews did not have an explicit equity focus and were often limited to the consideration of age, gender or sex and place. Few were able to complete a subgroup analysis to examine differential health outcomes/intervention effects, often prevented by a small number of primary studies or a lack of detail therein. This highlights missed opportunities for learning about health equity impacts, which could be improved through the routine use of PROGRESS-Plus for data extraction and equity consideration, with transparency regarding which indicators were/not reported and why.

## Figures and Tables

**Table 1:** Number and percentage of reviews targeting different vulnerable population groups.

Vulnerable Population	Number of Reviews	Percentage of Reviews of Vulnerable Populations
Caregivers	4	14.3
Ethnic minorities	1	3.6
Experience of abuse ^a^	6	21.4
Families with attachment issues	1	3.6
Homeless	2	7.1
Lone parents with social welfare support	1	3.6
Mixed ^b^	1	3.6
Poor literacy	1	3.6
Refugees/asylum seekers	2	7.1
Substandard housing	2	7.1
Unemployed adults	1	3.6
Workers exposed to risk	3	10.7
Young offenders	2	7.1
Trachoma endemic area	1	3.6
Total	28	7.7

^a^ Includes review of women, children, partner violence or training of professionals regarding supporting/reporting of violence; ^b^ review authors defined vulnerable populations as groups not covered by health insurance scheme, including children, elderly, women, low-income individuals, rural populations, racial or ethnic minorities, immigrants, informal sector workers and populations with disability or chronic diseases.

**Table 2:** Summary of reviews that cited use of PROGRESS or PROGRESS-Plus checklist.

Study	Title	Health-Determinant Categories	Subgroup Analysis		Checklist	How Progress Was Used (in Addition to Data Extraction)
			Planned	Completed		
Baker 2016 [37]	Interventions for preventing abuse in the elderly	Other	Y	N	PROGRESS-Plus	Planned subgroup analysis by geographical regions, sociodemographic characteristics of the target population. Planned to explore if equity gradient was apparent, if there was increasing gap and decreasing effectiveness by advantaged/disadvantaged populations, but there were insufficient data. ‘Evidence of consideration to equity issues’ reported for each study, but little narrative discussion of equity.
Brown 2019 [34]	Interventions for preventing obesity in children	Individual lifestyle factors	Y	Y	PROGRESS	Completed subgroup analysis by age; within age groups, reported where primary studies had undertaken subgroup analysis by SES, migrant status, ethnicity and rural/urban setting. Reported how studies had targeted disadvantage, with subheading for ‘equity and disadvantage’ and narrative on effects by age, gender, ethnicity, migrant status and urban/rural settings.
Centeno 2019 [38]	Fortification of wheat and maize flour with folic acid for population health outcomes	Agriculture and food production	N	N	PROGRESS-Plus	Recorded if studies included strategies to address diversity or disadvantage. Narrative synthesis described intervention impact by sociodemographic characteristics. Did not report findings by equity indicators.
Chamberlain 2017 [39]	Psychosocial interventions for supporting women to stop smoking in pregnancy	Individual lifestyle factors	Y	Y	PROGRESS-Plus	Used PROGRESS-Plus criteria to categorise interventions provided for vulnerable populations, which might impact vulnerability. Completed subgroup analysis by country income (LMIC, HIC), race/ethnicity (African American, Hispanic) and SES (low/not low), with narrative synthesis for other indicators.
Coren 2016 [40]	Interventions for promoting reintegration and reducing harmful behaviour and lifestyles in street-connected children and young people	Other	Y	N	PROGRESS-Plus	Used PROGRESS-Plus checklist alongside logic model. Planned subgroup analysis by age, gender, location of studies and HIC/LMIC, but there were insufficient data. Narrative examination of equity-related issues in primary studies, focusing on ethnicity, SES, gender, sexual orientation and disability.
Das 2019 [41]	Food fortification with multiple micronutrients: impact on health outcomes in general population	Agriculture and food production	Y	N	PROGRESS-Plus	Planned subgroup analysis by country income (LMIC/HIC) and age, but insufficient information as equity-related variables, and analyses were often missing from the primary studies. Included some descriptive analysis of the PROGRESS-Plus factors reported that highlighted deficient reporting in primary studies.
Garcia-Casal 2018 [42]	Fortification of maize flour with iron for controlling anaemia and iron deficiency in populations	Agriculture and food production	Y	N	PROGRESS-Plus	Recorded whether studies included specific strategies to address diversity or disadvantage. Planned subgroup analysis by gender was not possible. Table of studies reported against each PROGRESS-Plus indicator, with narrative synthesis to describe intervention impact by sociodemographic characteristics.
Hombali 2019 [30]	Fortification of staple foods with vitamin A for vitamin A deficiency	Agriculture and food production	Y	Y	PROGRESS	Recorded whether studies included strategies to address diversity or disadvantage. Planned subgroup analysis by age and gender, completed only for age. Table of studies reported against each PROGRESS indicator (and ‘Plus’) with narrative synthesis to describe intervention impact by sociodemographic characteristics.
Husk 2016 [43]	Participation in environmental enhancement and conservation activities for health and well-being in adults: A review of quantitative and qualitative evidence	Individual lifestyle factors	Y	N	PROGRESS-Plus	Planned subgroup analysis to explore potential impacts by SES, but no studies reported SES. Narrative analysis reported where included studies had undertaken subgroup analysis, but little overall discussion of equity impact.
Lhachimi 2020 [31]	Taxation of the fat content of foods for reducing their consumption and preventing obesity or other adverse health outcomes	Agriculture and food production	Y	N	PROGRESS	Planned subgroup analysis by country income, group income and age (children/adult), but insufficient data. Noted the ‘equity considerations’ for included studies, but there was no narrative synthesis by these factors nor discussion of equity.
MacArthur 2018 [51]	Individual-, family-, and school-level interventions targeting multiple risk behaviours in young people	Individual lifestyle factors	Y	N	PROGRESS-Plus	Planned subgroup analyses for all PROGRESS indicators (but did not specify variables for most). Data within each subgroup for outcomes were insufficient to complete these analyses. Included ‘equity’ section in the Results that provided a narrative description, and noted the limited information on which to draw inferences around equity.
Marx 2017 [35]	Later school start times for supporting the education, health and well-being of high school students	Education	Y	N	PROGRESS	Planned subgroup analyses by gender, age and/or grade, indicators of socioeconomic status and ethnicity, but there were too few studies. Authors included a brief narrative ‘report on equity’ in the Discussion.
McLaren 2016 [36]	Population-level interventions in government jurisdictions for dietary sodium reduction	Agriculture and food production	Y	Y	PROGRESS	Planned subgroup analysis to examine differential impact by multiple axes of social inequality based on PROGRESS indicators, but data only permitted this for gender/sex. Presented narrative synthesis to summarise for the remainder.
Morgan 2020 [45]	Caregiver involvement in interventions for improving children’s dietary intake and physical activity behaviours	Individual lifestyle factors	Y	N	PROGRESS-Plus	Extracted data for all PROGRESS-Plus indicators (including disability, sexual orientation, caregiver civil status). Planned subgroup analysis, but there were insufficient data. Discussion included consideration of implications for health equity and the research needs relevant to the promotion of health equity.
Mosdol 2017 [46]	Targeted mass media interventions promoting healthy behaviours to reduce risk of non-communicable diseases in adult, ethnic minorities	Individual lifestyle factors	N	N	PROGRESS-Plus	No planned subgroup analysis related to PROGRESS-Plus indicators. Narrative consideration of some equity issues (particularly by ethnicity).
Pena-Rosas 2019 [47]	Fortification of rice with vitamins and minerals for addressing micronutrient malnutrition	Agriculture and food production	Y	Y	PROGRESS-Plus	Planned and completed subgroup analysis by malaria endemic/malaria-free location (‘Place’). Included a table of studies reporting against each PROGRESS-Plus indicator and narrative synthesis to describe intervention impact by sociodemographic characteristics.
Pega 2013 [32]	In-work tax credits for families and their impact on health status in adults	General socio-economic	Y	N	PROGRESS	Included data on gender identity (and sexual orientation) and extracted data on inclusion of strategies for supporting disadvantaged populations. Planned subgroup analyses by ethnicity, family type (one-parent family, two-parent family), gender and income were not possible due to a small number of studies. Included an ‘Impact on equity’ section with narrative synthesis. Noted a lack of information available for subgroup analysis.
Petkovic 2021 [48]	Behavioural interventions delivered through interactive social media for health behaviour change, health outcomes and health equity in the adult population	Individual lifestyle factors	Y	N	PROGRESS-Plus	Planned harvest plots to assess the presence of gradients in effects across sex, ethnicity, SES and other PROGRESS-Plus characteristics for each outcome, but there were insufficient data. Used narrative synthesis with an ‘equity’ section in results, summarising data from four studies for which data were available.
Pfindern 2020 [33]	Taxation of unprocessed sugar or sugar-added foods for reducing their consumption and preventing obesity or other adverse health outcomes	Agriculture and food production; individual lifestyle factors	Y	N	PROGRESS	Planned subgroup analyses with data on most PROGRESS categories were not possible due to the inclusion of only one study (which also limited potential narrative synthesis around equity).
Shah 2016 [49]	Fortification of staple foods with zinc for improving zinc status and other health outcomes in the general population	Agriculture and food production	Y	Y	PROGRESS-Plus	Completed subgroup analysis by age. Table of studies reported against each PROGRESS-Plus indicator, and narrative synthesis describe intervention impact by sociodemographic characteristics (mainly limited to age groups).
von Philipsborn 2019 [50]	Environmental interventions to reduce the consumption of sugar-sweetened beverages and their effects on health	Living and working conditions; individual lifestyle factors	Y	Y	PROGRESS-Plus	Completed subgroup analysis by gender/sex. Narrative synthesis of studies reporting subgroup analyses by indicators of social disadvantage (‘SES’) and gender/sex and presented in a separate Appendix.

**Table 3:** Number and proportion of reviews that planned or completed subgroup analysis using equity indicators.

PROGRESS-Plus Indicator Type	Planned		Complete		
	n	% ^a^	n	% ^a^	% ^b^
**P**lace	47	28.5	13	7.9	21.0
**R**ace/ethnicity	31	18.8	8	4.8	12.9
**O**ccupation	9	5.5	1	0.6	1.6
**G**ender/sex	69	41.8	24	14.5	38.7
**R**eligion	3	1.8	0	0.0	0.0
**E**ducation	11	6.7	0	0.0	0.0
**S**ocio-economic status (SES)	37	22.4	8	4.2	11.3
**S**ocial capital	2	1.2	0	0.0	0.0
**Plus** Personal characteristics (total)	91	55.2	30	18.2	48.4
—Variable: Age	91	55.2	30	18.2	48.4
—Variable: Disability	3	1.8	1	0.6	1.6
—Variable: Sexual orientation	1	0.6	0	0.0	0.0
Relationships (total)	2	1.2	0	0.0	0.0
—Variable: Family type	1	0.6	0	0.0	0.0
—Variable: Parents of child with disability	1	0.6	0	0.0	0.0
Time-dependant relationships (total)	3	1.8	0	0.0	0.0
—Variable: Residential history (sheltered/unsheltered housing)	1	0.6	0	0.0	0.0
—Variable: Exposure to injury risk	1	0.6	0	0.0	0.0
—Variable: Victim, perpetrator	1	0.6	0	0.0	0.0
Total count of indicator use	400		115		

^a^ % figures are proportions of 165 reviews that planned subgroup analyses using equity indicators. ^b^ % figures are proportions of 63 reviews that completed subgroup analyses using equity indicators.

## Data Availability

Data supporting reported results can be requested from the authors.

## Supplementary Materials

The following supporting information can be downloaded at: https://www.mdpi.com/article/10.3390/ijerph22060851/s1, Supplementary File S1: PRISMA Checklist; Supplementary File S2: Types of excluded reviews with reasons [70,71,72,73]; Supplementary File S3: Data extraction form fields; Supplementary File S4: Summary of descriptive analysis of included reviews; Supplementary File S5: Characteristics of included reviews (n = 363) [74,75,76,77,78,79,80,81,82,83,84,85,86,87,88,89,90,91,92,93,94,95,96,97,98,99,100,101,102,103,104,105,106,107,108,109,110,111,112,113,114,115,116,117,118,119,120,121,122,123,124,125,126,127,128,129,130,131,132,133,134,135,136,137,138,139,140,141,142,143,144,145,146,147,148,149,150,151,152,153,154,155,156,157,158,159,160,161,162,163,164,165,166,167,168,169,170,171,172,173,174,175,176,177,178,179,180,181,182,183,184,185,186,187,188,189,190,191,192,193,194,195,196,197,198,199,200,201,202,203,204,205,206,207,208,209,210,211,212,213,214,215,216,217,218,219,220,221,222,223,224,225,226,227,228,229,230,231,232,233,234,235,236,237,238,239,240,241,242,243,244,245,246,247,248,249,250,251,252,253,254,255,256,257,258,259,260,261,262,263,264,265,266,267,268,269,270,271,272,273,274,275,276,277,278,279,280,281,282,283,284,285,286,287,288,289,290,291,292,293,294,295,296,297,298,299,300,301,302,303,304,305,306,307,308,309,310,311,312,313,314,315,316,317,318,319,320,321,322,323,324,325,326,327,328,329,330,331,332,333,334,335,336,337,338,339,340,341,342,343,344,345,346,347,348,349,350,351,352,353,354,355,356,357,358,359,360,361,362,363,364,365,366,367,368,369,370,371,372,373,374,375,376,377,378,379,380,381,382,383,384,385,386,387,388,389,390,391,392,393,394,395,396,397,398,399,400,401,402,403,404,405,406,407,408,409,410,411,412,413]; Supplementary File S6: Excluded reviews (n = 33) [414,415,416,417,418,419,420,421,422,423,424,425,426,427,428,429,430,431,432,433,434,435,436,437,438,439,440,441,442,443,444,445,446]; Supplementary File S7: Number of reviews mapped to determinants of health categories (primary/secondary); Supplementary File S8: Breakdown of Place and SES categories planned for subgroup analysis.

## References

[1] Frohlich K.L., Potvin L. (2008). Transcending the known in public health practice: The inequality paradox: The population approach and vulnerable populations. Am. J. Public. Health.

[2] Welch V., Petkovic J., Hartling L., Klassen T., Kristjansson E., Pardo Pardo J., Petticrew M., Stott D., Thomson D., Ueffing E., Higgins J.P.T., Thomas J., Chandler J., Cumpston M., Li T., Page M.J., Welch V.A. (2024). Chapter 16: Equity and Specific Populations. Cochrane Handbook for Systematic Reviews of Interventions Version 6.5.

[3] Porroche-Escudero A., Popay J. (2021). The Health Inequalities Assessment Toolkit: Supporting integration of equity into applied health research. J. Public. Health.

[4] Roux A.V. (2016). On the Distinction-or Lack of Distinction-Between Population Health and Public Health. Am. J. Public. Health.

[5] Moreno-Montoya J. (2023). The practical irrelevance of distinguishing between public health and population health. Rev. De La Univ. Ind. De Santander. Salud.

[6] Rose G.A., Khaw K.-T., Marmot M. (2008). Rose’s Strategy of Preventive Medicine: The Complete Original Text.

[7] Rukmana D., Michalos A.C. (2014). Vulnerable Populations. Encyclopedia of Quality of Life and Well-Being Research.

[8] Marmot M., Allen J., Goldblatt P., Boyce T., Di McNeish M., Grady I.G. (2010). Fair Society, Health Lives: The Marmot Review. Strategic Review of Health Inequalities in England Post-2010.

[9] Arcaya M.C., Arcaya A.L., Subramanian S.V. (2015). Inequalities in health: Definitions, concepts, and theories. Glob. Health Action.

[10] World Health Organization (2013). Handbook on Health Inequality Monitoring: With a Special Focus on Low- and Middle-Income Countries.

[11] Kawachi I., Subramanian S.V., Almeida-Filho N. (2002). A glossary for health inequalities. J. Epidemiol. Community Health.

[12] Welch V., Petticrew M., Tugwell P., Moher D., O’Neill J., Waters E., White H., PRISMA-Equity Bellagio Group (2012). PRISMA-Equity 2012 Extension: Reporting Guidelines for Systematic Reviews with a Focus on Health Equity. PLoS Med..

[13] Hosseinpoor A.R., Nambiar D., Schlotheuber A., Reidpath D., Ross Z. (2016). Health Equity Assessment Toolkit (HEAT): Software for exploring and comparing health inequalities in countries. BMC Med. Res. Methodol..

[14] Welch V., Petticrew M., Petkovic J., Moher D., Waters E., White H., Tugwell P., Atun R., Awasthi S., Barbour V. (2016). Extending the PRISMA statement to equity-focused systematic reviews (PRISMA-E 2012): Explanation and elaboration. J. Clin. Epidemiol..

[15] Watt T., Raymond A., Rachet-Jacquet L. (2022). Quantifying Health Inequalities in England.

[16] Glasziou P., Sanders S. (2002). Investigating causes of heterogeneity in systematic reviews. Stat. Med..

[17] Lavis J.N., Røttingen J.-A., Bosch-Capblanch X., Atun R., El-Jardali F., Gilson L., Lewin S., Oliver S., Ongolo-Zogo P., Haines A. (2012). Guidance for evidence-informed policies about health systems: Linking guidance development to policy development. PLoS Med..

[18] Whitehead M., Petticrew M., Graham H., Macintyre S.J., Bambra C., Egan M. (2004). Evidence for public health policy on inequalities: 2: Assembling the evidence jigsaw. J. Epidemiol. Community Health.

[19] Tugwell P., Petticrew M., Kristjansson E., Welch V., Ueffing E., Waters E., Bonnefoy J., Morgan A., Doohan E., Kelly M.P. (2010). Assessing equity in systematic reviews: Realising the recommendations of the Commission on Social Determinants of Health. BMJ.

[20] Evans T., Brown H. (2003). Road traffic crashes: Operationalizing equity in the context of health sector reform. Inj. Control Saf. Promot..

[21] Kavanaugh J., Oliver S., Lorenc T. (2008). Reflections on developing and using PROGRESS-Plus Equity Update. Equity Update Cochrane Health Equity Methods Group.

[22] Hollands G.J., South E., Shemilt I., Oliver S., Thomas J., Sowden A.J. (2024). Methods used to conceptualize dimensions of health equity impacts of public health interventions in systematic reviews. J. Clin. Epidemiol..

[23] Kunonga T.P., Hanratty B., Bower P., Craig D. (2023). A systematic review finds a lack of consensus in methodological approaches in health inequality/inequity focused reviews. J. Clin. Epidemiol..

[24] Welch V., Dewidar O., Tanjong Ghogomu E., Abdisalam S., Al Ameer A., Barbeau V.I., Brand K., Kebedom K., Benkhalti M., Kristjansson E. (2022). How effects on health equity are assessed in systematic reviews of interventions. Cochrane Database Syst. Rev..

[25] Kyte D., Retzer A., Ahmed K., Keeley T., Armes J., Brown J.M., Calman L., Gavin A., Glaser A.W., Greenfield D.M. (2019). Systematic Evaluation of Patient-Reported Outcome Protocol Content and Reporting in Cancer Trials. JNCI J. Natl. Cancer Inst..

[26] Smith V., Devane D., Begley C.M., Clarke M. (2011). Methodology in conducting a systematic review of systematic reviews of healthcare interventions. BMC Med. Res. Methodol..

[27] Howe L.D., Galobardes B., Matijasevich A., Gordon D., Johnston D., Onwujekwe O., Patel R., Webb E.A., Lawlor D.A., Hargreaves J.R. (2012). Measuring socio-economic position for epidemiological studies in low- and middle-income countries: A methods of measurement in epidemiology paper. Int. J. Epidemiol..

[28] Munn Z., Stern C., Aromataris E., Lockwood C., Jordan Z. (2018). What kind of systematic review should I conduct? A proposed typology and guidance for systematic reviewers in the medical and health sciences. BMC Med. Res. Methodol..

[29] Dahlgren G., Whitehead M. (1991). Policies and Strategies to Promote Social Equity in Health. Background Document to WHO-Strategy Paper for Europe.

[30] Hombali A.S., Solon J.A., Venkatesh B.T., Nair N.S., Peña-Rosas J.P. (2019). Fortification of staple foods with vitamin A for vitamin A deficiency. Cochrane Database Syst. Rev..

[31] Lhachimi S.K., Pega F., Heise T.L., Fenton C., Gartlehner G., Griebler U., Sommer I., Bombana M., Katikireddi S. (2020). Taxation of the fat content of foods for reducing their consumption and preventing obesity or other adverse health outcomes. Cochrane Database Syst. Rev..

[32] Pega F., Carter K., Blakely T., Lucas P.J. (2013). In-work tax credits for families and their impact on health status in adults. Cochrane Database Syst. Rev..

[33] Pfindern M., Heisen T.L., Hiltonn Boon M., Pega F., Fenton C., Griebler U., Gartlehner G., Sommer I., Katikireddi S.V., Lhachimi S.K. (2020). Taxation of unprocessed sugar or sugar-added foods for reducing their consumption and preventing obesity or other adverse health outcomes. Cochrane Database Syst. Rev..

[34] Brown T., Moore T.H.M., Hooper L., Gao Y., Zayegh A., Ijaz S., Elwenspoek M., Foxen S.C., Magee L., O’Malley C. (2019). Interventions for preventing obesity in children. Cochrane Database Syst. Rev..

[35] Marx R., Tanner-Smith E.E., Davison C.M., Ufholz L.A., Freeman J., Shankar R., Newton L., Brown R.S., Parpia A.S., Cozma I. (2017). Later school start times for supporting the education, health, and well-being of high school students. Cochrane Database Syst. Rev..

[36] McLaren L., Sumar N., Barberio A.M., Trieu K., Lorenzetti D.L., Tarasuk V., Webster J., Campbell N.R.C. (2016). Population-level interventions in government jurisdictions for dietary sodium reduction. Cochrane Database Syst. Rev..

[37] Baker P.R.A., Francis D.P., Hairi N.N., Othman S., Choo W.Y. (2016). Interventions for preventing abuse in the elderly. Cochrane Database Syst. Rev..

[38] Centeno Tablante E., Pachón H., Guetterman H.M., Finkelstein J.L. (2019). Fortification of wheat and maize flour with folic acid for population health outcomes. Cochrane Database Syst. Rev..

[39] Chamberlain C., O’Mara-Eves A., Porter J., Coleman T., Perlen S.M., Thomas J., McKenzie J.E. (2017). Psychosocial interventions for supporting women to stop smoking in pregnancy. Cochrane Database Syst. Rev..

[40] Coren E., Hossain R., Pardo Pardo J., Bakker B. (2016). Interventions for promoting reintegration and reducing harmful behaviour and lifestyles in street-connected children and young people. Cochrane Database Syst. Rev..

[41] Das J.K., Salam R.A., Mahmood S.B., Moin A., Kumar R., Mukhtar K., Lassi Z.S., Bhutta Z.A. (2019). Food fortification with multiple micronutrients: Impact on health outcomes in general population. Cochrane Database Syst. Rev..

[42] Garcia-Casal M.N., Peña-Rosas J.P., De-Regil L.M., Gwirtz J.A., Pasricha S.R. (2018). Fortification of maize flour with iron for controlling anaemia and iron deficiency in populations. Cochrane Database Syst. Rev..

[43] Husk K., Lovell R., Cooper C., Stahl-Timmins W., Garside R. (2016). Participation in environmental enhancement and conservation activities for health and well-being in adults: A review of quantitative and qualitative evidence. Cochrane Database Syst. Rev..

[44] McArthur G., Sheehan Y., Badcock N.A., Francis D.A., Wang H.C., Kohnen S., Banales E., Anandakumar T., Marinus E., Castles A. (2018). Phonics training for English-speaking poor readers. Cochrane Database Syst. Rev..

[45] Morgan E.H., Schoonees A., Sriram U., Faure M., Seguin-Fowler R.A. (2020). Caregiver involvement in interventions for improving children’s dietary intake and physical activity behaviors. Cochrane Database Syst. Rev..

[46] Mosdøl A., Lidal I.B., Straumann G.H., Vist G.E. (2017). Targeted mass media interventions promoting healthy behaviours to reduce risk of non-communicable diseases in adult, ethnic minorities. Cochrane Database Syst. Rev..

[47] Peña-Rosas J.P., Mithra P., Unnikrishnan B., Kumar N., De-Regil L.M., Nair N.S., Garcia-Casal M.N., Solon J.A. (2019). Fortification of rice with vitamins and minerals for addressing micronutrient malnutrition. Cochrane Database Syst. Rev..

[48] Petkovic J., Duench S., Trawin J., Dewidar O., Pardo Pardo J., Simeon R., DesMeules M., Gagnon D., Hatcher Roberts J., Hossain A. (2021). Behavioural interventions delivered through interactive social media for health behaviour change, health outcomes, and health equity in the adult population. Cochrane Database Syst. Rev..

[49] Shah D., Sachdev H.S., Gera T., De-Regil L.M., Peña-Rosas J.P. (2016). Fortification of staple foods with zinc for improving zinc status and other health outcomes in the general population. Cochrane Database Syst. Rev..

[50] von Philipsborn P., Stratil J.M., Burns J., Busert L.K., Pfadenhauer L.M., Polus S., Holzapfel C., Hauner H., Rehfuess E. (2019). Environmental interventions to reduce the consumption of sugar-sweetened beverages and their effects on health. Cochrane Database Syst. Rev..

[51] MacArthur G., Caldwell D.M., Redmore J., Watkins S.H., Kipping R., White J., Chittleborough C., Langford R., Er V., Lingam R. (2018). Individual-, family-, and school-level interventions targeting multiple risk behaviours in young people. Cochrane Database Syst. Rev..

[52] Bambra C., Gibson M., Sowden A., Wright K., Whitehead M., Petticrew M. (2010). Tackling the wider social determinants of health and health inequalities: Evidence from systematic reviews. J. Epidemiol. Community Health.

[53] Karran E.L., Cashin A.G., Barker T., Boyd M.A., Chiarotto A., Dewidar O., Mohabir V., Petkovic J., Sharma S., Tejani S. (2023). Using PROGRESS-plus to identify current approaches to the collection and reporting of equity-relevant data: A scoping review. J. Clin. Epidemiol..

[54] O’Hara L., Smith E.R., Barlow J., Livingstone N., Herath N., Wei Y., Spreckelsen T.F., Macdonald G. (2019). Video feedback for parental sensitivity and attachment security in children under five years. Cochrane Database Syst. Rev..

[55] Revenson T.A., Zoccola P.M. (2022). New Instructions to Authors Emphasize Open Science, Transparency, Full Reporting of Sociodemographic Characteristics of the Sample, and Avoidance of Piecemeal Publication. Ann. Behav. Med..

[56] Kayani Z., Willis A., Salisu-Olatunji S.O., Jeffers S., Khunti K., Routen A. (2024). Reporting and representation of underserved groups in intervention studies for patients with multiple long-term conditions: A systematic review. J. R. Soc. Med..

[57] Retzer A., Jolly K., Calvert M., Adab P., Campbell N., Varney J. Methodology Generation for Core Outcome Data Sets in Population Health Research. https://fundingawards.nihr.ac.uk/award/NIHR135211.

[58] Petticrew M., Wilson P., Wright K., Song F. (2002). Quality of Cochrane reviews. Quality of Cochrane reviews is better than that of non-Cochrane reviews. BMJ.

[59] Howick J., Koletsi D., Ioannidis J.P.A., Madigan C., Pandis N., Loef M., Walach H., Sauer S., Kleijnen J., Seehra J. (2022). Most healthcare interventions tested in Cochrane Reviews are not effective according to high quality evidence: A systematic review and meta-analysis. J. Clin. Epidemiol..

[60] Wang X., Dewidar O., Rizvi A., Huang J., Desai P., Doyle R., Ghogomu E., Rader T., Nicholls S.G., Antequera A. (2023). A scoping review establishes need for consensus guidance on reporting health equity in observational studies. J. Clin. Epidemiol..

[61] Rizvi A., Lawson D.O., Young T., Dewidar O., Nicholls S., Akl E.A., Little J., Magwood O., Shamseer L., Ghogomu E. (2022). Guidance relevant to the reporting of health equity in observational research: A scoping review protocol. BMJ Open.

[62] Mbuagbaw L., Aves T., Shea B., Jull J., Welch V., Taljaard M., Yoganathan M., Greer-Smith R., Wells G., Tugwell P. (2017). Considerations and guidance in designing equity-relevant clinical trials. Int. J. Equity Health.

[63] Merriman R., Galizia I., Tanaka S., Sheffel A., Buse K., Hawkes S. (2021). The gender and geography of publishing: A review of sex/gender reporting and author representation in leading general medical and global health journals. BMJ Glob. Health.

[64] Kauh T.J., Read J.G., Scheitler A.J. (2021). The Critical Role of Racial/Ethnic Data Disaggregation for Health Equity. Popul. Res. Policy Rev..

[65] Rochester L., Carroll C. (2022). Implications of research that excludes under-served populations. Nat. Rev. Neurol..

[66] Department for Levelling Up, Housing and Communities (DLUHC) (2022). Levelling up the United Kingdom.

[67] World Health Organization (2019). Strategic Mapping of Institutional Frameworks and Their Approach to Equity.

[68] DeSalvo K.B., Wang Y.C., Harris A., Auerbach J., Koo D., O’Carroll P. (2017). Public Health 3.0: A Call to Action for Public Health to Meet the Challenges of the 21st Century. Prev. Chronic Dis..

[69] United Nations Department of Economic and Social Affairs (2023). The Sustainable Development Goals Report 2023: Special Edition—July 2023.

[70] Clarke V., Braun V. (2013). Successful Qualitative Research: A Practical Guide for Beginners.

[71] World Health Organization (2022). European Regional Obesity Report 2022.

[72] Office for National Statistics Childbearing for Women Born in Different Years, England and Wales: 2020. https://www.ons.gov.uk/peoplepopulationandcommunity/birthsdeathsandmarriages/conceptionandfertilityrates/bulletins/childbearingforwomenbornindifferentyearsenglandandwales/2020.

[73] Retzer A., Adab P., Calvert M., Campbell N., Varney J., Fisher P., Arhin-Tenkorang D., Merriman J., Harris I., Khatsuria F. (2023). Core outcome set development in population health: Potential opportunities and methodological guidance. BMJ Public Health.

[74] Abe S.K., Balogun O.O., Ota E., Takahashi K., Mori R. (2016). Supplementation with multiple micronutrients for breastfeeding women for improving outcomes for the mother and baby. Cochrane Database Syst. Rev..

[75] Adler A.J., Taylor F., Martin N., Gottlieb S., Taylor R.S., Ebrahim S. (2014). Reduced dietary salt for the prevention of cardiovascular disease. Cochrane Database Syst. Rev..

[76] Akl E.A., Kairouz V.F., Sackett K.M., Erdley W.S., Mustafa R.A., Fiander M., Gabriel C., Schünemann H. (2013). Educational games for health professionals. Cochrane Database Syst. Rev..

[77] Al-Khudairy L., Flowers N., Wheelhouse R., Ghannam O., Hartley L., Stranges S., Rees K. (2017). Vitamin C supplementation for the primary prevention of cardiovascular disease. Cochrane Database Syst. Rev..

[78] Al-Khudairy L., Loveman E., Colquitt J.L., Mead E., Johnson R.E., Fraser H., Olajide J., Murphy M., Velho R.M., O’Malley C. (2017). Diet, physical activity and behavioural interventions for the treatment of overweight or obese adolescents aged 12 to 17 years. Cochrane Database Syst. Rev..

[79] Allaouat S., Reddy V.K., Räsänen K., Khan S., Lumens M. (2020). Educational interventions for preventing lead poisoning in workers. Cochrane Database Syst. Rev..

[80] Andras A., Ferket B. (2014). Screening for peripheral arterial disease. Cochrane Database Syst. Rev..

[81] Arikpo D., Edet E.S., Chibuzor M.T., Odey F., Caldwell D.M. (2018). Educational interventions for improving primary caregiver complementary feeding practices for children aged 24 months and under. Cochrane Database Syst. Rev..

[82] Baker R., Camosso-Stefinovic J., Gillies C., Shaw E.J., Cheater F., Flottorp S., Robertson N., Wensing M., Fiander M., Eccles M.P. (2015). Tailored interventions to address determinants of practice. Cochrane Database Syst. Rev..

[83] Bala M.M., Strzeszynski L., Topor-Madry R. (2017). Mass media interventions for smoking cessation in adults. Cochrane Database Syst. Rev..

[84] Balogun O.O., da Silva Lopes K., Ota E., Takemoto Y., Rumbold A., Takegata M., Mori R. (2016). Vitamin supplementation for preventing miscarriage. Cochrane Database Syst. Rev..

[85] Bao Y., Tu X., Wei Q. (2020). Water for preventing urinary stones. Cochrane Database Syst. Rev..

[86] Barlow J., Bergman H., Kornør H., Wei Y., Bennett C. (2016). Group-based parent training programmes for improving emotional and behavioural adjustment in young children. Cochrane Database Syst. Rev..

[87] Bergman H., Henschke N., Hungerford D., Pitan F., Ndwandwe D., Cunliffe N., Soares-Weiser K. (2021). Vaccines for preventing rotavirus diarrhoea: Vaccines in use. Cochrane Database Syst. Rev..

[88] Bergwall S., Johansson A., Sonestedt E., Acosta S. (2022). High versus low-added sugar consumption for the primary prevention of cardiovascular disease. Cochrane Database Syst. Rev..

[89] Bjelakovic G., Gluud L.L., Nikolova D., Whitfield K., Krstic G., Wetterslev J., Gluud C. (2014). Vitamin D supplementation for prevention of cancer in adults. Cochrane Database Syst. Rev..

[90] Bjelakovic G., Gluud L.L., Nikolova D., Whitfield K., Wetterslev J., Simonetti R.G., Bjelakovic M., Gluud C. (2014). Vitamin D supplementation for prevention of mortality in adults. Cochrane Database Syst. Rev..

[91] Buppasiri P., Lumbiganon P., Thinkhamrop J., Ngamjarus C., Laopaiboon M., Medley N. (2015). Calcium supplementation (other than for preventing or treating hypertension) for improving pregnancy and infant outcomes. Cochrane Database Syst. Rev..

[92] Burns J., Movsisyan A., Stratil J.M., Biallas R.L., Coenen M., Emmert-Fees K.M.F., Geffert K., Hoffmann S., Horstick O., Laxy M. (2021). International travel-related control measures to contain the COVID-19 pandemic: A rapid review. Cochrane Database Syst. Rev..

[93] Cahill K., Lancaster T. (2014). Workplace interventions for smoking cessation. Cochrane Database Syst. Rev..

[94] Carberry A.E., Gordon A., Bond D.M., Hyett J., Raynes-Greenow C.H., Jeffery H.E. (2014). Customised versus population-based growth charts as a screening tool for detecting small for gestational age infants in low-risk pregnant women. Cochrane Database Syst. Rev..

[95] Carducci B., Keats E.C., Bhutta Z.A. (2021). Zinc supplementation for improving pregnancy and infant outcome. Cochrane Database Syst. Rev..

[96] Carson-Chahhoud K., Ameer F., Sayehmiri K., Hnin K., van Agteren J.E.M., Sayehmiri F., Brinn M.P., Esterman A.J., Chang A.B., Smith B.J. (2017). Mass media interventions for preventing smoking in young people. Cochrane Database Syst. Rev..

[97] Carson-Chahhoud K.V., Livingstone-Banks J., Sharrad K.J., Kopsaftis Z., Brinn M.P., To-A-Nan R., Bond C.M. (2019). Community pharmacy personnel interventions for smoking cessation. Cochrane Database Syst. Rev..

[98] Cheetham S., Ngo H.T.T., Liira J., Liira H. (2021). Education and training for preventing sharps injuries and splash exposures in healthcare workers. Cochrane Database Syst. Rev..

[99] Clar C., Al-Khudairy L., Loveman E., Kelly S.A.M., Hartley L., Flowers N., Germanò R., Frost G., Rees K. (2017). Low glycaemic index diets for the prevention of cardiovascular disease. Cochrane Database Syst. Rev..

[100] Clarke E.L., Evans J.R., Smeeth L. (2018). Community screening for visual impairment in older people. Cochrane Database Syst. Rev..

[101] Colquitt J.L., Loveman E., O’Malley C., Azevedo L.B., Mead E., Al-Khudairy L., Ells L.J., Metzendorf M.I., Rees K. (2016). Diet, physical activity, and behavioural interventions for the treatment of overweight or obesity in preschool children up to the age of 6 years. Cochrane Database Syst. Rev..

[102] Crepinsek M.A., Taylor E.A., Michener K., Stewart F. (2020). Interventions for preventing mastitis after childbirth. Cochrane Database Syst. Rev..

[103] Coppo A., Galanti M.R., Giordano L., Buscemi D., Bremberg S., Faggiano F. (2014). School policies for preventing smoking among young people. Cochrane Database Syst. Rev..

[104] Davidson S.J., Barrett H.L., Price S.A., Callaway L.K., Dekker Nitert M. (2021). Probiotics for preventing gestational diabetes. Cochrane Database Syst. Rev..

[105] De-Regil L.M., Peña-Rosas J.P., Fernández-Gaxiola A.C., Rayco-Solon P. (2015). Effects and safety of periconceptional oral folate supplementation for preventing birth defects. Cochrane Database Syst. Rev..

[106] Delgado-Noguera M.F., Calvache J.A., Bonfill Cosp X., Kotanidou E.P., Galli-Tsinopoulou A. (2015). Supplementation with long chain polyunsaturated fatty acids (LCPUFA) to breastfeeding mothers for improving child growth and development. Cochrane Database Syst. Rev..

[107] Demicheli V., Barale A., Rivetti A. (2015). Vaccines for women for preventing neonatal tetanus. Cochrane Database Syst. Rev..

[108] Demicheli V., Jefferson T., Di Pietrantonj C., Ferroni E., Thorning S., Thomas R.E., Rivetti A. (2018). Vaccines for preventing influenza in the elderly. Cochrane Database Syst. Rev..

[109] Demicheli V., Jefferson T., Ferroni E., Rivetti A., Di Pietrantonj C. (2018). Vaccines for preventing influenza in healthy adults. Cochrane Database Syst. Rev..

[110] Dyakova M., Shantikumar S., Colquitt J.L., Drew C.M., Sime M., MacIver J., Wright N., Clarke A., Rees K. (2016). Systematic versus opportunistic risk assessment for the primary prevention of cardiovascular disease. Cochrane Database Syst. Rev..

[111] Ebbert J.O., Elrashidi M.Y., Stead L.F. (2015). Interventions for smokeless tobacco use cessation. Cochrane Database Syst. Rev..

[112] Evans J.R., Lawrenson J.G. (2017). Antioxidant vitamin and mineral supplements for preventing age-related macular degeneration. Cochrane Database Syst. Rev..

[113] Faggiano F., Minozzi S., Versino E., Buscemi D. (2014). Universal school-based prevention for illicit drug use. Cochrane Database Syst. Rev..

[114] Fanshawe T.R., Halliwell W., Lindson N., Aveyard P., Livingstone-Banks J., Hartmann-Boyce J. (2017). Tobacco cessation interventions for young people. Cochrane Database Syst. Rev..

[115] Faseru B., Richter K.P., Scheuermann T.S., Park E.W. (2018). Enhancing partner support to improve smoking cessation. Cochrane Database Syst. Rev..

[116] Fellmeth G.L.T., Heffernan C., Nurse J., Habibula S., Sethi D. (2013). Educational and skills-based interventions for preventing relationship and dating violence in adolescents and young adults. Cochrane Database Syst. Rev..

[117] Fernández-Gaxiola A., De-Regil L. (2019). Intermittent iron supplementation for reducing anaemia and its associated impairments in adolescent and adult menstruating women. Cochrane Database Syst. Rev..

[118] Ferri M., Allara E., Bo A., Gasparrini A., Faggiano F. (2013). Media campaigns for the prevention of illicit drug use in young people. Cochrane Database Syst. Rev..

[119] Fiander M., McGowan J., Grad R., Pluye P., Hannes K., Labrecque M., Roberts N.W., Salzwedel D.M., Welch V., Tugwell P. (2015). Interventions to increase the use of electronic health information by healthcare practitioners to improve clinical practice and patient outcomes. Cochrane Database Syst. Rev..

[120] Filippini T., Malavolti M., Borrelli F., Izzo A.A., Fairweather-Tait S.J., Horneber M., Vinceti M. (2020). Green tea (Camellia sinensis) for the prevention of cancer. Cochrane Database Syst. Rev..

[121] Flodgren G., Gonçalves-Bradley D.C., Summerbell C.D. (2017). Interventions to change the behaviour of health professionals and the organisation of care to promote weight reduction in children and adults with overweight or obesity. Cochrane Database Syst. Rev..

[122] Flodgren G., O’Brien M.A., Parmelli E., Grimshaw J.M. (2019). Local opinion leaders: Effects on professional practice and healthcare outcomes. Cochrane Database Syst. Rev..

[123] Forsetlund L., O’Brien M.A., Forsén L., Mwai L., Reinar L.M., Okwen M.P., Horsley T., Rose C.J. (2021). Continuing education meetings and workshops: Effects on professional practice and healthcare outcomes. Cochrane Database Syst. Rev..

[124] Foster C., Richards J., Thorogood M., Hillsdon M. (2013). Remote and web 2.0 interventions for promoting physical activity. Cochrane Database Syst. Rev..

[125] Foxcroft D.R., Moreira M.T., Almeida Santimano N.M.L., Smith L.A. (2015). Social norms information for alcohol misuse in university and college students. Cochrane Database Syst. Rev..

[126] Frazer K., Callinan J.E., McHugh J., van Baarsel S., Clarke A., Doherty K., Kelleher C. (2016). Legislative smoking bans for reducing harms from secondhand smoke exposure, smoking prevalence and tobacco consumption. Cochrane Database Syst. Rev..

[127] Frazer K., McHugh J., Callinan J.E., Kelleher C. (2016). Impact of institutional smoking bans on reducing harms and secondhand smoke exposure. Cochrane Database Syst. Rev..

[128] Gagliardi A.M.Z., Andriolo B.N.G., Torloni M.R., Soares B.G.O., de Oliveira Gomes J., Andriolo R.B., Canteiro Cruz E. (2019). Vaccines for preventing herpes zoster in older adults. Cochrane Database Syst. Rev..

[129] Gartlehner G., Thaler K., Chapman A., Kaminski-Hartenthaler A., Berzaczy D., Van Noord M.G., Helbich T.H. (2013). Mammography in combination with breast ultrasonography versus mammography for breast cancer screening in women at average risk. Cochrane Database Syst. Rev..

[130] Giguère A., Zomahoun H.T., Carmichael P.-H., Uwizeye C.B., Légaré F., Grimshaw J.M., Gagnon M.-P., Auguste D.U., Massougbodji J. (2020). Printed educational materials: Effects on professional practice and healthcare outcomes. Cochrane Database Syst. Rev..

[131] Gonçalves-Bradley D.C.J., Maria A.R., Ricci-Cabello I., Villanueva G., Fønhus M.S., Glenton C., Lewin S., Henschke N., Buckley B.S., Mehl G.L. (2020). Mobile technologies to support healthcare provider to healthcare provider communication and management of care. Cochrane Database Syst. Rev..

[132] Grande A.J., Reid H., Thomas E.E., Nunan D., Foster C. (2016). Exercise prior to influenza vaccination for limiting influenza incidence and its related complications in adults. Cochrane Database Syst. Rev..

[133] Hafdi M., Hoevenaar-Blom M.P., Richard E. (2021). Multi-domain interventions for the prevention of dementia and cognitive decline. Cochrane Database Syst. Rev..

[134] Hameed M., O’Doherty L., Gilchrist G., Tirado-Muñoz J., Taft A., Chondros P., Feder G., Tan M., Hegarty K. (2020). Psychological therapies for women who experience intimate partner violence. Cochrane Database Syst. Rev..

[135] Harding K.B., Peña-Rosas J.P., Webster A.C., Yap C.M.Y., Payne B.A., Ota E., De-Regil L.M. (2017). Iodine supplementation for women during the preconception, pregnancy and postpartum period. Cochrane Database Syst. Rev..

[136] Hartley L., Flowers N., Holmes J., Clarke A., Stranges S., Hooper L., Rees K. (2013). Green and black tea for the primary prevention of cardiovascular disease. Cochrane Database Syst. Rev..

[137] Hartley L., Igbinedion E., Holmes J., Flowers N., Thorogood M., Clarke A., Stranges S., Hooper L., Rees K. (2013). Increased consumption of fruit and vegetables for the primary prevention of cardiovascular diseases. Cochrane Database Syst. Rev..

[138] Hartley L., Dyakova M., Holmes J., Clarke A., Lee M.S., Ernst E., Rees K. (2014). Yoga for the primary prevention of cardiovascular disease. Cochrane Database Syst. Rev..

[139] Hartley L., Clar C., Ghannam O., Flowers N., Stranges S., Rees K. (2015). Vitamin K for the primary prevention of cardiovascular disease. Cochrane Database Syst. Rev..

[140] Hartley L., May M.D., Loveman E., Colquitt J.L., Rees K. (2016). Dietary fibre for the primary prevention of cardiovascular disease. Cochrane Database Syst. Rev..

[141] Hartmann-Boyce J., Chepkin S.C., Ye W., Bullen C., Lancaster T. (2018). Nicotine replacement therapy versus control for smoking cessation. Cochrane Database Syst. Rev..

[142] Hartmann-Boyce J., Livingstone-Banks J., Ordóñez-Mena J.M., Fanshawe T.R., Lindson N., Freeman S.C., Sutton A.J., Theodoulou A., Aveyard P. (2021). Behavioural interventions for smoking cessation: An overview and network meta-analysis. Cochrane Database Syst. Rev..

[143] Hartmann-Boyce J., Theodoulou A., Farley A., Hajek P., Lycett D., Jones L.L., Kudlek L., Heath L., Hajizadeh A., Schenkels M. (2021). Interventions for preventing weight gain after smoking cessation. Cochrane Database Syst. Rev..

[144] Hartmann-Boyce J., Lindson N., Butler A.R., McRobbie H., Bullen C., Begh R., Theodoulou A., Notley C., Rigotti N.A., Turner T. (2022). Electronic cigarettes for smoking cessation. Cochrane Database Syst. Rev..

[145] Hefler M., Liberato S.C., Thomas D.P. (2017). Incentives for preventing smoking in children and adolescents. Cochrane Database Syst. Rev..

[146] Hemilä H., Chalker E. (2013). Vitamin C for preventing and treating the common cold. Cochrane Database Syst. Rev..

[147] Hemilä H., Louhiala P. (2013). Vitamin C for preventing and treating pneumonia. Cochrane Database Syst. Rev..

[148] Hoe V.C.W., Urquhart D.M., Kelsall H.L., Zamri E.N., Sim M.R. (2018). Ergonomic interventions for preventing work-related musculoskeletal disorders of the upper limb and neck among office workers. Cochrane Database Syst. Rev..

[149] Hofmeyr G.J., Lawrie T.A., Atallah Á.N., Torloni M.R. (2018). Calcium supplementation during pregnancy for preventing hypertensive disorders and related problems. Cochrane Database Syst. Rev..

[150] Hofmeyr G.J., Manyame S., Medley N., Williams M.J. (2019). Calcium supplementation commencing before or early in pregnancy, for preventing hypertensive disorders of pregnancy. Cochrane Database Syst. Rev..

[151] Hopewell S., Adedire O., Copsey B.J., Boniface G.J., Sherrington C., Clemson L., Close J.C.T., Lamb S.E. (2018). Multifactorial and multiple component interventions for preventing falls in older people living in the community. Cochrane Database Syst. Rev..

[152] Huey S.L., Acharya N., Silver A., Sheni R., Yu E.A., Peña-Rosas J.P., Mehta S. (2020). Effects of oral vitamin D supplementation on linear growth and other health outcomes among children under five years of age. Cochrane Database Syst. Rev..

[153] Jaafar S.H., Ho J.J., Jahanfar S., Angolkar M. (2016). Effect of restricted pacifier use in breastfeeding term infants for increasing duration of breastfeeding. Cochrane Database Syst. Rev..

[154] Jackson S., Brown J., Norris E., Livingstone-Banks J., Hayes E., Lindson N. (2022). Mindfulness for smoking cessation. Cochrane Database Syst. Rev..

[155] Jacobson Vann J.C., Jacobson R.M., Coyne-Beasley T., Asafu-Adjei J.K., Szilagyi P.G. (2018). Patient reminder and recall interventions to improve immunization rates. Cochrane Database Syst. Rev..

[156] Jahanfar S., Jaafar S.H. (2015). Effects of restricted caffeine intake by mother on fetal, neonatal and pregnancy outcomes. Cochrane Database Syst. Rev..

[157] Jasani B., Simmer K., Patole S.K., Rao S.C. (2017). Long chain polyunsaturated fatty acid supplementation in infants born at term. Cochrane Database Syst. Rev..

[158] Jawad A., Jawad I., Alwan N.A. (2019). Interventions using social networking sites to promote contraception in women of reproductive age. Cochrane Database Syst. Rev..

[159] Jia L., Yuan B., Huang F., Lu Y., Garner P., Meng Q. (2014). Strategies for expanding health insurance coverage in vulnerable populations. Cochrane Database Syst. Rev..

[160] Kalra N., Hooker L., Reisenhofer S., Di Tanna G.L., García-Moreno C. (2021). Training healthcare providers to respond to intimate partner violence against women. Cochrane Database Syst. Rev..

[161] Karsch-Völk M., Barrett B., Kiefer D., Bauer R., Ardjomand-Woelkart K., Linde K. (2014). Echinacea for preventing and treating the common cold. Cochrane Database Syst. Rev..

[162] Kaufman J., Ryan R., Walsh L., Horey D., Leask J., Robinson P., Hill S. (2018). Face-to-face interventions for informing or educating parents about early childhood vaccination. Cochrane Database Syst. Rev..

[163] Keats E.C., Haider B.A., Tam E., Bhutta Z.A. (2019). Multiple-micronutrient supplementation for women during pregnancy. Cochrane Database Syst. Rev..

[164] Kelleher M.M., Phillips R., Brown S.J., Cro S., Cornelius V., Carlsen K.C., Lødrup Skjerven H.O., Rehbinder E.M., Lowe A.J., Dissanayake E. (2022). Skin care interventions in infants for preventing eczema and food allergy. Cochrane Database Syst. Rev..

[165] Kelly S.A.M., Hartley L., Loveman E., Colquitt J.L., Jones H.M., Al-Khudairy L., Clar C., Germanò R., Lunn H.R., Frost G. (2017). Whole grain cereals for the primary or secondary prevention of cardiovascular disease. Cochrane Database Syst. Rev..

[166] Kendrick D., Mulvaney C.A., Ye L., Stevens T., Mytton J.A., Stewart-Brown S. (2013). Parenting interventions for the prevention of unintentional injuries in childhood. Cochrane Database Syst. Rev..

[167] Krishnaratne S., Littlecott H., Sell K., Burns J., Rabe J.E., Stratil J.M., Litwin T., Kreutz C., Coenen M., Geffert K. (2022). Measures implemented in the school setting to contain the COVID-19 pandemic. Cochrane Database Syst. Rev..

[168] Krogsbøll L.T., Jørgensen K.J., Gøtzsche P.C. (2019). General health checks in adults for reducing morbidity and mortality from disease. Cochrane Database Syst. Rev..

[169] Kuehnl A., Seubert C., Rehfuess E., von Elm E., Nowak D., Glaser J. (2019). Human resource management training of supervisors for improving health and well-being of employees. Cochrane Database Syst. Rev..

[170] Kunzler A.M., Helmreich I., Chmitorz A., König J., Binder H., Wessa M., Lieb K. (2020). Psychological interventions to foster resilience in healthcare professionals. Cochrane Database Syst. Rev..

[171] Kuster A.T., Dalsbø T.K., Luong Thanh B.Y., Agarwal A., Durand-Moreau Q.V., Kirkehei I. (2017). Computer-based versus in-person interventions for preventing and reducing stress in workers. Cochrane Database Syst. Rev..

[172] Lak R., Yazdizadeh B., Davari M., Nouhi M., Kelishadi R. (2020). Newborn screening for galactosaemia. Cochrane Database Syst. Rev..

[173] Lassi Z.S., Salam R.A., Haider B.A., Bhutta Z.A. (2013). Folic acid supplementation during pregnancy for maternal health and pregnancy outcomes. Cochrane Database Syst. Rev..

[174] Lawrenson J.G., Evans J.R. (2015). Omega 3 fatty acids for preventing or slowing the progression of age-related macular degeneration. Cochrane Database Syst. Rev..

[175] Law E., Fisher E., Eccleston C., Palermo T.M. (2019). Psychological interventions for parents of children and adolescents with chronic illness. Cochrane Database Syst. Rev..

[176] Lindson N., Klemperer E., Hong B., Ordóñez-Mena J.M., Aveyard P. (2019). Smoking reduction interventions for smoking cessation. Cochrane Database Syst. Rev..

[177] Lindson-Hawley N., Hartmann-Boyce J., Fanshawe T.R., Begh R., Farley A., Lancaster T. (2016). Interventions to reduce harm from continued tobacco use. Cochrane Database Syst. Rev..

[178] Lissiman E., Bhasale A.L., Cohen M. (2014). Garlic for the common cold. Cochrane Database Syst. Rev..

[179] Liu Z., Sun Y.Y., Zhong B.L. (2018). Mindfulness-based stress reduction for family carers of people with dementia. Cochrane Database Syst. Rev..

[180] Livingstone N., Macdonald G., Carr N. (2013). Restorative justice conferencing for reducing recidivism in young offenders (aged 7 to 21). Cochrane Database Syst. Rev..

[181] Livingstone-Banks J., Norris E., Hartmann-Boyce J., West R., Jarvis M., Chubb E., Hajek P. (2019). Relapse prevention interventions for smoking cessation. Cochrane Database Syst. Rev..

[182] Livingstone-Banks J., Ordóñez-Mena J.M., Hartmann-Boyce J. (2019). Print-based self-help interventions for smoking cessation. Cochrane Database Syst. Rev..

[183] Lopez L.M., Grey T.W., Chen M., Denison J., Stuart G. (2016). Behavioral interventions for improving contraceptive use among women living with HIV. Cochrane Database Syst. Rev..

[184] Lopez L.M., Steiner M., Grimes D.A., Hilgenberg D., Schulz K.F. (2013). Strategies for communicating contraceptive effectiveness. Cochrane Database Syst. Rev..

[185] Lopez L.M., Grey T.W., Hiller J.E., Chen M. (2015). Education for contraceptive use by women after childbirth. Cochrane Database Syst. Rev..

[186] Lopez L.M., Bernholc A., Chen M., Tolley E.E. (2016). School-based interventions for improving contraceptive use in adolescents. Cochrane Database Syst. Rev..

[187] Lopez L.M., Grey T.W., Tolley E.E., Chen M. (2016). Brief educational strategies for improving contraception use in young people. Cochrane Database Syst. Rev..

[188] Loveman E., Al-Khudairy L., Johnson R.E., Robertson W., Colquitt J.L., Mead E.L., Ells L.J., Metzendorf M.I., Rees K. (2015). Parent-only interventions for childhood overweight or obesity in children aged 5 to 11 years. Cochrane Database Syst. Rev..

[189] Luong Thanh B.Y., Laopaiboon M., Koh D., Sakunkoo P., Moe H. (2016). Behavioural interventions to promote workers’ use of respiratory protective equipment. Cochrane Database Syst. Rev..

[190] Méndez-Sánchez L., Clark P., Winzenberg T.M., Tugwell P., Correa-Burrows P., Costello R. (2023). Calcium and vitamin D for increasing bone mineral density in premenopausal women. Cochrane Database Syst. Rev..

[191] Makrides M., Crosby D.D., Shepherd E., Crowther C.A. (2014). Magnesium supplementation in pregnancy. Cochrane Database Syst. Rev..

[192] Marinho V.C.C., Worthington H., Walsh T., Chong L.-Y. (2015). Fluoride gels for preventing dental caries in children and adolescents. Cochrane Database Syst. Rev..

[193] Marinho V.C.C., Chong L.-Y., Worthington H.V., Walsh T. (2016). Fluoride mouthrinses for preventing dental caries in children and adolescents. Cochrane Database Syst. Rev..

[194] Martin N., Germanò R., Hartley L., Adler A.J., Rees K. (2015). Nut consumption for the primary prevention of cardiovascular disease. Cochrane Database Syst. Rev..

[195] Maziak W., Jawad M., Jawad S., Ward K.D., Eissenberg T., Asfar T. (2015). Interventions for waterpipe smoking cessation. Cochrane Database Syst. Rev..

[196] McNeill A., Gravely S., Hitchman S.C., Bauld L., Hammond D., Hartmann-Boyce J. (2017). Tobacco packaging design for reducing tobacco use. Cochrane Database Syst. Rev..

[197] Mead E., Brown T., Rees K., Azevedo L.B., Whittaker V., Jones D., Olajide J., Mainardi G.M., Corpeleijn E., O’Malley C. (2017). Diet, physical activity and behavioural interventions for the treatment of overweight or obese children from the age of 6 to 11 years. Cochrane Database Syst. Rev..

[198] Medley N., Vogel J.P., Care A., Alfirevic Z. (2018). Interventions during pregnancy to prevent preterm birth: An overview of Cochrane systematic reviews. Cochrane Database Syst. Rev..

[199] Middleton P., Gomersall J.C., Gould J.F., Shepherd E., Olsen S.F., Makrides M. (2018). Omega-3 fatty acid addition during pregnancy. Cochrane Database Syst. Rev..

[200] Miller B.J., Murray L., Beckmann M.M., Kent T., Macfarlane B. (2013). Dietary supplements for preventing postnatal depression. Cochrane Database Syst. Rev..

[201] Mischke C., Verbeek J.H., Job J., Morata T.C., Alvesalo-Kuusi A., Neuvonen K., Clarke S., Pedlow R.I. (2013). Occupational safety and health enforcement tools for preventing occupational diseases and injuries. Cochrane Database Syst. Rev..

[202] Montesinos-Guevara C., Buitrago-Garcia D., Felix M.L., Guerra C.V., Hidalgo R., Martinez-Zapata M.J., Simancas-Racines D. (2022). Vaccines for the common cold. Cochrane Database Syst. Rev..

[203] Motuhifonua S.K., Lin L., Alsweiler J., Crawford T.J., Crowther C.A. (2023). Antenatal dietary supplementation with myo-inositol for preventing gestational diabetes. Cochrane Database Syst. Rev..

[204] Muktabhant B., Lawrie T.A., Lumbiganon P., Laopaiboon M. (2015). Diet or exercise, or both, for preventing excessive weight gain in pregnancy. Cochrane Database Syst. Rev..

[205] Naude C.E., Visser M.E., Nguyen K.A., Durao S., Schoonees A. (2018). Effects of total fat intake on bodyweight in children. Cochrane Database Syst. Rev..

[206] Ndikom C.M., Fawole B., Ilesanmi R.E. (2014). Extra fluids for breastfeeding mothers for increasing milk production. Cochrane Database Syst. Rev..

[207] Noone C., McSharry J., Smalle M., Burns A., Dwan K., Devane D., Morrissey E.C. (2020). Video calls for reducing social isolation and loneliness in older people: A rapid review. Cochrane Database Syst. Rev..

[208] Notley C., Gentry S., Livingstone-Banks J., Bauld L., Perera R., Hartmann-Boyce J. (2019). Incentives for smoking cessation. Cochrane Database Syst. Rev..

[209] Okolie C., Hawton K., Lloyd K., Price S.F., Dennis M., John A. (2020). Means restriction for the prevention of suicide on roads. Cochrane Database Syst. Rev..

[210] Okolie C., Wood S., Hawton K., Kandalama U., Glendenning A.C., Dennis M., Price S.F., Lloyd K., John A. (2020). Means restriction for the prevention of suicide by jumping. Cochrane Database Syst. Rev..

[211] Ong T.G., Gordon M., Banks S.S.C., Thomas M.R., Akobeng A.K. (2019). Probiotics to prevent infantile colic. Cochrane Database Syst. Rev..

[212] Osborn D.A., Sinn J.K.H. (2013). Prebiotics in infants for prevention of allergy. Cochrane Database Syst. Rev..

[213] Osborn D.A., Sinn J.K.H., Jones L.J. (2018). Infant formulas containing hydrolysed protein for prevention of allergic disease. Cochrane Database Syst. Rev..

[214] Pachito D.V., Eckeli A.L., Desouky A.S., Corbett M.A., Partonen T., Rajaratnam S.M.W., Riera R. (2018). Workplace lighting for improving alertness and mood in daytime workers. Cochrane Database Syst. Rev..

[215] Palacios C., Trak-Fellermeier M.A., Martinez R.X., Lopez-Perez L., Lips P., Salisi J.A., John J.C., Peña-Rosas J.P. (2019). Regimens of vitamin D supplementation for women during pregnancy. Cochrane Database Syst. Rev..

[216] Pantoja T., Grimshaw J.M., Colomer N., Castañon C., Leniz Martelli J. (2019). Manually-generated reminders delivered on paper: Effects on professional practice and patient outcomes. Cochrane Database Syst. Rev..

[217] Peña-Rosas J.P., De-Regil L.M., Garcia-Casal M.N., Dowswell T. (2015). Daily oral iron supplementation during pregnancy. Cochrane Database Syst. Rev..

[218] Peña-Rosas J.P., De-Regil L.M., Gomez Malave H., Flores-Urrutia M.C., Dowswell T. (2015). Intermittent oral iron supplementation during pregnancy. Cochrane Database Syst. Rev..

[219] Peer N., Balakrishna Y., Durao S. (2020). Screening for type 2 diabetes mellitus. Cochrane Database Syst. Rev..

[220] Petrosino A., Turpin-Petrosino C., Hollis-Peel M.E., Lavenberg J.G. (2013). ‘Scared Straight’ and other juvenile awareness programs for preventing juvenile delinquency. Cochrane Database Syst. Rev..

[221] Rees K., Hartley L., Day C., Flowers N., Clarke A., Stranges S. (2013). Selenium supplementation for the primary prevention of cardiovascular disease. Cochrane Database Syst. Rev..

[222] Rees K., Takeda A., Martin N., Ellis L., Wijesekara D., Vepa A., Das A., Hartley L., Stranges S. (2019). Mediterranean-style diet for the primary and secondary prevention of cardiovascular disease. Cochrane Database Syst. Rev..

[223] Rees K., Al-Khudairy L., Takeda A., Stranges S. (2021). Vegan dietary pattern for the primary and secondary prevention of cardiovascular diseases. Cochrane Database Syst. Rev..

[224] Reeves S., Perrier L., Goldman J., Freeth D., Zwarenstein M. (2013). Interprofessional education: Effects on professional practice and healthcare outcomes. Cochrane Database Syst. Rev..

[225] Pelone F., Harrison R., Goldman J., Zwarenstein M. (2017). Interprofessional collaboration to improve professional practice and healthcare outcomes. Cochrane Database Syst. Rev..

[226] Richards J., Hillsdon M., Thorogood M., Foster C. (2013). Face-to-face interventions for promoting physical activity. Cochrane Database Syst. Rev..

[227] Richards J., Thorogood M., Hillsdon M., Foster C. (2013). Face-to-face versus remote and web 2.0 interventions for promoting physical activity. Cochrane Database Syst. Rev..

[228] Rivas C., Ramsay J., Sadowski L., Davidson L.L., Dunne D., Eldridge S., Hegarty K., Taft A., Feder G. (2015). Advocacy interventions to reduce or eliminate violence and promote the physical and psychosocial well-being of women who experience intimate partner abuse. Cochrane Database Syst. Rev..

[229] Rivas C., Vigurs C., Cameron J., Yeo L. (2019). A realist review of which advocacy interventions work for which abused women under what circumstances. Cochrane Database Syst. Rev..

[230] Robertson L., Yeoh S.E., Kolbach D.N. (2013). Non-pharmacological interventions for preventing venous insufficiency in a standing worker population. Cochrane Database Syst. Rev..

[231] Rumbold A., Ota E., Hori H., Miyazaki C., Crowther C.A. (2015). Vitamin E supplementation in pregnancy. Cochrane Database Syst. Rev..

[232] Rumbold A., Ota E., Nagata C., Shahrook S., Crowther C.A. (2015). Vitamin C supplementation in pregnancy. Cochrane Database Syst. Rev..

[233] Rutjes A.W.S., Denton D.A., Di Nisio M., Chong L.Y., Abraham R.P., Al-Assaf A.S., Anderson J.L., Malik M.A., Vernooij R.W.M., Martínez G. (2018). Vitamin and mineral supplementation for maintaining cognitive function in cognitively healthy people in mid and late life. Cochrane Database Syst. Rev..

[234] Saeterdal I., Lewin S., Austvoll-Dahlgren A., Glenton C., Munabi-Babigumira S. (2014). Interventions aimed at communities to inform and/or educate about early childhood vaccination. Cochrane Database Syst. Rev..

[235] Salam R., Zuberi N.F., Bhutta Z.A. (2015). Pyridoxine (vitamin B6) supplementation during pregnancy or labour for maternal and neonatal outcomes. Cochrane Database Syst. Rev..

[236] Sandall J., Soltani H., Gates S., Shennan A., Devane D. (2016). Midwife-led continuity models versus other models of care for childbearing women. Cochrane Database Syst. Rev..

[237] Sangkomkamhang U.S., Lumbiganon P., Laopaiboon M. (2014). Hepatitis B vaccination during pregnancy for preventing infant infection. Cochrane Database Syst. Rev..

[238] Santesso N., Carrasco-Labra A., Brignardello-Petersen R. (2014). Hip protectors for preventing hip fractures in older people. Cochrane Database Syst. Rev..

[239] Sauni R., Verbeek J.H., Uitti J., Jauhiainen M., Kreiss K., Sigsgaard T. (2015). Remediating buildings damaged by dampness and mould for preventing or reducing respiratory tract symptoms, infections and asthma. Cochrane Database Syst. Rev..

[240] Schaafsma F.G., Mahmud N., Reneman M.F., Fassier J.B., Jungbauer F.H.W. (2016). Pre-employment examinations for preventing injury, disease and sick leave in workers. Cochrane Database Syst. Rev..

[241] Schindler T., Sinn J.K.H., Osborn D.A. (2016). Polyunsaturated fatty acid supplementation in infancy for the prevention of allergy. Cochrane Database Syst. Rev..

[242] Schmucker C., Eisele-Metzger A., Meerpohl J.J., Lehane C., Kuellenberg de Gaudry D., Lohner S., Schwingshackl L. (2022). Effects of a gluten-reduced or gluten-free diet for the primary prevention of cardiovascular disease. Cochrane Database Syst. Rev..

[243] Scott A.M., Clark J., Julien B., Islam F., Roos K., Grimwood K., Little P., Del Mar C.B. (2019). Probiotics for preventing acute otitis media in children. Cochrane Database Syst. Rev..

[244] Shrestha N., Kukkonen-Harjula K.T., Verbeek J.H., Ijaz S., Hermans V., Pedisic Z. (2018). Workplace interventions for reducing sitting at work. Cochrane Database Syst. Rev..

[245] Soltan F., Cristofalo D., Marshall D., Purgato M., Taddese H., Vanderbloemen L., Barbui C., Uphoff E. (2022). Community-based interventions for improving mental health in refugee children and adolescents in high-income countries. Cochrane Database Syst. Rev..

[246] Staley H., Shiraz A., Shreeve N., Bryant A., Martin-Hirsch P.P.L., Gajjar K. (2021). Interventions targeted at women to encourage the uptake of cervical screening. Cochrane Database Syst. Rev..

[247] Stead L.F., Carroll A.J., Lancaster T. (2017). Group behaviour therapy programmes for smoking cessation. Cochrane Database Syst. Rev..

[248] Tan M.L., Abrams S.A., Osborn D.A. (2020). Vitamin D supplementation for term breastfed infants to prevent vitamin D deficiency and improve bone health. Cochrane Database Syst. Rev..

[249] Tattan-Birch H., Hartmann-Boyce J., Kock L., Simonavicius E., Brose L., Jackson S., Shahab L., Brown J. (2022). Heated tobacco products for smoking cessation and reducing smoking prevalence. Cochrane Database Syst. Rev..

[250] Taylor G.M.J., Lindson N., Farley A., Leinberger-Jabari A., Sawyer K., te Water Naudé R., Theodoulou A., King N., Burke C., Aveyard P. (2021). Smoking cessation for improving mental health. Cochrane Database Syst. Rev..

[251] Thomas R.E., McLellan J., Perera R. (2013). School-based programmes for preventing smoking. Cochrane Database Syst. Rev..

[252] Thomas R.E., Baker P.R., Thomas B.C., Lorenzetti D.L. (2015). Family-based programmes for preventing smoking by children and adolescents. Cochrane Database Syst. Rev..

[253] Thomas R.E., Lorenzetti D.L. (2018). Interventions to increase influenza vaccination rates of those 60 years and older in the community. Cochrane Database Syst. Rev..

[254] Tieu J., McPhee A.J., Crowther C.A., Middleton P., Shepherd E. (2017). Screening for gestational diabetes mellitus based on different risk profiles and settings for improving maternal and infant health. Cochrane Database Syst. Rev..

[255] Tikka C., Verbeek J.H., Kateman E., Morata T.C., Dreschler W.A., Ferrite S. (2017). Interventions to prevent occupational noise-induced hearing loss. Cochrane Database Syst. Rev..

[256] Uphoff E., Robertson L., Cabieses B., Villalón F.J., Purgato M., Churchill R., Barbui C. (2020). An overview of systematic reviews on mental health promotion, prevention, and treatment of common mental disorders for refugees, asylum seekers, and internally displaced persons. Cochrane Database Syst. Rev..

[257] Ussher M.H., Faulkner G.E.J., Angus K., Hartmann-Boyce J., Taylor A.H. (2019). Exercise interventions for smoking cessation. Cochrane Database Syst. Rev..

[258] van den Brand F.A., Nagelhout G.E., Reda A.A., Winkens B., Evers S., Kotz D., van Schayck O.C.P. (2017). Healthcare financing systems for increasing the use of tobacco dependence treatment. Cochrane Database Syst. Rev..

[259] Vaona A., Banzi R., Kwag K.H., Rigon G., Cereda D., Pecoraro V., Tramacere I., Moja L. (2018). E-learning for health professionals. Cochrane Database Syst. Rev..

[260] Virgara R., Phillips A., Lewis L.K., Baldock K., Wolfenden L., Ferguson T., Richardson M., Okely A., Beets M., Maher C. (2021). Interventions in outside-school hours childcare settings for promoting physical activity amongst schoolchildren aged 4 to 12 years. Cochrane Database Syst. Rev..

[261] Walsh T., Worthington H.V., Glenny A.M., Marinho V.C.C., Jeroncic A. (2019). Fluoride toothpastes of different concentrations for preventing dental caries. Cochrane Database Syst. Rev..

[262] Walsh K., Eggins E., Hine L., Mathews B., Kenny M.C., Howard S., Ayling N., Dallaston E., Pink E., Vagenas D. (2022). Child protection training for professionals to improve reporting of child abuse and neglect. Cochrane Database Syst. Rev..

[263] Whittaker R., McRobbie H., Bullen C., Rodgers A., Gu Y., Dobson R. (2019). Mobile phone text messaging and app-based interventions for smoking cessation. Cochrane Database Syst. Rev..

[264] Wolfenden L., Goldman S., Stacey F.G., Grady A., Kingsland M., Williams C.M., Wiggers J., Milat A., Rissel C., Bauman A. (2018). Strategies to improve the implementation of workplace-based policies or practices targeting tobacco, alcohol, diet, physical activity and obesity. Cochrane Database Syst. Rev..

[265] Wolfenden L., Barnes C., Jones J., Finch M., Wyse R.J., Kingsland M., Tzelepis F., Grady A., Hodder R.K., Booth D. (2020). Strategies to improve the implementation of healthy eating, physical activity and obesity prevention policies, practices or programmes within childcare services. Cochrane Database Syst. Rev..

[266] Yaacob M., Worthington H.V., Deacon S.A., Deery C., Walmsley A.D., Robinson P.G., Glenny A.M. (2014). Powered versus manual toothbrushing for oral health. Cochrane Database Syst. Rev..

[267] Yonemoto N., Nagai S., Mori R. (2021). Schedules for home visits in the early postpartum period. Cochrane Database Syst. Rev..

[268] Young J., Angevaren M., Rusted J., Tabet N. (2015). Aerobic exercise to improve cognitive function in older people without known cognitive impairment. Cochrane Database Syst. Rev..

[269] Abdel-Aleem H., El-Gibaly O.M.H., EL-Gazzar A.F.E.S., Al-Attar G.S.T. (2016). Mobile clinics for women’s and children’s health. Cochrane Database Syst. Rev..

[270] Abdullahi L.H., Kagina B.M., Ndze V.N., Hussey G.D., Wiysonge C.S. (2020). Improving vaccination uptake among adolescents. Cochrane Database Syst. Rev..

[271] Ammenwerth E., Neyer S., Hörbst A., Mueller G., Siebert U., Schnell-Inderst P. (2021). Adult patient access to electronic health records. Cochrane Database Syst. Rev..

[272] Anderson L.M., Adeney K.L., Shinn C., Safranek S., Buckner-Brown J., Krause L.K. (2015). Community coalition-driven interventions to reduce health disparities among racial and ethnic minority populations. Cochrane Database Syst. Rev..

[273] Arora A., Kumbargere Nagraj S., Khattri S., Ismail N.M., Eachempati P. (2022). School dental screening programmes for oral health. Cochrane Database Syst. Rev..

[274] Baker P.R.A., Francis D.P., Soares J., Weightman A.L., Foster C. (2015). Community wide interventions for increasing physical activity. Cochrane Database Syst. Rev..

[275] Barlow J., Smailagic N., Huband N., Roloff V., Bennett C. (2014). Group-based parent training programmes for improving parental psychosocial health. Cochrane Database Syst. Rev..

[276] Bauer A., Rönsch H., Elsner P., Dittmar D., Bennett C., Schuttelaar M.L.A., Lukács J., John S.M., Williams H.C. (2018). Interventions for preventing occupational irritant hand dermatitis. Cochrane Database Syst. Rev..

[277] Borrie F.R.P., Bearn D.R., Innes N.P.T., Iheozor-Ejiofor Z. (2015). Interventions for the cessation of non-nutritive sucking habits in children. Cochrane Database Syst. Rev..

[278] Burns J., Boogaard H., Polus S., Pfadenhauer L.M., Rohwer A.C., van Erp A.M., Turley R., Rehfuess E. (2019). Interventions to reduce ambient particulate matter air pollution and their effect on health. Cochrane Database Syst. Rev..

[279] Byber K., Radtke T., Norbäck D., Hitzke C., Imo D., Schwenkglenks M., Puhan M.A., Dressel H., Mutsch M. (2021). Humidification of indoor air for preventing or reducing dryness symptoms or upper respiratory infections in educational settings and at the workplace. Cochrane Database Syst. Rev..

[280] Catling C.J., Medley N., Foureur M., Ryan C., Leap N., Teate A., Homer C.S.E. (2015). Group versus conventional antenatal care for women. Cochrane Database Syst. Rev..

[281] Chaithongwongwatthana S., Yamasmit W., Limpongsanurak S., Lumbiganon P., Tolosa J.E. (2015). Pneumococcal vaccination during pregnancy for preventing infant infection. Cochrane Database Syst. Rev..

[282] Chastin S., Gardiner P.A., Harvey J.A., Leask C.F., Jerez-Roig J., Rosenberg D., Ashe M.C., Helbostad J.L., Skelton D.A. (2021). Interventions for reducing sedentary behaviour in community-dwelling older adults. Cochrane Database Syst. Rev..

[283] Chen I., Opiyo N., Tavender E., Mortazhejri S., Rader T., Petkovic J., Yogasingam S., Taljaard M., Agarwal S., Laopaiboon M. (2018). Non-clinical interventions for reducing unnecessary caesarean section. Cochrane Database Syst. Rev..

[284] Clar C., Oseni Z., Flowers N., Keshtkar-Jahromi M., Rees K. (2015). Influenza vaccines for preventing cardiovascular disease. Cochrane Database Syst. Rev..

[285] Cooper A.M., O’Malley L.A., Elison S.N., Armstrong R., Burnside G., Adair P., Dugdill L., Pine C. (2013). Primary school-based behavioural interventions for preventing caries. Cochrane Database Syst. Rev..

[286] Crockett R.A., King S.E., Marteau T.M., Prevost A.T., Bignardi G., Roberts N.W., Stubbs B., Hollands G.J., Jebb S.A. (2018). Nutritional labelling for healthier food or non-alcoholic drink purchasing and consumption. Cochrane Database Syst. Rev..

[287] Desapriya E., Harjee R., Brubacher J., Chan H., Hewapathirane D.S., Subzwari S., Pike I. (2014). Vision screening of older drivers for preventing road traffic injuries and fatalities. Cochrane Database Syst. Rev..

[288] Eaton J.C., Rothpletz-Puglia P., Dreker M.R., Iannotti L., Lutter C., Kaganda J., Rayco-Solon P. (2019). Effectiveness of provision of animal-source foods for supporting optimal growth and development in children 6 to 59 months of age. Cochrane Database Syst. Rev..

[289] Edwards A.G.K., Naik G., Ahmed H., Elwyn G.J., Pickles T., Hood K., Playle R. (2013). Personalised risk communication for informed decision making about taking screening tests. Cochrane Database Syst. Rev..

[290] Ejere H.O., Alhassan M.B., Rabiu M. (2015). Face washing promotion for preventing active trachoma. Cochrane Database Syst. Rev..

[291] Els C., Jackson T.D., Milen M.T., Kunyk D., Wyatt G., Sowah D., Hagtvedt R., Deibert D., Straube S. (2020). Random drug and alcohol testing for preventing injury in workers. Cochrane Database Syst. Rev..

[292] Fanshawe T.R., Hartmann-Boyce J., Perera R., Lindson N. (2019). Competitions for smoking cessation. Cochrane Database Syst. Rev..

[293] Freak-Poli R.L.A., Cumpston M., Albarqouni L., Clemes S.A., Peeters A. (2020). Workplace pedometer interventions for increasing physical activity. Cochrane Database Syst. Rev..

[294] Gates N.J., Rutjes A.W.S., Di Nisio M., Karim S., Chong L.Y., March E., Martínez G., Vernooij R.W.M. (2019). Computerised cognitive training for maintaining cognitive function in cognitively healthy people in midlife. Cochrane Database Syst. Rev..

[295] Gates N.J., Rutjes A.W.S., Di Nisio M., Karim S., Chong L.Y., March E., Martínez G., Vernooij R.W.M. (2020). Computerised cognitive training for 12 or more weeks for maintaining cognitive function in cognitively healthy people in late life. Cochrane Database Syst. Rev..

[296] Gibson M., Thomson H., Banas K., Lutje V., McKee M.J., Martin S.P., Fenton C., Bambra C., Bond L. (2018). Welfare-to-work interventions and their effects on the mental and physical health of lone parents and their children. Cochrane Database Syst. Rev..

[297] Gillen P., Sinclair M., Kernohan W.G., Begley C.M., Luyben A.G. (2017). Interventions for prevention of bullying in the workplace. Cochrane Database Syst. Rev..

[298] González-Fraile E., Ballesteros J., Rueda J.-R., Santos-Zorrozúa B., Solà I., McCleery J. (2021). Remotely delivered information, training and support for informal caregivers of people with dementia. Cochrane Database Syst. Rev..

[299] Goyder C., Atherton H., Car M., Heneghan C.J., Car J. (2015). Email for clinical communication between healthcare professionals. Cochrane Database Syst. Rev..

[300] Grande A.J., Keogh J., Silva V., Scott A.M. (2020). Exercise versus no exercise for the occurrence, severity, and duration of acute respiratory infections. Cochrane Database Syst. Rev..

[301] Gulani A., Sachdev H.S. (2014). Zinc supplements for preventing otitis media. Cochrane Database Syst. Rev..

[302] Harrod C.S., Goss C.W., Stallones L., DiGuiseppi C. (2014). Interventions for primary prevention of suicide in university and other post-secondary educational settings. Cochrane Database Syst. Rev..

[303] Hartley L., Flowers N., Lee M.S., Ernst E., Rees K. (2014). Tai chi for primary prevention of cardiovascular disease. Cochrane Database Syst. Rev..

[304] Hartley L., Lee M.S., Kwong J.S.W., Flowers N., Todkill D., Ernst E., Rees K. (2015). Qigong for the primary prevention of cardiovascular disease. Cochrane Database Syst. Rev..

[305] Horvat L., Horey D., Romios P., Kis-Rigo J. (2014). Cultural competence education for health professionals. Cochrane Database Syst. Rev..

[306] Hult M., Lappalainen K., Saaranen T.K., Räsänen K., Vanroelen C., Burdorf A. (2020). Health-improving interventions for obtaining employment in unemployed job seekers. Cochrane Database Syst. Rev..

[307] Iheozor-Ejiofor Z., Worthington H.V., Walsh T., O’Malley L., Clarkson J.E., Macey R., Alam R., Tugwell P., Welch V., Glenny A.M. (2015). Water fluoridation for the prevention of dental caries. Cochrane Database Syst. Rev..

[308] Kendrick D., Kumar A., Carpenter H., Zijlstra G.A.R., Skelton D.A., Cook J.R., Stevens Z., Belcher C.M., Haworth D., Gawler S.J. (2014). Exercise for reducing fear of falling in older people living in the community. Cochrane Database Syst. Rev..

[309] Kew K.M., Carr R., Donovan T., Gordon M. (2017). Asthma education for school staff. Cochrane Database Syst. Rev..

[310] Luger T., Maher C.G., Rieger M.A., Steinhilber B. (2019). Work-break schedules for preventing musculoskeletal symptoms and disorders in healthy workers. Cochrane Database Syst. Rev..

[311] Lumbiganon P., Martis R., Laopaiboon M., Festin M.R., Ho J.J., Hakimi M. (2016). Antenatal breastfeeding education for increasing breastfeeding duration. Cochrane Database Syst. Rev..

[312] Manser R., Lethaby A., Irving L.B., Stone C., Byrnes G., Abramson M.J., Campbell D. (2013). Screening for lung cancer. Cochrane Database Syst. Rev..

[313] Martin A., Booth J.N., Laird Y., Sproule J., Reilly J.J., Saunders D.H. (2018). Physical activity, diet and other behavioural interventions for improving cognition and school achievement in children and adolescents with obesity or overweight. Cochrane Database Syst. Rev..

[314] Mastellos N., Gunn L.H., Felix L.M., Car J., Majeed A. (2014). Transtheoretical model stages of change for dietary and physical exercise modification in weight loss management for overweight and obese adults. Cochrane Database Syst. Rev..

[315] Mulvaney C.A., Smith S., Watson M.C., Parkin J., Coupland C., Miller P., Kendrick D., McClintock H. (2015). Cycling infrastructure for reducing cycling injuries in cyclists. Cochrane Database Syst. Rev..

[316] Munn Z., Tufanaru C., Lockwood C., Stern C., McAneney H., Barker T.H. (2020). Rinse-free hand wash for reducing absenteeism among preschool and school children. Cochrane Database Syst. Rev..

[317] Murtagh E.M., Murphy M.H., Milton K., Roberts N.W., O’Gorman C.S.M., Foster C. (2020). Interventions outside the workplace for reducing sedentary behaviour in adults under 60 years of age. Cochrane Database Syst. Rev..

[318] Naghieh A., Montgomery P., Bonell C.P., Thompson M., Aber J.L. (2015). Organisational interventions for improving wellbeing and reducing work-related stress in teachers. Cochrane Database Syst. Rev..

[319] Norhayati M.N., Ho J.J., Azman M.Y. (2017). Influenza vaccines for preventing acute otitis media in infants and children. Cochrane Database Syst. Rev..

[320] Ojha S., Elfzzani Z., Kwok T.C., Dorling J. (2020). Education of family members to support weaning to solids and nutrition in later infancy in term-born infants. Cochrane Database Syst. Rev..

[321] O’Mahony M., Comber H., Fitzgerald T., Corrigan M.A., Fitzgerald E., Grunfeld E.A., Flynn M.G., Hegarty J. (2017). Interventions for raising breast cancer awareness in women. Cochrane Database Syst. Rev..

[322] Oringanje C., Meremikwu M.M., Eko H., Esu E., Meremikwu A., Ehiri J.E. (2016). Interventions for preventing unintended pregnancies among adolescents. Cochrane Database Syst. Rev..

[323] Orton E., Whitehead J., Mhizha-Murira J., Clarkson M., Watson M.C., Mulvaney C.A., Staniforth J.U.L., Bhuchar M., Kendrick D. (2016). School-based education programmes for the prevention of unintentional injuries in children and young people. Cochrane Database Syst. Rev..

[324] Padhani Z.A., Moazzam Z., Ashraf A., Bilal H., Salam R.A., Das J.K., Bhutta Z.A. (2021). Vitamin C supplementation for prevention and treatment of pneumonia. Cochrane Database Syst. Rev..

[325] Palmer M.J., Henschke N., Bergman H., Villanueva G., Maayan N., Tamrat T., Mehl G.L., Glenton C., Lewin S., Fønhus M.S. (2020). Targeted client communication via mobile devices for improving maternal, neonatal, and child health. Cochrane Database Syst. Rev..

[326] Palmer M.J., Henschke N., Villanueva G., Maayan N., Bergman H., Glenton C., Lewin S., Fønhus M.S., Tamrat T., Mehl G.L. (2020). Targeted client communication via mobile devices for improving sexual and reproductive health. Cochrane Database Syst. Rev..

[327] Parry S.P., Coenen P., Shrestha N., O’Sullivan P.B., Maher C.G., Straker L.M. (2019). Workplace interventions for increasing standing or walking for decreasing musculoskeletal symptoms in sedentary workers. Cochrane Database Syst. Rev..

[328] Pizarro A.B., Persad E., Durao S., Nussbaumer-Streit B., Engela-Volker J.S., McElvenny D., Rhodes S., Stocking K., Fletcher T., Martin C. (2022). Workplace interventions to reduce the risk of SARS-CoV-2 infection outside of healthcare settings. Cochrane Database Syst. Rev..

[329] Poorolajal J., Hooshmand E. (2016). Booster dose vaccination for preventing hepatitis B. Cochrane Database Syst. Rev..

[330] Posadzki P., Mastellos N., Ryan R., Gunn L.H., Felix L.M., Pappas Y., Gagnon M.P., Julious S.A., Xiang L., Oldenburg B. (2016). Automated telephone communication systems for preventive healthcare and management of long-term conditions. Cochrane Database Syst. Rev..

[331] Riggs E., Kilpatrick N., Slack-Smith L., Chadwick B., Yelland J., Muthu M.S., Gomersall J.C. (2019). Interventions with pregnant women, new mothers and other primary caregivers for preventing early childhood caries. Cochrane Database Syst. Rev..

[332] Sánchez G., Nova J., Rodriguez-Hernandez A.E., Medina R.D., Solorzano-Restrepo C., Gonzalez J., Olmos M., Godfrey K., Arevalo-Rodriguez I. (2016). Sun protection for preventing basal cell and squamous cell skin cancers. Cochrane Database Syst. Rev..

[333] Salam R.A., Das J.K., Dojo Soeandy C., Lassi Z.S., Bhutta Z.A. (2015). Impact of Haemophilus influenzae type B (Hib) and viral influenza vaccinations in pregnancy for improving maternal, neonatal and infant health outcomes. Cochrane Database Syst. Rev..

[334] Schmidt B.-M., Durao S., Toews I., Bavuma C.M., Hohlfeld A., Nury E., Meerpohl J.J., Kredo T. (2020). Screening strategies for hypertension. Cochrane Database Syst. Rev..

[335] Shepherd E., Gomersall J.C., Tieu J., Han S., Crowther C.A., Middleton P. (2017). Combined diet and exercise interventions for preventing gestational diabetes mellitus. Cochrane Database Syst. Rev..

[336] Siegfried N., Pienaar D.C., Ataguba J.E., Volmink J., Kredo T., Jere M., Parry C.D.H. (2014). Restricting or banning alcohol advertising to reduce alcohol consumption in adults and adolescents. Cochrane Database Syst. Rev..

[337] Silva J.A., Mininel V.A., Fernandes Agreli H., Peduzzi M., Harrison R., Xyrichis A. (2022). Collective leadership to improve professional practice, healthcare outcomes and staff well-being. Cochrane Database Syst. Rev..

[338] Slanger T.E., Gross J.V., Pinger A., Morfeld P., Bellinger M., Duhme A.L., Reichardt Ortega R.A., Costa G., Driscoll T.R., Foster R.G. (2016). Person-directed, non-pharmacological interventions for sleepiness at work and sleep disturbances caused by shift work. Cochrane Database Syst. Rev..

[339] Smith H.A., Becker G.E. (2016). Early additional food and fluids for healthy breastfed full-term infants. Cochrane Database Syst. Rev..

[340] Steed L., Sohanpal R., Todd A., Madurasinghe V.W., Rivas C., Edwards E.A., Summerbell C.D., Taylor S.J.C., Walton R.T. (2019). Community pharmacy interventions for health promotion: Effects on professional practice and health outcomes. Cochrane Database Syst. Rev..

[341] Strobel N.A., Chamberlain C., Campbell S.K., Shields L., Bainbridge R.G., Adams C., Edmond K.M., Marriott R., McCalman J. (2022). Family-centred interventions for Indigenous early childhood well-being by primary healthcare services. Cochrane Database Syst. Rev..

[342] Takahashi R., Ota E., Hoshi K., Naito T., Toyoshima Y., Yuasa H., Mori R., Nango E. (2017). Fluoride supplementation (with tablets, drops, lozenges or chewing gum) in pregnant women for preventing dental caries in the primary teeth of their children. Cochrane Database Syst. Rev..

[343] Tasnim S., Tang C., Musini V.M., Wright J.M. (2020). Effect of alcohol on blood pressure. Cochrane Database Syst. Rev..

[344] Thomson H., Thomas S., Sellstrom E., Petticrew M. (2013). Housing improvements for health and associated socio-economic outcomes. Cochrane Database Syst. Rev..

[345] Tieu J., Shepherd E., Middleton P., Crowther C.A. (2017). Dietary advice interventions in pregnancy for preventing gestational diabetes mellitus. Cochrane Database Syst. Rev..

[346] Treanor C., Santin O., Prue G., Coleman H., Cardwell C.R., O’Halloran P., Donnelly M. (2019). Psychosocial interventions for informal caregivers of people living with cancer. Cochrane Database Syst. Rev..

[347] Valentine J.C., Leach S.M., Fowler A.P., Stojda D.K., Macdonald G. (2019). Families and Schools Together (FAST) for improving outcomes for children and their families. Cochrane Database Syst. Rev..

[348] van der Molen H.F., Basnet P., Hoonakker P.L.T., Lehtola M.M., Lappalainen J., Frings-Dresen M.H.W., Haslam R., Verbeek J.H. (2018). Interventions to prevent injuries in construction workers. Cochrane Database Syst. Rev..

[349] van Urk F.C., Brown T.W., Waller R., Mayo-Wilson E. (2014). Centre-based day care for children younger than five years of age in high-income countries. Cochrane Database Syst. Rev..

[350] Vaona A., Pappas Y., Grewal R.S., Ajaz M., Majeed A., Car J. (2017). Training interventions for improving telephone consultation skills in clinicians. Cochrane Database Syst. Rev..

[351] Vijayaraghavan M., Elser H., Frazer K., Lindson N., Apollonio D. (2020). Interventions to reduce tobacco use in people experiencing homelessness. Cochrane Database Syst. Rev..

[352] Walsh K., Zwi K., Woolfenden S., Shlonsky A. (2015). School-based education programmes for the prevention of child sexual abuse. Cochrane Database Syst. Rev..

[353] Ward A., Lewis S.R., Weiss H. (2020). Mobility management to prevent, reduce, or delay driving a car in teenagers. Cochrane Database Syst. Rev..

[354] Winokur M., Holtan A., Batchelder K.E. (2014). Kinship care for the safety, permanency, and well-being of children removed from the home for maltreatment. Cochrane Database Syst. Rev..

[355] Worthington H.V., MacDonald L., Poklepovic Pericic T., Sambunjak D., Johnson T.M., Imai P., Clarkson J.E. (2019). Home use of interdental cleaning devices, in addition to toothbrushing, for preventing and controlling periodontal diseases and dental caries. Cochrane Database Syst. Rev..

[356] Yakoob M.Y., Salam R.A., Khan F.R., Bhutta Z.A. (2016). Vitamin D supplementation for preventing infections in children under five years of age. Cochrane Database Syst. Rev..

[357] Yang S., Wu S., Zhou J., Chen X.Y. (2015). Screening for nasopharyngeal cancer. Cochrane Database Syst. Rev..

[358] Yeung C., Chong L.Y., Glenny A.M. (2015). Fluoridated milk for preventing dental caries. Cochrane Database Syst. Rev..

[359] Abdelhamid A.S., Martin N., Bridges C., Brainard J.S., Wang X., Brown T.J., Hanson S., Jimoh O.F., Ajabnoor S.M., Deane K.H.O. (2018). Polyunsaturated fatty acids for the primary and secondary prevention of cardiovascular disease. Cochrane Database Syst. Rev..

[360] Allaf M., Elghazaly H., Mohamed O.G., Fareen M.F., Zaman S., Salmasi A.-M., Tsilidis K., Dehghan A. (2021). Intermittent fasting for the prevention of cardiovascular disease. Cochrane Database Syst. Rev..

[361] Arbyn M., Xu L., Simoens C., Martin-Hirsch P.P.L. (2018). Prophylactic vaccination against human papillomaviruses to prevent cervical cancer and its precursors. Cochrane Database Syst. Rev..

[362] Arditi C., Rège-Walther M., Durieux P., Burnand B. (2017). Computer-generated reminders delivered on paper to healthcare professionals: Effects on professional practice and healthcare outcomes. Cochrane Database Syst. Rev..

[363] Avenell A., Mak J.C.S., O’Connell D.L. (2014). Vitamin D and vitamin D analogues for preventing fractures in post-menopausal women and older men. Cochrane Database Syst. Rev..

[364] Balogun O.O., O’Sullivan E.J., McFadden A., Ota E., Gavine A., Garner C.D., Renfrew M.J., MacGillivray S. (2016). Interventions for promoting the initiation of breastfeeding. Cochrane Database Syst. Rev..

[365] Bauza V., Ye W., Liao J., Majorin F., Clasen T. (2023). Interventions to improve sanitation for preventing diarrhoea. Cochrane Database Syst. Rev..

[366] Behbod B., Sharma M., Baxi R., Roseby R., Webster P. (2018). Family and carer smoking control programmes for reducing children’s exposure to environmental tobacco smoke. Cochrane Database Syst. Rev..

[367] Brand A., Visser M.E., Schoonees A., Naude C.E. (2022). Replacing salt with low-sodium salt substitutes (LSSS) for cardiovascular health in adults, children and pregnant women. Cochrane Database Syst. Rev..

[368] Clasen T.F., Alexander K.T., Sinclair D., Boisson S., Peletz R., Chang H.H., Majorin F., Cairncross S. (2015). Interventions to improve water quality for preventing diarrhoea. Cochrane Database Syst. Rev..

[369] Cormick G., Ciapponi A., Cafferata M.L., Cormick M.S., Belizán J.M. (2022). Calcium supplementation for prevention of primary hypertension. Cochrane Database Syst. Rev..

[370] da Silva Lopes K., Yamaji N., Rahman M.O., Suto M., Takemoto Y., Garcia-Casal M.N., Ota E. (2021). Nutrition-specific interventions for preventing and controlling anaemia throughout the life cycle: An overview of systematic reviews. Cochrane Database Syst. Rev..

[371] Di Pietrantonj C., Rivetti A., Marchione P., Debalini M.G., Demicheli V. (2021). Vaccines for measles, mumps, rubella, and varicella in children. Cochrane Database Syst. Rev..

[372] Ejemot-Nwadiaro R., Ehiri J.E., Arikpo D., Meremikwu M.M., Critchley J.A. (2021). Hand-washing promotion for preventing diarrhoea. Cochrane Database Syst. Rev..

[373] Gavine A., Shinwell S.C., Buchanan P., Farre A., Wade A., Lynn F., Marshall J., Cumming S.E., Dare S., McFadden A. (2022). Support for healthy breastfeeding mothers with healthy term babies. Cochrane Database Syst. Rev..

[374] Gilligan C., Wolfenden L., Foxcroft D.R., Williams A.J., Kingsland M., Hodder R.K., Stockings E., McFadyen T.R., Tindall J., Sherker S. (2019). Family-based prevention programmes for alcohol use in young people. Cochrane Database Syst. Rev..

[375] Graudal N.A., Hubeck-Graudal T., Jurgens G. (2020). Effects of low sodium diet versus high sodium diet on blood pressure, renin, aldosterone, catecholamines, cholesterol, and triglyceride. Cochrane Database Syst. Rev..

[376] Griffith R.J., Alsweiler J., Moore A.E., Brown S., Middleton P., Shepherd E., Crowther C.A. (2020). Interventions to prevent women from developing gestational diabetes mellitus: An overview of Cochrane Reviews. Cochrane Database Syst. Rev..

[377] He F.J., Li J., MacGregor G.A. (2013). Effect of longer-term modest salt reduction on blood pressure. Cochrane Database Syst. Rev..

[378] Hodder R.K., O’Brien K.M., Tzelepis F., Wyse R.J., Wolfenden L. (2020). Interventions for increasing fruit and vegetable consumption in children aged five years and under. Cochrane Database Syst. Rev..

[379] Hollands G.J., Shemilt I., Marteau T.M., Jebb S.A., Lewis H.B., Wei Y., Higgins J.P.T., Ogilvie D. (2015). Portion, package or tableware size for changing selection and consumption of food, alcohol and tobacco. Cochrane Database Syst. Rev..

[380] Hollands G.J., Carter P., Anwer S., King S.E., Jebb S.A., Ogilvie D., Shemilt I., Higgins J.P.T., Marteau T.M. (2019). Altering the availability or proximity of food, alcohol, and tobacco products to change their selection and consumption. Cochrane Database Syst. Rev..

[381] Hooper L., Abdelhamid A., Bunn D., Brown T., Summerbell C.D., Skeaff C.M. (2015). Effects of total fat intake on body weight. Cochrane Database Syst. Rev..

[382] Hooper L., Al-Khudairy L., Abdelhamid A.S., Rees K., Brainard J.S., Brown T.J., Ajabnoor S.M., O’Brien A.T., Winstanley L.E., Donaldson D.H. (2018). Omega-6 fats for the primary and secondary prevention of cardiovascular disease. Cochrane Database Syst. Rev..

[383] Hooper L., Martin N., Jimoh O.F., Kirk C., Foster E., Abdelhamid A.S. (2020). Reduction in saturated fat intake for cardiovascular disease. Cochrane Database Syst. Rev..

[384] Hooper L., Abdelhamid A.S., Jimoh O.F., Bunn D., Skeaff C. (2020). Effects of total fat intake on body fatness in adults. Cochrane Database Syst. Rev..

[385] Imdad A., Mayo-Wilson E., Haykal M.R., Regan A., Sidhu J., Smith A., Bhutta Z.A. (2022). Vitamin A supplementation for preventing morbidity and mortality in children from six months to five years of age. Cochrane Database Syst. Rev..

[386] Jefferson T., Rivetti A., Di Pietrantonj C., Demicheli V. (2018). Vaccines for preventing influenza in healthy children. Cochrane Database Syst. Rev..

[387] Jefferson T., Dooley L., Ferroni E., Al-Ansary L.A., van Driel M.L., Bawazeer G.A., Jones M.A., Hoffmann T.C., Clark J., Beller E.M. (2023). Physical interventions to interrupt or reduce the spread of respiratory viruses. Cochrane Database Syst. Rev..

[388] Kristjansson E., Francis D.K., Liberato S., Benkhalti Jandu M., Welch V., Batal M., Greenhalgh T., Rader T., Noonan E., Shea B. (2015). Food supplementation for improving the physical and psychosocial health of socio-economically disadvantaged children aged three months to five years. Cochrane Database Syst. Rev..

[389] Lassi Z.S., Kurji J., Oliveira C.S.D., Moin A., Bhutta Z.A. (2020). Zinc supplementation for the promotion of growth and prevention of infections in infants less than six months of age. Cochrane Database Syst. Rev..

[390] Lee L.L., Mulvaney C.A., Wong Y.K., Chan E.S.Y., Watson M.C., Lin H.H. (2021). Walking for hypertension. Cochrane Database Syst. Rev..

[391] Low M., Speedy J., Styles C.E., De-Regil L.M., Pasricha S.R. (2016). Daily iron supplementation for improving anaemia, iron status and health in menstruating women. Cochrane Database Syst. Rev..

[392] Low N., Redmond S., Uusküla A., van Bergen J., Ward H., Andersen B., Götz H. (2016). Screening for genital chlamydia infection. Cochrane Database Syst. Rev..

[393] Majorin F., Torondel B., Ka Seen Chan G., Clasen T. (2019). Interventions to improve disposal of child faeces for preventing diarrhoea and soil-transmitted helminth infection. Cochrane Database Syst. Rev..

[394] Mason-Jones A.J., Sinclair D., Mathews C., Kagee A., Hillman A., Lombard C. (2016). School-based interventions for preventing HIV, sexually transmitted infections, and pregnancy in adolescents. Cochrane Database Syst. Rev..

[395] Mayo-Wilson E., Junior J.A., Imdad A., Dean S., Chan X.H.S., Chan E.S., Jaswal A., Bhutta Z.A. (2014). Zinc supplementation for preventing mortality, morbidity, and growth failure in children aged 6 months to 12 years of age. Cochrane Database Syst. Rev..

[396] Mbuagbaw L., Medley N., Darzi A.J., Richardson M., Habiba Garga K., Ongolo-Zogo P. (2015). Health system and community level interventions for improving antenatal care coverage and health outcomes. Cochrane Database Syst. Rev..

[397] Moberley S., Holden J., Tatham D.P., Andrews R.M. (2013). Vaccines for preventing pneumococcal infection in adults. Cochrane Database Syst. Rev..

[398] Moran P.S., Teljeur C., Ryan M., Smith S.M. (2016). Systematic screening for the detection of atrial fibrillation. Cochrane Database Syst. Rev..

[399] Naude C.E., Brand A., Schoonees A., Nguyen K.A., Chaplin M., Volmink J. (2022). Low-carbohydrate versus balanced-carbohydrate diets for reducing weight and cardiovascular risk. Cochrane Database Syst. Rev..

[400] Neil-Sztramko S.E., Caldwell H., Dobbins M. (2021). School-based physical activity programs for promoting physical activity and fitness in children and adolescents aged 6 to 18. Cochrane Database Syst. Rev..

[401] Ota E., Hori H., Mori R., Tobe-Gai R., Farrar D. (2015). Antenatal dietary education and supplementation to increase energy and protein intake. Cochrane Database Syst. Rev..

[402] Palacios C., Kostiuk L.K., Peña-Rosas J.P. (2019). Vitamin D supplementation for women during pregnancy. Cochrane Database Syst. Rev..

[403] Qureshi N., Da Silva M.L.R., Abdul-Hamid H., Weng S.F., Kai J., Leonardi-Bee J. (2021). Strategies for screening for familial hypercholesterolaemia in primary care and other community settings. Cochrane Database Syst. Rev..

[404] Rees K., Dyakova M., Wilson N., Ward K., Thorogood M., Brunner E. (2013). Dietary advice for reducing cardiovascular risk. Cochrane Database Syst. Rev..

[405] Ried K., Fakler P., Stocks N.P. (2017). Effect of cocoa on blood pressure. Cochrane Database Syst. Rev..

[406] Ruotsalainen J.H., Verbeek J.H., Mariné A., Serra C. (2015). Preventing occupational stress in healthcare workers. Cochrane Database Syst. Rev..

[407] Santos J.A.R., Christoforou A., Trieu K., McKenzie B.L., Downs S., Billot L., Webster J., Li M. (2019). Iodine fortification of foods and condiments, other than salt, for preventing iodine deficiency disorders. Cochrane Database Syst. Rev..

[408] Sherrington C., Fairhall N.J., Wallbank G.K., Tiedemann A., Michaleff Z.A., Howard K., Clemson L., Hopewell S., Lamb S.E. (2019). Exercise for preventing falls in older people living in the community. Cochrane Database Syst. Rev..

[409] Smith C., Gold J., Ngo T.D., Sumpter C., Free C. (2015). Mobile phone-based interventions for improving contraception use. Cochrane Database Syst. Rev..

[410] Taylor G.M.J., Dalili M.N., Semwal M., Civljak M., Sheikh A., Car J. (2017). Internet-based interventions for smoking cessation. Cochrane Database Syst. Rev..

[411] Vinceti M., Filippini T., Del Giovane C., Dennert G., Zwahlen M., Brinkman M., Zeegers M.P.A., Horneber M., D’Amico R., Crespi C.M. (2018). Selenium for preventing cancer. Cochrane Database Syst. Rev..

[412] Willcox M.L., Price J., Scott S., Nicholson B.D., Stuart B., Roberts N.W., Allott H., Mubangizi V., Dumont A., Harnden A. (2020). Death audits and reviews for reducing maternal, perinatal and child mortality. Cochrane Database Syst. Rev..

[413] Wolfenden L., McCrabb S., Barnes C., O’Brien K.M., Ng K.W., Nathan N.K., Sutherland R., Hodder R.K., Tzelepis F., Nolan E. (2022). Strategies for enhancing the implementation of school-based policies or practices targeting diet, physical activity, obesity, tobacco or alcohol use. Cochrane Database Syst. Rev..

[414] Kaner E.F.S., Beyer F.R., Garnett C., Crane D., Brown J., Muirhead C., Redmore J., O’Donnell A., Newham J.J., de Vocht F. (2017). Personalised digital interventions for reducing hazardous and harmful alcohol consumption in community-dwelling populations. Cochrane Database Syst. Rev..

[415] Dennis C.L., Dowswell T. (2013). Psychosocial and psychological interventions for preventing postpartum depression. Cochrane Database Syst. Rev..

[416] Liddle S.D., Pennick V. (2015). Interventions for preventing and treating low-back and pelvic pain during pregnancy. Cochrane Database Syst. Rev..

[417] Moe-Byrne T., Brown J.V.E., McGuire W. (2016). Glutamine supplementation to prevent morbidity and mortality in preterm infants. Cochrane Database Syst. Rev..

[418] Hemmingsen B., Gimenez-Perez G., Mauricio D., Roqué i Figuls M., Metzendorf M.I., Richter B. (2017). Diet, physical activity or both for prevention or delay of type 2 diabetes mellitus and its associated complications in people at increased risk of developing type 2 diabetes mellitus. Cochrane Database Syst. Rev..

[419] Sosa C.G., Althabe F., Belizán J.M., Bergel E. (2015). Bed rest in singleton pregnancies for preventing preterm birth. Cochrane Database Syst. Rev..

[420] Kaner E.F.S., Beyer F.R., Muirhead C., Campbell F., Pienaar E.D., Bertholet N., Daeppen J.B., Saunders J.B., Burnand B. (2018). Effectiveness of brief alcohol interventions in primary care populations. Cochrane Database Syst. Rev..

[421] Brocklehurst P., Kujan O., O’Malley L., Ogden G.R., Shepherd S., Glenny A.-M. (2013). Screening programmes for the early detection and prevention of oral cancer. Cochrane Database Syst. Rev..

[422] Lamont T., Worthington H.V., Clarkson J.E., Beirne P.V. (2018). Routine scale and polish for periodontal health in adults. Cochrane Database Syst. Rev..

[423] Smith S.M., Cousins G., Clyne B., Allwright S., O’Dowd T. (2017). Shared care across the interface between primary and specialty care in management of long term conditions. Cochrane Database Syst. Rev..

[424] Amorim Adegboye A.R., Linne Y.M. (2013). Diet or exercise, or both, for weight reduction in women after childbirth. Cochrane Database Syst. Rev..

[425] Oliveira J.M., Allert R., East C.E. (2016). Vitamin A supplementation for postpartum women. Cochrane Database Syst. Rev..

[426] Lassi Z.S., Moin A., Bhutta Z.A. (2016). Zinc supplementation for the prevention of pneumonia in children aged 2 months to 59 months. Cochrane Database Syst. Rev..

[427] Karmali K.N., Persell S.D., Perel P., Lloyd-Jones D.M., Berendsen M.A., Huffman M.D. (2017). Risk scoring for the primary prevention of cardiovascular disease. Cochrane Database Syst. Rev..

[428] van Vilsteren M., van Oostrom S.H., de Vet H.C.W., Franche R.L., Boot C.R.L., Anema J.R. (2015). Workplace interventions to prevent work disability in workers on sick leave. Cochrane Database Syst. Rev..

[429] Imdad A., Ahmed Z., Bhutta Z.A. (2016). Vitamin A supplementation for the prevention of morbidity and mortality in infants one to six months of age. Cochrane Database Syst. Rev..

[430] Tzortziou Brown V., Underwood M., Mohamed N., Westwood O., Morrissey D. (2016). Professional interventions for general practitioners on the management of musculoskeletal conditions. Cochrane Database Syst. Rev..

[431] McCauley M.E., van den Broek N., Dou L., Othman M. (2015). Vitamin A supplementation during pregnancy for maternal and newborn outcomes. Cochrane Database Syst. Rev..

[432] Boyle R., Solberg L., Fiore M. (2014). Use of electronic health records to support smoking cessation. Cochrane Database Syst. Rev..

[433] Schwenger E.M., Tejani A.M., Loewen P.S. (2015). Probiotics for preventing urinary tract infections in adults and children. Cochrane Database Syst. Rev..

[434] Lavender T., Richens Y., Milan S.J., Smyth R.M.D., Dowswell T. (2013). Telephone support for women during pregnancy and the first six weeks postpartum. Cochrane Database Syst. Rev..

[435] De-Regil L.M., Jefferds M.E.D., Peña-Rosas J.P. (2017). Point-of-use fortification of foods with micronutrient powders containing iron in children of preschool and school-age. Cochrane Database Syst. Rev..

[436] Thomas D., Abramson M.J., Bonevski B., George J. (2017). System change interventions for smoking cessation. Cochrane Database Syst. Rev..

[437] Suchdev P.S., Peña-Rosas J.P., De-Regil L.M. (2015). Multiple micronutrient powders for home (point-of-use) fortification of foods in pregnant women. Cochrane Database Syst. Rev..

[438] Lindson N., Pritchard G., Hong B., Fanshawe T.R., Pipe A., Papadakis S. (2021). Strategies to improve smoking cessation rates in primary care. Cochrane Database Syst. Rev..

[439] Fair F.J., Ford G.L., Soltani H. (2019). Interventions for supporting the initiation and continuation of breastfeeding among women who are overweight or obese. Cochrane Database Syst. Rev..

[440] Garn J.V., Wilkers J.L., Meehan A.A., Pfadenhauer L.M., Burns J., Imtiaz R., Freeman M.C. (2022). Interventions to improve water, sanitation, and hygiene for preventing soil-transmitted helminth infection. Cochrane Database Syst. Rev..

[441] Grev J., Berg M., Soll R. (2018). Maternal probiotic supplementation for prevention of morbidity and mortality in preterm infants. Cochrane Database Syst. Rev..

[442] Das J.K., Hoodbhoy Z., Salam R.A., Bhutta A.Z., Valenzuela-Rubio N.G., Weise Prinzo Z., Bhutta Z.A. (2018). Lipid-based nutrient supplements for maternal, birth, and infant developmental outcomes. Cochrane Database Syst. Rev..

[443] Das J.K., Salam R.A., Hadi Y.B., Sadiq Sheikh S., Bhutta A.Z., Weise P.Z., Bhutta Z.A. (2019). Preventive lipid-based nutrient supplements given with complementary foods to infants and young children 6 to 23 months of age for health, nutrition, and developmental outcomes. Cochrane Database Syst. Rev..

[444] Baxter J.-A., Carducci B., Kamali M., Zlotkin S.H., Bhutta Z.A. (2022). Fortification of salt with iron and iodine versus fortification of salt with iodine alone for improving iron and iodine status. Cochrane Database Syst. Rev..

[445] Vonasek B., Ness T., Takwoingi Y., Kay A.W., van Wyk S.S., Ouellette L., Marais B.J., Steingart K.R., Mandalakas A.M. (2021). Screening tests for active pulmonary tuberculosis in children. Cochrane Database Syst. Rev..

[446] Viswanathan M., Kahwati L., Jahn B., Giger K., Dobrescu A.I., Hill C., Klerings I., Meixner J., Persad E., Teufer B. (2020). Universal screening for SARS-CoV-2 infection: A rapid review. Cochrane Database Syst. Rev..

