# Supplementary material for: Systematic Evaluation of How Indicators of Inequity and Disadvantage Are Measured and Reported in Population Health Evidence Syntheses
Source: Int J Environ Res Public Health. 2025 May 29;22(6):851. doi: 10.3390/ijerph22060851 (PMC12192879; doi:10.3390/ijerph22060851)
Supplement: Supplementary file 1 [file ijerph-22-00851-s001.zip › Suppl file S2 - Exclusion reasons.pdf]

Supplementary file S2. Types of excluded reviews with reasons

**Review type**	**Reason for exclusion**
Reviews of diagnostic accuracy	Reviews focused on the sensitivity and specificity of tests for detecting disease, rather than differences between population groups were excluded. Reviews of screening programmes with potential to examine social patterning in uptake or effectiveness were included.
Reviews of qualitative studies	Qualitative studies are designed to understand meaning [70]. The focus of the present study was discrepancies in health-related outcomes or intervention effects through quantitative subgroup analysis.
Reviews recorded as withdrawn	Review not available.
Methodological reviews	Reviews focused on the methods or processes of research (e.g., participant recruitment, retention, or randomisation, or different statistical methods) were excluded.
Scoping reviews	Scoping reviews were excluded as they did not include quantitative syntheses with potential for subgroup analyses.
Reviews of clinical intervention studies	Reviews of treatments for conditions or medical procedures were excluded. These included pharmacological interventions (e.g., medications for existing conditions, smoking cessation, weight loss), other types of treatment (e.g., emollients, skin treatments), and treatments/procedures undertaken in healthcare settings (e.g., surgical procedures, ultrasound, shockwave therapy, childbirth processes, acupuncture, dental procedures). Non-pharmacological weight loss interventions were potentially eligible given the high prevalence and social patterning of overweight/obesity [71], and potential for subgroup analysis.
Reviews of clinical populations	These included reviews of studies targeting people with a specific condition or syndrome, or where intervention recipients were 'patients' (already in a health care setting or taking medication).
Reviews of specific populations	These included reviews of specific (non-clinical) groups not defined by indicators relevant to PROGRESS-Plus (e.g., women with multiple births, people undertaking cosmetic procedures, airline passengers). Pregnant women were included unless there were additional conditions (e.g., overweight/obesity, gestational diabetes) given the large proportion of the population who bear children during their lifetime (e.g., ~40% UK population) and, therefore, potential for subgroup analysis [72].
Reviews including only low- and	Similar to others [52, 73], these were excluded as the LMIC focus introduces additional complexity through differences in the wider

middle-income countries (LMIC)	economic, geographical and health context between high-income countries (HIC) and LMICs [2], and related differences in equity measures [27].
Reviews of health service design, organisation of care, and healthcare professionals' practice	These were excluded if the primary outcomes did not include patient-level outcomes (e.g., only healthcare professional outcomes, healthcare utilisation) or focused on specific conditions (e.g., changes to patient care) as they did not align with population health. Reviews were eligible if they related to general health care changes/approaches that could improve care for the general population (not limited by condition or target group) and reported individual level health outcomes.
Reviews of 1-to-1 focused intervention studies	Reviews of studies excluded if the intervention aimed only to change an individual's behaviour. For example, reviews of individual counselling excluded, but family or group counselling might be eligible if aiming to influence a person's social environment (e.g., fostering social support, social connections)
