# Supplementary material for: Systematic Evaluation of How Indicators of Inequity and Disadvantage Are Measured and Reported in Population Health Evidence Syntheses
Source: Int J Environ Res Public Health. 2025 May 29;22(6):851. doi: 10.3390/ijerph22060851 (PMC12192879; doi:10.3390/ijerph22060851)
Supplement: Supplementary file 1 [file ijerph-22-00851-s001.zip › Suppl file S3 - Data extraction fields.pdf]

Supplementary file S3. Data extraction form fields

Variable name	Description (if appropriate)	Categories (if applicable)
ID	Cochrane ID and Year of review	
Cochrane review group code	Cochrane review group code	
Authors		
Title		
Health determinants - primary	Determinants of Health domain (Primary)	
Health determinants - secondary	Determinants of Health domain (Secondary)	
Include/exclude	Review included or excluded	Include; Exclude
Exclusion reason	If excluded, reason	1-to-1 delivery; Clinical intervention; Clinical population; Diagnostic accuracy; Health service design/organisation of care; LMIC only; Specific population
N studies included (total)	Number of studies included (total)	
N studies in quantitative synthesis	Number of studies included in quantitative synthesis	
Primary aim		
Population	Population as described in the review	
Population type	Population type (based on broad groupings)	
Primary outcome	Primary outcome(s) as described in the review	
Mention inequity/inequality or social patterning (in background, methods, etc.)	Did the introduction/rationale reference inequities, inequalities, or social patterning	Yes; No
PROGRESS/PROGRESS-Plus	Cited use of PROGRESS/Plus checklist	Yes; No
Intervention targeted/related to vulnerable group	Did the intervention target or relate to a vulnerable group	Yes; No
Planned sub-group analysis (any)		Yes; No
Completed sub-group analysis (any)		Yes; No
Subgroup analysis by		
Place		Planned (Yes/No/Not specified)
		Undertaken (Y/N)
		Var1
		Var1 Categories
		Var2
		Var2 Categories
Race/ethnicity		Planned (Yes/No/Not specified)
		Undertaken

Variable name	Description (if appropriate)	Categories (if applicable)
		Categories
Occupation		Planned (Yes/No/Not specified)
		Undertaken
		Categories
Gender/sex		Planned (Yes/No/Not specified)
		Undertaken
		Categories
Religion		Planned (Yes/No/Not specified)
		Undertaken
		Categories
Education		Planned (Yes/No/Not specified)
		Undertaken
		Categories
SES		Planned (Yes/No/Not specified)
		Undertaken
		Categories
Social capital		Planned (Yes/No/Not specified)
		Undertaken
		Categories
Personal characteristics		Planned (Yes/No/Not specified)
		Var1
		Var1 Categories
		Var2
		Var2 Categories
	Features of relationships	Planned (Yes/No/Not specified)
		Undertaken
		Categories
	Time-dependent relationships	Planned (Yes/No/Not specified)
		Undertaken
		Categories
