# Supplementary material for: Systematic Evaluation of How Indicators of Inequity and Disadvantage Are Measured and Reported in Population Health Evidence Syntheses
Source: Int J Environ Res Public Health. 2025 May 29;22(6):851. doi: 10.3390/ijerph22060851 (PMC12192879; doi:10.3390/ijerph22060851)
Supplement: Supplementary file 1 [file ijerph-22-00851-s001.zip › Suppl file S5 - Included studies.pdf]

Supplementary file S5. Characteristics of included reviews (n=363)

Study	Title	Population type	Determinants of health		N studies included		Mention inequity/ inequality/social patterning	Focus on vulnerable group	Sub-group analysis	
			Primary	Secondary	Total	Quant			Plan	Complete
**Did not plan nor complete subgroup analysis for any PROGRESS-Plus indicators**										
Abe 2016 [74]	Supplementation with multiple micronutrients for breastfeeding women for improving outcomes for the mother and baby	Pregnant women/Planning pregnancy/New mothers	Individual lifestyle factors		2	0	Y	N	N	N
Adler 2014 [75]	Reduced dietary salt for the prevention of cardiovascular disease	Adults	Individual lifestyle factors		8	8	N	N	N	N
Akl 2013 [76]	Educational games for health professionals	Healthcare professionals	Health care services		2	0	N	N	N	N
Al-Khudairy 2017 [77]	Vitamin C supplementation for the primary prevention of cardiovascular disease	Adults	Individual lifestyle factors		8	0	Y	N	N	N
Al-Khudairy 2017 [78]	Diet, physical activity and behavioural interventions for the treatment of overweight or obese adolescents aged 12 to 17 years	Adolescents	Individual lifestyle factors		44	39	Y	N	N	N
Allaouat 2020 [79]	Educational interventions for preventing lead poisoning in workers	Vulnerable population - Workers exposed to risk	Living and working conditions		4	4	Y	Y	N	N
Andras 2014 [80]	Screening for peripheral arterial disease	Adults	Health care services		0	0	N	N	N	N
Arikpo 2018 [81]	Educational interventions for improving primary caregiver complementary feeding practices for children aged 24 months and under	Parents/guardians/families	Individual lifestyle factors		23	15	Y	N	N	N
Baker 2015 [82]	Tailored interventions to address determinants of practice	Healthcare professionals	Health care services		32	15	N	N	N	N
Bala 2017 [83]	Mass media interventions for smoking cessation in adults	Adults	Individual lifestyle factors		7	0	N	N	N	N
Balogun 2016 [84]	Vitamin supplementation for preventing miscarriage	Pregnant women/Planning pregnancy/New mothers	Individual lifestyle factors	Health care services	40	40	N	N	N	N

Study	Title	Population type	Determinants of health		N studies included		Mention inequity/ inequality/social patterning	Focus on vulnerable group	Sub-group analysis	
			Primary	Secondary	Total	Quant			Plan	Complete
Bao 2020 [85]	Water for preventing urinary stones	Adults	Health care services	Individual lifestyle factors	1	1	N	N	N	N
Barlow 2016 [86]	Group-based parent training programmes for improving emotional and behavioural adjustment in young children	Parents/guardians/families	Education		24	23	N	N	N	N
Bergman 2021 [87]	Vaccines for preventing rotavirus diarrhoea: vaccines in use	Children	Health care services		60	60	Y	N	N	N
Bergwall 2022 [88]	High versus low-added sugar consumption for the primary prevention of cardiovascular disease	Adults	Individual lifestyle factors		21	20	Y	N	N	N
Bjelakovic 2014 [89]	Vitamin D supplementation for prevention of cancer in adults	Adults	Individual lifestyle factors		18	18	N	N	N	N
Bjelakovic 2014 [90]	Vitamin D supplementation for prevention of mortality in adults	Adults	Individual lifestyle factors		159	56	N	N	N	N
Buppasiri 2015 [91]	Calcium supplementation (other than for preventing or treating hypertension) for improving pregnancy and infant outcomes	Pregnant women/Planning pregnancy/New mothers	Individual lifestyle factors	Health care services	25	23	N	N	N	N
Burns 2021 [92]	International travel-related control measures to contain the COVID-19 pandemic: a rapid review	Mixed populations	Other		62	0	N	N	N	N
Cahill 2014 [93]	Workplace interventions for smoking cessation	Working adults	Individual lifestyle factors	Living and working conditions	57	57	N	N	N	N
Carberry 2014 [94]	Customised versus population-based growth charts as a screening tool for detecting small for gestational age infants in low-risk pregnant women	Pregnant women/Planning pregnancy/New mothers	Health care services		0	0	N	N	N	N
Carducci 2021 [95]	Zinc supplementation for improving pregnancy and infant outcome	Pregnant women/Planning pregnancy/New mothers	Individual lifestyle factors	Health care services	25	25	N	N	N	N
Carson-Chahhoud 2017 [96]	Mass media interventions for preventing smoking in young people	Young people	Education	Individual lifestyle factors	8	0	Y	N	N	N
Carson-Chahhoud 2019 [97]	Community pharmacy personnel interventions for smoking cessation	Healthcare professionals	Health care services		7	6	N	N	N	N

Study	Title	Population type	Determinants of health		N studies included		Mention inequity/ inequality/social patterning	Focus on vulnerable group	Sub-group analysis	
			Primary	Secondary	Total	Quant			Plan	Complete
Centeno 2019 [38]	Fortification of wheat and maize flour with folic acid for population health outcomes	Mixed populations	Agriculture and food production		10	5	Y	N	N	N
Cheetham 2021 [98]	Education and training for preventing sharps injuries and splash exposures in healthcare workers	Adults	Work environment	Health care services	7	6	N	N	N	N
Clar 2017 [99]	Low glycaemic index diets for the prevention of cardiovascular disease	Adults	Individual lifestyle factors		21	19	N	N	N	N
Clarke 2018 [100]	Community screening for visual impairment in older people	Older adults	Health care services		10	10	N	N	N	N
Colquitt 2016 [101]	Diet, physical activity, and behavioural interventions for the treatment of overweight or obesity in preschool children up to the age of 6 years	Infants/Children	Individual lifestyle factors		7	5	Y	N	N	N
Crepinsek 2020 [102]	Interventions for preventing mastitis after childbirth	Pregnant women/Planning pregnancy/New mothers	Health care services		10	9	Y	N	N	N
Coppo 2014 [103]	School policies for preventing smoking among young people	Students	Education		1	0	N	N	N	N
Davidson 2021 [104]	Probiotics for preventing gestational diabetes	Pregnant women/Planning pregnancy/New mothers	Individual lifestyle factors		7	7	N	N	N	N
De-Regil 2015 [105]	Effects and safety of periconceptional oral folate supplementation for preventing birth defects	Pregnant women/Planning pregnancy/New mothers	Individual lifestyle factors	Health care services	5	5	N	N	N	N
Delgado-Noguera 2015 [106]	Supplementation with long chain polyunsaturated fatty acids (LCPUFA) to breastfeeding mothers for improving child growth and development	Pregnant women/Planning pregnancy/New mothers	Individual lifestyle factors		8	8	N	N	N	N
Demicheli 2015 [107]	Vaccines for women for preventing neonatal tetanus	Women	Health care services		3	3	N	N	N	N

Study	Title	Population type	Determinants of health		N studies included		Mention inequity/ inequality/social patterning	Focus on vulnerable group	Sub-group analysis	
			Primary	Secondary	Total	Quant			Plan	Complete
Demicheli 2018 [108]	Vaccines for preventing influenza in the elderly	Older adults	Health care services		75	8	N	N	N	N
Demicheli 2018 [109]	Vaccines for preventing influenza in healthy adults	Adults	Health care services		110	94	N	N	N	N
Dyakova 2016 [110]	Systematic versus opportunistic risk assessment for the primary prevention of cardiovascular disease	Adults	Health care services		9	6	Y	N	N	N
Ebbert 2015 [111]	Interventions for smokeless tobacco use cessation	Smokers	Individual lifestyle factors		34	34	N	N	N	N
Evans 2017 [112]	Antioxidant vitamin and mineral supplements for preventing age-related macular degeneration	Adults	Individual lifestyle factors	Health care services	5	5	N	N	N	N
Faggiano 2014 [113]	Universal school-based prevention for illicit drug use	Students	Education	Individual lifestyle factors	51	25	N	N	N	N
Fanshawe 2017 [114]	Tobacco cessation interventions for young people	Young people	Education	Individual lifestyle factors	41	41	N	N	N	N
Faseru 2018 [115]	Enhancing partner support to improve smoking cessation	Mixed populations	Individual lifestyle factors		14	3	N	N	N	N
Fellmeth 2013 [116]	Educational and skills-based interventions for preventing relationship and dating violence in adolescents and young adults	Adolescents/Young people	Individual lifestyle factors	Social and community networks	41	33	N	N	N	N
Fernandez-Gaxiola 2019 [117]	Intermittent iron supplementation for reducing anaemia and its associated impairments in adolescent and adult menstruating women	Women	Individual lifestyle factors		25	25	Y	N	N	N
Ferri 2013 [118]	Media campaigns for the prevention of illicit drug use in young people	Young people	Individual lifestyle factors		23	13	N	N	N	N
Fiander 2015 [119]	Interventions to increase the use of electronic health information by healthcare practitioners to improve clinical practice and patient outcomes	Healthcare professionals	Health care services		6	0	Y	N	N	N
Filippini 2020 [120]	Green tea (Camellia sinensis) for the prevention of cancer	Adults	Individual lifestyle factors		142	142	N	N	N	N

Study	Title	Population type	Determinants of health		N studies included		Mention inequity/ inequality/social patterning	Focus on vulnerable group	Sub-group analysis	
			Primary	Secondary	Total	Quant			Plan	Complete
Flodgren 2017 [121]	Interventions to change the behaviour of health professionals and the organisation of care to promote weight reduction in children and adults with overweight or obesity	Mixed populations	Health care services		12	12	Y	N	N	N
Flodgren 2019 [122]	Local opinion leaders: effects on professional practice and healthcare outcomes	Healthcare professionals	Health care services		24	24	N	N	N	N
Forsetlund 2021 [123]	Continuing education meetings and workshops: effects on professional practice and healthcare outcomes	Healthcare professionals	Health care services		215	215	Y	N	N	N
Foster 2013 [124]	Remote and web 2.0 interventions for promoting physical activity	Adults	Individual lifestyle factors		30	11	Y	N	N	N
Foxcroft 2015 [125]	Social norms information for alcohol misuse in university and college students	Students	Individual lifestyle factors	Social and community networks	70	63	N	N	N	N
Frazer 2016 [126]	Legislative smoking bans for reducing harms from secondhand smoke exposure, smoking prevalence and tobacco consumption	Mixed populations	Individual lifestyle factors	Living and working conditions	77	0	Y	N	N	N
Frazer 2016 [127]	Impact of institutional smoking bans on reducing harms and secondhand smoke exposure	Mixed populations	Work environment	Individual lifestyle factors	17	11	N	N	N	N
Gagliardi 2019 [128]	Vaccines for preventing herpes zoster in older adults	Older adults	Health care services		24	24	N	N	N	N
Gartlehner 2013 [129]	Mammography in combination with breast ultrasonography versus mammography for breast cancer screening in women at average risk	Women	Health care services		8	8	N	N	N	N
Giguere 2020 [130]	Printed educational materials: effects on professional practice and healthcare outcomes	Healthcare professionals	Health care services		84	84	N	N	N	N
Goncalves-Bradley 2020 [131]	Mobile technologies to support healthcare provider to healthcare provider communication and management of care	Healthcare professionals	Health care services		19	19	Y	N	N	N
Grande 2016 [132]	Exercise prior to influenza vaccination for limiting influenza incidence and its related complications in adults	Adults	Individual lifestyle factors		6	6	Y	N	N	N

Study	Title	Population type	Determinants of health		N studies included		Mention inequity/ inequality/social patterning	Focus on vulnerable group	Sub-group analysis	
			Primary	Secondary	Total	Quant			Plan	Complete
Hafdi 2021 [133]	Multi-domain interventions for the prevention of dementia and cognitive decline	Older adults	Individual lifestyle factors		9	9	Y	N	N	N
Hameed 2020 [134]	Psychological therapies for women who experience intimate partner violence	Vulnerable population - Experience of abuse	Health care services		33	9	Y	Y	N	N
Harding 2017 [135]	Iodine supplementation for women during the preconception, pregnancy and postpartum period	Pregnant women/Planning pregnancy/New mothers	Individual lifestyle factors		14	11	N	N	N	N
Hartley 2013 [136]	Green and black tea for the primary prevention of cardiovascular disease	Adults	Individual lifestyle factors		11	6	N	N	N	N
Hartley 2013 [137]	Increased consumption of fruit and vegetables for the primary prevention of cardiovascular diseases	Adults	Individual lifestyle factors		10	7	N	N	N	N
Hartley 2014 [138]	Yoga for the primary prevention of cardiovascular disease	Adults	Individual lifestyle factors		11	10	N	N	N	N
Hartley 2015 [139]	Vitamin K for the primary prevention of cardiovascular disease	Adults	Individual lifestyle factors		1	0	Y	N	N	N
Hartley 2016 [140]	Dietary fibre for the primary prevention of cardiovascular disease	Adults	Individual lifestyle factors		23	17	N	N	N	N
Hartmann-Boyce 2018 [141]	Nicotine replacement therapy versus control for smoking cessation	Adults	Individual lifestyle factors	Health care services	18	18	N	N	N	N
Hartmann-Boyce 2021 [142]	Behavioural interventions for smoking cessation: an overview and network meta-analysis	Smokers	Individual lifestyle factors		312	312	Y	N	N	N
Hartmann-Boyce 2021 [143]	Interventions for preventing weight gain after smoking cessation	Smokers	Individual lifestyle factors		116	71	N	N	N	N
Hartmann-Boyce 2022 [144]	Electronic cigarettes for smoking cessation	Smokers	Individual lifestyle factors		29	2	N	N	N	N
Hefler 2017 [145]	Incentives for preventing smoking in children and adolescents	Children/Adolescents	Individual lifestyle factors		8	6	N	N	N	N
Hemila 2013 [146]	Vitamin C for preventing and treating the common cold	Mixed populations	Individual lifestyle factors	Health care services	44	44	N	N	N	N

Study	Title	Population type	Determinants of health		N studies included		Mention inequity/ inequality/social patterning	Focus on vulnerable group	Sub-group analysis	
			Primary	Secondary	Total	Quant			Plan	Complete
Hemila 2013 [147]	Vitamin C for preventing and treating pneumonia	Mixed populations	Individual lifestyle factors		3	3	N	N	N	N
Hoe 2018 [148]	Ergonomic interventions for preventing work-related musculoskeletal disorders of the upper limb and neck among office workers	Working adults	Work environment		17	6	N	N	N	N
Hofmeyr 2018 [149]	Calcium supplementation during pregnancy for preventing hypertensive disorders and related problems	Pregnant women/Planning pregnancy/New mothers	Individual lifestyle factors	Health care services	27	27	N	N	N	N
Hofmeyr 2019 [150]	Calcium supplementation commencing before or early in pregnancy, for preventing hypertensive disorders of pregnancy	Pregnant women/Planning pregnancy/New mothers	Health care services	Individual lifestyle factors	1	1	Y	N	N	N
Hopewell 2018 [151]	Multifactorial and multiple component interventions for preventing falls in older people living in the community	Older adults	Individual lifestyle factors		62	62	N	N	N	N
Huey 2020 [152]	Effects of oral vitamin D supplementation on linear growth and other health outcomes among children under five years of age	Infants/Children	Individual lifestyle factors		75	64	Y	N	N	N
Jaafar 2016 [153]	Effect of restricted pacifier use in breastfeeding term infants for increasing duration of breastfeeding	Infants	Individual lifestyle factors		3	2	N	N	N	N
Jackson 2022 [154]	Mindfulness for smoking cessation	Smokers	Individual lifestyle factors		27	19	N	N	N	N
Jacobson Vann 2018 [155]	Patient reminder and recall interventions to improve immunization rates	Mixed populations	Health care services		75	57	N	N	N	N
Jahanfar 2015 [156]	Effects of restricted caffeine intake by mother on fetal, neonatal and pregnancy outcomes	Pregnant women/Planning pregnancy/New mothers	Individual lifestyle factors		2	1	N	N	N	N
Jasani 2017 [157]	Long chain polyunsaturated fatty acid supplementation in infants born at term	Infants	Health care services		15	15	Y	N	N	N
Jawad 2019 [158]	Interventions using social networking sites to promote contraception in women of reproductive age	Women	Individual lifestyle factors		2	0	Y	N	N	N

Study	Title	Population type	Determinants of health		N studies included		Mention inequity/ inequality/social patterning	Focus on vulnerable group	Sub-group analysis	
			Primary	Secondary	Total	Quant			Plan	Complete
Jia 2014 [159]	Strategies for expanding health insurance coverage in vulnerable populations	Vulnerable population - Mixed	Health care services		2	0	Y	Y	N	N
Kalra 2021 [160]	Training healthcare providers to respond to intimate partner violence against women	Vulnerable population - Experience of abuse	Health care services		19	7	N	Y	N	N
Karsch-Volk 2014 [161]	Echinacea for preventing and treating the common cold	Mixed populations	Health care services		24	24	N	N	N	N
Kaufman 2018 [162]	Face-to-face interventions for informing or educating parents about early childhood vaccination	Mixed populations	Education	Health care services	10	10	Y	N	N	N
Keats 2019 [163]	Multiple-micronutrient supplementation for women during pregnancy	Pregnant women/Planning pregnancy/New mothers	Individual lifestyle factors	Health care services	21	20	N	N	N	N
Kelleher 2022 [164]	Skin care interventions in infants for preventing eczema and food allergy	Infants	Health care services		33	17	Y	N	N	N
Kelly 2017 [165]	Whole grain cereals for the primary or secondary prevention of cardiovascular disease	Adults	Individual lifestyle factors		9	8	N	N	N	N
Kendrick 2013 [166]	Parenting interventions for the prevention of unintentional injuries in childhood	Parents/guardians/f amilies	Other		22	13	Y	N	N	N
Krishnaratne 2022 [167]	Measures implemented in the school setting to contain the COVID-19 pandemic	Students, school stakeholders	Education		16	0	Y	N	N	N
Krogsboll 2019 [168]	General health checks in adults for reducing morbidity and mortality from disease	Adults	Health care services		17	11	N	N	N	N
Kuehnl 2019 [169]	Human resource management training of supervisors for improving health and well-being of employees	Working adults	Work environment		25	21	N	N	N	N
Kunzler 2020 [170]	Psychological interventions to foster resilience in healthcare professionals	Adults	Living and working conditions	Health care services	44	34	N	N	N	N
Kuster 2017 [171]	Computer-based versus in-person interventions for preventing and reducing stress in workers	Adults	Work environment		2	2	N	N	N	N
Lak 2020 [172]	Newborn screening for galactosaemia	Infants	Health care services		0	0	Y	N	N	N

Study	Title	Population type	Determinants of health		N studies included		Mention inequity/ inequality/social patterning	Focus on vulnerable group	Sub-group analysis	
			Primary	Secondary	Total	Quant			Plan	Complete
Lassi 2013 [173]	Folic acid supplementation during pregnancy for maternal health and pregnancy outcomes	Pregnant women/Planning pregnancy/New mothers	Individual lifestyle factors		31	31	N	N	N	N
Lawrenson 2015 [174]	Omega 3 fatty acids for preventing or slowing the progression of age-related macular degeneration	Mixed populations	Individual lifestyle factors		2	2	N	N	N	N
Law 2019 [175]	Psychological interventions for parents of children and adolescents with chronic illness	Vulnerable population - Caregivers	Health care services		47	47	Y	Y	N	N
Lindson 2019 [176]	Smoking reduction interventions for smoking cessation	Smokers	Individual lifestyle factors		51	51	N	N	N	N
Lindson-Hawley 2016 [177]	Interventions to reduce harm from continued tobacco use	Smokers	Individual lifestyle factors		24	24	N	N	N	N
Lissiman 2014 [178]	Garlic for the common cold	Mixed populations	Individual lifestyle factors		1	0	Y	N	N	N
Liu 2018 [179]	Mindfulness-based stress reduction for family carers of people with dementia	Vulnerable population - Caregivers	Social and community networks		5	5	N	Y	N	N
Livingstone 2013 [180]	Restorative justice conferencing for reducing recidivism in young offenders (aged 7 to 21)	Vulnerable population - Young offenders	Other		4	4	Y	Y	N	N
Livingstone-Banks 2019 [181]	Relapse prevention interventions for smoking cessation	Adults	Individual lifestyle factors		81	81	N	N	N	N
Livingstone-Banks 2019 [182]	Print-based self-help interventions for smoking cessation	Adults	Education	Individual lifestyle factors	75	75	N	N	N	N
Lopez 2016 [183]	Behavioral interventions for improving condom use for dual protection	Adults	Individual lifestyle factors		15	7	Y	N	N	N
Lopez 2013 [184]	Strategies for communicating contraceptive effectiveness	Mixed populations	Individual lifestyle factors		7	0	N	N	N	N
Lopez 2015 [185]	Education for contraceptive use by women after childbirth	Pregnant women/Planning pregnancy/New mothers	Health care services	Individual lifestyle factors	12	0	Y	N	N	N

Study	Title	Population type	Determinants of health		N studies included		Mention inequity/ inequality/social patterning	Focus on vulnerable group	Sub-group analysis	
			Primary	Secondary	Total	Quant			Plan	Complete
Lopez 2016 [186]	School-based interventions for improving contraceptive use in adolescents	Adolescents	Education	Individual lifestyle factors	21	11	Y	N	N	N
Lopez 2016 [187]	Brief educational strategies for improving contraception use in young people	Adults	Individual lifestyle factors		15	11	Y	N	N	N
Loveman 2015 [188]	Parent-only interventions for childhood overweight or obesity in children aged 5 to 11 years	Children	Individual lifestyle factors		20	14	Y	N	N	N
Luong Thanh 2016 [189]	Behavioural interventions to promote workers' use of respiratory protective equipment	Vulnerable population - Workers exposed to risk	Work environment		14	10	N	Y	N	N
Mendez-Sanchez 2023 [190]	Calcium and vitamin D for increasing bone mineral density in premenopausal women	Women	Individual lifestyle factors		7	4	N	N	N	N
Makrides 2014 [191]	Magnesium supplementation in pregnancy	Pregnant women/Planning pregnancy/New mothers	Individual lifestyle factors	Health care services	10	10	N	N	N	N
Marinho 2015 [192]	Fluoride gels for preventing dental caries in children and adolescents	Children/Adolescent s	Other		28	27	N	N	N	N
Marinho 2016 [193]	Fluoride mouthrinses for preventing dental caries in children and adolescents	Children/Adolescent s	Other		37	35	N	N	N	N
Martin 2015 [194]	Nut consumption for the primary prevention of cardiovascular disease	Adults	Individual lifestyle factors		5	5	Y	N	N	N
Maziak 2015 [195]	Interventions for waterpipe smoking cessation	Smokers	Individual lifestyle factors		9	7	N	N	N	N
McArthur 2018 [44]	Phonics training for English-speaking poor readers	Vulnerable population - Poor literacy	Education		14	14	Y	Y	N	N
McNeill 2017 [196]	Tobacco packaging design for reducing tobacco use	Mixed populations	Individual lifestyle factors		54	0	N	N	N	N
Mead 2017 [197]	Diet, physical activity and behavioural interventions for the treatment of overweight or obese children from the age of 6 to 11 years	Children	Individual lifestyle factors		20	55	Y	N	N	N

Study	Title	Population type	Determinants of health		N studies included		Mention inequity/ inequality/social patterning	Focus on vulnerable group	Sub-group analysis	
			Primary	Secondary	Total	Quant			Plan	Complete
Medley 2018 [198]	Interventions during pregnancy to prevent preterm birth: an overview of Cochrane systematic reviews	Pregnant women/Planning pregnancy/New mothers	Health care services		83	0	Y	N	N	N
Middleton 2018 [199]	Omega-3 fatty acid addition during pregnancy	Pregnant women/Planning pregnancy/New mothers	Individual lifestyle factors	Health care services	70	61	N	N	N	N
Miller 2013 [200]	Dietary supplements for preventing postnatal depression	Pregnant women/Planning pregnancy/New mothers	Individual lifestyle factors		2	2	N	N	N	N
Mischke 2013 [201]	Occupational safety and health enforcement tools for preventing occupational diseases and injuries	Working adults	Work environment		23	17	N	N	N	N
Montesinos-Guevara 2022 [202]	Vaccines for the common cold	Mixed populations	Health care services		1	0	N	N	N	N
Mosdol 2017 [46]	Targeted mass media interventions promoting healthy behaviours to reduce risk of non-communicable diseases in adult, ethnic minorities	Adults	Individual lifestyle factors		5	5	Y	N	N	N
Motuhifonua 2023 [203]	Antenatal dietary supplementation with myo-inositol for preventing gestational diabetes	Pregnant women/Planning pregnancy/New mothers	Health care services		7	7	Y	N	N	N
Muktabhant 2015 [204]	Diet or exercise, or both, for preventing excessive weight gain in pregnancy	Pregnant women/Planning pregnancy/New mothers	Individual lifestyle factors		65	49	N	N	N	N
Naude 2018 [205]	Effects of total fat intake on bodyweight in children	Children/Adolescent s	Individual lifestyle factors		24	21	Y	N	N	N
Ndikom 2014 [206]	Extra fluids for breastfeeding mothers for increasing milk production	Pregnant women/Planning pregnancy/New mothers	Individual lifestyle factors		1	0	N	N	N	N
Noone 2020 [207]	Video calls for reducing social isolation and loneliness in older people: a rapid review	Older adults	Social and community networks				N	N	N	N
Notley 2019 [208]	Incentives for smoking cessation	Smokers	Individual lifestyle factors		43	33	N	N	N	N

Study	Title	Population type	Determinants of health		N studies included		Mention inequity/ inequality/social patterning	Focus on vulnerable group	Sub-group analysis	
			Primary	Secondary	Total	Quant			Plan	Complete
Okolie 2020 [209]	Means restriction for the prevention of suicide on roads	Mixed populations	Other		0	0	Y	N	N	N
Okolie 2020 [210]	Means restriction for the prevention of suicide by jumping	Mixed populations	Other		18	12	Y	N	N	N
Ong 2019 [211]	Probiotics to prevent infantile colic	Infants	Health care services		6	6	N	N	N	N
Osborn 2013 [212]	Prebiotics in infants for prevention of allergy	Infants	Health care services		4	4	N	N	N	N
Osborn 2018 [213]	Infant formulas containing hydrolysed protein for prevention of allergic disease	Infants	Agriculture and food production		16	16	N	N	N	N
Pachito 2018 [214]	Workplace lighting for improving alertness and mood in daytime workers	Adults	Work environment		5	2	N	N	N	N
Palacios 2019 [215]	Regimens of vitamin D supplementation for women during pregnancy	Pregnant women/Planning pregnancy/New mothers	Health care services	Individual lifestyle factors	30	30	Y	N	N	N
Pantoja 2019 [216]	Manually-generated reminders delivered on paper: effects on professional practice and patient outcomes	Healthcare professionals	Health care services		57	57	Y	N	N	N
Pena-Rosas 2015 [217]	Daily oral iron supplementation during pregnancy	Pregnant women/Planning pregnancy/New mothers	Health care services	Individual lifestyle factors	61	44	N	N	N	N
Pena-Rosas 2015 [218]	Intermittent oral iron supplementation during pregnancy	Pregnant women/Planning pregnancy/New mothers	Individual lifestyle factors	Health care services	27	21	Y	N	N	N
Peer 2020 [219]	Screening for type 2 diabetes mellitus	Mixed populations	Health care services		1	1	N	N	N	N
Petrosino 2013 [220]	'Scared Straight' and other juvenile awareness programs for preventing juvenile delinquency	Vulnerable population - Young offfenders	Other		9	7	N	Y	N	N
Rees 2013 [221]	Selenium supplementation for the primary prevention of cardiovascular disease	Adults	Individual lifestyle factors		12	6	Y	N	N	N

Study	Title	Population type	Determinants of health		N studies included		Mention inequity/ inequality/social patterning	Focus on vulnerable group	Sub-group analysis	
			Primary	Secondary	Total	Quant			Plan	Complete
Rees 2019 [222]	Mediterranean-style diet for the primary and secondary prevention of cardiovascular disease	Adults	Individual lifestyle factors		30	30	N	N	N	N
Rees 2021 [223]	Vegan dietary pattern for the primary and secondary prevention of cardiovascular diseases	Adults	Individual lifestyle factors		13	13	Y	N	N	N
Reeves 2013 [224]	Interprofessional education: effects on professional practice and healthcare outcomes	Healthcare/social care professionals	Health care services		15	0	N	N	N	N
Reeves 2017 [225]	Interprofessional collaboration to improve professional practice and healthcare outcomes	Healthcare/social care professionals	Health care services		9	0	N	N	N	N
Richards 2013 [226]	Face-to-face interventions for promoting physical activity	Adults	Individual lifestyle factors		12	10	N	N	N	N
Richards 2013 [227]	Face-to-face versus remote and web 2.0 interventions for promoting physical activity	Adults	Individual lifestyle factors		1	1	N	N	N	N
Rivas 2015 [228]	Advocacy interventions to reduce or eliminate violence and promote the physical and psychosocial well-being of women who experience intimate partner abuse	Vulnerable population - Experience of abuse	Other		10	10	N	Y	N	N
Rivas 2019 [229]	A realist review of which advocacy interventions work for which abused women under what circumstances	Vulnerable population - Experience of abuse	Other		98	0	Y	Y	N	N
Robertson 2013 [230]	Non-pharmacological interventions for preventing venous insufficiency in a standing worker population	Working adults	Work environment	Living and working conditions	1	1	N	N	N	N
Rumbold 2015 [231]	Vitamin E supplementation in pregnancy	Pregnant women/Planning pregnancy/New mothers	Individual lifestyle factors		21	17	N	N	N	N
Rumbold 2015 [232]	Vitamin C supplementation in pregnancy	Pregnant women/Planning pregnancy/New mothers	Individual lifestyle factors		29	29	N	N	N	N
Rutjes 2018 [233]	Vitamin and mineral supplementation for maintaining cognitive function in cognitively healthy people in mid and late life	Adults	Individual lifestyle factors		28	27	Y	N	N	N

Study	Title	Population type	Determinants of health		N studies included		Mention inequity/ inequality/social patterning	Focus on vulnerable group	Sub-group analysis	
			Primary	Secondary	Total	Quant			Plan	Complete
Saeterdal 2014 [234]	Interventions aimed at communities to inform and/or educate about early childhood vaccination	Mixed populations	Health care services		2	0	Y	N	N	N
Salam 2015 [235]	Pyridoxine (vitamin B6) supplementation during pregnancy or labour for maternal and neonatal outcomes	Pregnant women/Planning pregnancy/New mothers	Health care services	Individual lifestyle factors	4	4	N	N	N	N
Sandall 2016 [236]	Midwife-led continuity models versus other models of care for childbearing women	Pregnant women/Planning pregnancy/New mothers	Health care services		15	15	N	N	N	N
Sangkomkamhang 2014 [237]	Hepatitis B vaccination during pregnancy for preventing infant infection	Pregnant women/Planning pregnancy/New mothers	Health care services		0	0	N	N	N	N
Santesso 2014 [238]	Hip protectors for preventing hip fractures in older people	Older adults	Health care services	Living and working conditions	19	19	N	N	N	N
Sauni 2015 [239]	Remediating buildings damaged by dampness and mould for preventing or reducing respiratory tract symptoms, infections and asthma	Vulnerable population - Substandard housing	Housing		12	12	N	Y	N	N
Schaafsma 2016 [240]	Pre-employment examinations for preventing injury, disease and sick leave in workers	Adults	Work environment		11	0	N	N	N	N
Schindler 2016 [241]	Polyunsaturated fatty acid supplementation in infancy for the prevention of allergy	Infants	Health care services		9	9	N	N	N	N
Schmucker 2022 [242]	Effects of a gluten-reduced or gluten-free diet for the primary prevention of cardiovascular disease	Adults	Individual lifestyle factors		4	4	Y	N	N	N
Scott 2019 [243]	Probiotics for preventing acute otitis media in children	Children/Adolescent s	Health care services	Individual lifestyle factors	17	16	Y	N	N	N
Shrestha 2018 [244]	Workplace interventions for reducing sitting at work	Working adults	Work environment		34	19	N	N	N	N
Soltan 2022 [245]	Community-based interventions for improving mental health in refugee children and adolescents in high-income countries	Vulnerable population - Refugee children/adolescent s	Other		38	3	Y	Y	N	N

Study	Title	Population type	Determinants of health		N studies included		Mention inequity/ inequality/social patterning	Focus on vulnerable group	Sub-group analysis	
			Primary	Secondary	Total	Quant			Plan	Complete
Staley 2021 [246]	Interventions targeted at women to encourage the uptake of cervical screening	Women	Individual lifestyle factors		70	69	N	N	N	N
Stead 2017 [247]	Group behaviour therapy programmes for smoking cessation	Adults	Individual lifestyle factors	Social and community networks	66	66	N	N	N	N
Tan 2020 [248]	Vitamin D supplementation for term breastfed infants to prevent vitamin D deficiency and improve bone health	Infants	Individual lifestyle factors		19	19	N	N	N	N
Tattan-Birch 2022 [249]	Heated tobacco products for smoking cessation and reducing smoking prevalence	Smokers	Individual lifestyle factors		13	10	N	N	N	N
Taylor 2021 [250]	Smoking cessation for improving mental health	Smokers	Individual lifestyle factors		102	102	Y	N	N	N
Thomas 2013 [251]	School-based programmes for preventing smoking	Children/Adolescent s	Individual lifestyle factors	Education	134	134	N	N	N	N
Thomas 2015 [252]	Family-based programmes for preventing smoking by children and adolescents	Mixed populations	Individual lifestyle factors	Social and community networks	27	14	N	N	N	N
Thomas 2018 [253]	Interventions to increase influenza vaccination rates of those 60 years and older in the community	Older adults	Health care services		61	61	N	N	N	N
Tieu 2017 [254]	Screening for gestational diabetes mellitus based on different risk profiles and settings for improving maternal and infant health	Pregnant women/Planning pregnancy/New mothers	Health care services		2	0	Y	N	N	N
Tikka 2017 [255]	Interventions to prevent occupational noise-induced hearing loss	Vulnerable population - Workers exposed to risk	Work environment		29	29	N	Y	N	N
Uphoff 2020 [256]	An overview of systematic reviews on mental health promotion, prevention, and treatment of common mental disorders for refugees, asylum seekers, and internally displaced persons	Vulnerable population - Refugees/asylum seekers	Other		38	0	Y	Y	N	N
Ussher 2019 [257]	Exercise interventions for smoking cessation	Adults	Individual lifestyle factors		24	23	N	N	N	N

Study	Title	Population type	Determinants of health		N studies included		Mention inequity/ inequality/social patterning	Focus on vulnerable group	Sub-group analysis	
			Primary	Secondary	Total	Quant			Plan	Complete
van den Brand 2017 [258]	Healthcare financing systems for increasing the use of tobacco dependence treatment	Smokers, healthcare professionals	Health care services	Individual lifestyle factors	6	5	N	N	N	N
Vaona 2018 [259]	E-learning for health professionals	Healthcare professionals	Health care services		16	16	N	N	N	N
Virgara 2021 [260]	Interventions in outside-school hours childcare settings for promoting physical activity amongst schoolchildren aged 4 to 12 years	Children	Education	Individual lifestyle factors	9	9	Y	N	N	N
Walsh 2019 [261]	Fluoride toothpastes of different concentrations for preventing dental caries	Mixed populations	Other		96	91	Y	N	N	N
Walsh 2022 [262]	Child protection training for professionals to improve reporting of child abuse and neglect	Vulnerable population - Experience of abuse	Health care services		11	9	Y	Y	N	N
Whittaker 2019 [263]	Mobile phone text messaging and app-based interventions for smoking cessation	Smokers	Individual lifestyle factors		26	26	Y	N	N	N
Wolfenden 2018 [264]	Strategies to improve the implementation of workplace-based policies or practices targeting tobacco, alcohol, diet, physical activity and obesity	Organisational stakeholders	Work environment		6	3	Y	N	N	N
Wolfenden 2020 [265]	Strategies to improve the implementation of healthy eating, physical activity and obesity prevention policies, practices or programmes within childcare services	Healthcare/social care professionals	Education		21	12	Y	N	N	N
Yaacob 2014 [266]	Powered versus manual toothbrushing for oral health	Mixed populations	Other		56	51	N	N	N	N
Yonemoto 2021 [267]	Schedules for home visits in the early postpartum period	Pregnant women/Planning pregnancy/New mothers	Health care services		17	15	N	N	N	N
Young 2015 [268]	Aerobic exercise to improve cognitive function in older people without known cognitive impairment	Older adults	Individual lifestyle factors		12	12	N	N	N	N
**Planned but did not complete subgroup analysis for any PROGRESS-Plus indicators**										

Study	Title	Population type	Determinants of health		N studies included		Mention inequity/ inequality/social patterning	Focus on vulnerable group	Sub-group analysis	
			Primary	Secondary	Total	Quant			Plan	Complete
Abdel-Aleem 2016 [269]	Mobile clinics for women's and children's health	Mixed populations	Health care services		2	2	Y	N	Y	N
Abdullahi 2020 [270]	Improving vaccination uptake among adolescents	Children/Adolescent s	Health care services	Individual lifestyle factors	16	16	Y	N	Y	N
Ammenwerth 2021 [271]	Adult patient access to electronic health records	Adults	Health care services		10	2	Y	N	Y	N
Anderson 2015 [272]	Community coalition-driven interventions to reduce health disparities among racial and ethnic minority populations	Vulnerable population - Ethnic minorities	Social and community networks		58	58	Y	Y	Y	N
Arora 2022 [273]	School dental screening programmes for oral health	Children/Adolescent s	Education	Other	8	8	Y	N	Y	N
Baker 2015 [274]	Community wide interventions for increasing physical activity	Mixed populations	Individual lifestyle factors		33	0	Y	N	Y	N
Baker 2016 [37]	Interventions for preventing abuse in the elderly	Older adults	Other		7	0	Y	N	Y	N
Barlow 2014 [275]	Group-based parent training programmes for improving parental psychosocial health	Adults	Education		48	48	N	N	Y	N
Bauer 2018 [276]	Interventions for preventing occupational irritant hand dermatitis	Working adults	Living and working conditions		9	7	N	N	Y	N
Borrie 2015 [277]	Interventions for the cessation of non-nutritive sucking habits in children	Children/Adolescent s	Individual lifestyle factors		6	2	N	N	Y	N
Burns 2019 [278]	Interventions to reduce ambient particulate matter air pollution and their effect on health	Mixed populations	Living and working conditions		42	0	Y	N	Y	N
Byber 2021 [279]	Humidification of indoor air for preventing or reducing dryness symptoms or upper respiratory infections in educational settings and at the workplace	Adults	Education	Living and working conditions	12	0	Y	N	Y	N
Catling 2015 [280]	Group versus conventional antenatal care for women	Pregnant women/Planning pregnancy/New mothers	Health care services			4	N	N	Y	N

Study	Title	Population type	Determinants of health		N studies included		Mention inequity/ inequality/social patterning	Focus on vulnerable group	Sub-group analysis	
			Primary	Secondary	Total	Quant			Plan	Complete
Chaithongwongwatthana 2015 [281]	Pneumococcal vaccination during pregnancy for preventing infant infection	Pregnant women/Planning pregnancy/New mothers	Health care services		7	6	Y	N	Y	N
Chastin 2021 [282]	Interventions for reducing sedentary behaviour in community-dwelling older adults	Older adults	Individual lifestyle factors		7	7	Y	N	Y	N
Chen 2018 [283]	Non-clinical interventions for reducing unnecessary caesarean section	Pregnant women/Planning pregnancy/New mothers	Health care services		29	29	Y	N	Y	N
Clar 2015 [284]	Influenza vaccines for preventing cardiovascular disease	Adults	Health care services		9	7	N	N	Y	N
Cooper 2013 [285]	Primary school-based behavioural interventions for preventing caries	Children	Education	Other	4	0	Y	N	Y	N
Coren 2016 [40]	Interventions for promoting reintegration and reducing harmful behaviour and lifestyles in street-connected children and young people	Vulnerable population - Homeless	Other		13	10	Y	Y	Y	N
Crockett 2018 [286]	Nutritional labelling for healthier food or non-alcoholic drink purchasing and consumption	Mixed populations	Agriculture and food production	Individual lifestyle factors	28	17	Y	N	Y	N
Das 2019 [41]	Food fortification with multiple micronutrients: impact on health outcomes in general population	Mixed populations	Agriculture and food production		43	43	Y	N	Y	N
Desapriya 2014 [287]	Vision screening of older drivers for preventing road traffic injuries and fatalities	Older adults	Other		0	0	N	N	Y	N
Eaton 2019 [288]	Effectiveness of provision of animal-source foods for supporting optimal growth and development in children 6 to 59 months of age	Infants/Children	Agriculture and food production		6	0	Y	N	Y	N
Edwards 2013 [289]	Personalised risk communication for informed decision making about taking screening tests	Adults	Health care services		41	38	N	N	Y	N
Ejere 2015 [290]	Face washing promotion for preventing active trachoma	Vulnerable population - Trachoma endemic area	Water and sanitation		2	0	Y	Y	Y	N

Study	Title	Population type	Determinants of health		N studies included		Mention inequity/ inequality/social patterning	Focus on vulnerable group	Sub-group analysis	
			Primary	Secondary	Total	Quant			Plan	Complete
Els 2020 [291]	Random drug and alcohol testing for preventing injury in workers	Adults	Work environment		1	0	Y	N	Y	N
Fanshawe 2019 [292]	Competitions for smoking cessation	Mixed populations	Individual lifestyle factors		20	20	N	N	Y	N
Freak-Poli 2020 [293]	Workplace pedometer interventions for increasing physical activity	Working adults	Individual lifestyle factors	Work environmen t	14	14	N	N	Y	N
Garcia-Casal 2018 [42]	Fortification of maize flour with iron for controlling anaemia and iron deficiency in populations	Mixed populations	Agriculture and food production		5	3	Y	N	Y	N
Gates 2019 [294]	Computerised cognitive training for maintaining cognitive function in cognitively healthy people in midlife	Adults	Individual lifestyle factors		1	0	Y	N	Y	N
Gates 2020 [295]	Computerised cognitive training for 12 or more weeks for maintaining cognitive function in cognitively healthy people in late life	Older adults	Individual lifestyle factors		8	8	Y	N	Y	N
Gibson 2018 [296]	Welfare-to-work interventions and their effects on the mental and physical health of lone parents and their children	Vulnerable population - lone parents with social welfare support	General socio-economic		12	12	Y	Y	Y	N
Gillen 2017 [297]	Interventions for prevention of bullying in the workplace	Adults	Work environment		5	2	N	N	Y	N
Gonzalez-Fraile 2021 [298]	Remotely delivered information, training and support for informal caregivers of people with dementia	Vulnerable population - Caregivers	Other		26	25	Y	Y	Y	N
Goyder 2015 [299]	Email for clinical communication between healthcare professionals	Healthcare professionals	Health care services		1	0	N	N	Y	N
Grande 2020 [300]	Exercise versus no exercise for the occurrence, severity, and duration of acute respiratory infections	Mixed populations	Individual lifestyle factors		14	12	N	N	Y	N
Gulani 2014 [301]	Zinc supplements for preventing otitis media	Mixed populations	Individual lifestyle factors	Health care services	12	10	Y	N	Y	N
Harrod 2014 [302]	Interventions for primary prevention of suicide in university and other post-secondary educational settings	Students	Education		5	3	N	N	Y	N
Hartley 2014 [303]	Tai chi for primary prevention of cardiovascular disease	Adults	Individual lifestyle factors		13	0	N	N	Y	N

Study	Title	Population type	Determinants of health		N studies included		Mention inequity/ inequality/social patterning	Focus on vulnerable group	Sub-group analysis	
			Primary	Secondary	Total	Quant			Plan	Complete
Hartley 2015 [304]	Qigong for the primary prevention of cardiovascular disease	Adults	Individual lifestyle factors		11	0	N	N	Y	N
Horvat 2014 [305]	Cultural competence education for health professionals	Healthcare professionals	Health care services		5	0	Y	N	Y	N
Hult 2020 [306]	Health-improving interventions for obtaining employment in unemployed job seekers	Vulnerable population - Unemployed adults	Unemployment		15	15	Y	Y	Y	N
Husk 2016 [43]	Participation in environmental enhancement and conservation activities for health and well-being in adults: a review of quantitative and qualitative evidence	Adults	Individual lifestyle factors		19	7	Y	N	Y	N
Iheozor-Ejiofor 2015 [307]	Water fluoridation for the prevention of dental caries	Mixed populations	Water and sanitation		155	107	Y	N	Y	N
Kendrick 2014 [308]	Exercise for reducing fear of falling in older people living in the community	Older adults	Individual lifestyle factors		30	29	Y	N	Y	N
Kew 2017 [309]	Asthma education for school staff	Teachers	Education		5	5	Y	N	Y	N
Lhachimi 2020 [31]	Taxation of the fat content of foods for reducing their consumption and preventing obesity or other adverse health outcomes	Mixed populations	Agriculture and food production		2	0	Y	N	Y	N
Luger 2019 [310]	Work-break schedules for preventing musculoskeletal symptoms and disorders in healthy workers	Working adults	Work environment		6	6	Y	N	Y	N
Lumbiganon 2016 [311]	Antenatal breastfeeding education for increasing breastfeeding duration	Pregnant women/Planning pregnancy/New mothers	Health care services		24	20	N	N	Y	N
MacArthur 2018 [51]	Individual-, family-, and school-level interventions targeting multiple risk behaviours in young people	Children/Adolescents	Individual lifestyle factors		70	54	Y	N	Y	N
Manser 2013 [312]	Screening for lung cancer	Adults	Health care services		9	9	N	N	Y	N
Martin 2018 [313]	Physical activity, diet and other behavioural interventions for improving cognition and school achievement in children and	Children/Adolescents	Individual lifestyle factors		18	17	Y	N	Y	N

Study	Title	Population type	Determinants of health		N studies included		Mention inequity/ inequality/social patterning	Focus on vulnerable group	Sub-group analysis	
			Primary	Secondary	Total	Quant			Plan	Complete
	adolescents with obesity or overweight									
Marx 2017 [35]	Later school start times for supporting the education, health, and well-being of high school students	Students	Education		11	2	Y	N	Y	N
Mastellos 2014 [314]	Transtheoretical model stages of change for dietary and physical exercise modification in weight loss management for overweight and obese adults	Adults	Individual lifestyle factors		3	0	N	N	Y	N
McLaren 2016 [36]	Population-level interventions in government jurisdictions for dietary sodium reduction	Mixed populations	Agriculture and food production		15	10	Y	N	Y	N
Morgan 2020 [45]	Caregiver involvement in interventions for improving children's dietary intake and physical activity behaviors	Parents/guardians/families	Individual lifestyle factors		23	16	Y	N	Y	N
Mulvaney 2015 [315]	Cycling infrastructure for reducing cycling injuries in cyclists	Mixed populations	Other		21	7	Y	N	Y	N
Munn 2020 [316]	Rinse-free hand wash for reducing absenteeism among preschool and school children	Children/Adolescents	Education		19	14	N	N	Y	N
Murtagh 2020 [317]	Interventions outside the workplace for reducing sedentary behaviour in adults under 60 years of age	Adults	Individual lifestyle factors		24	12	Y	N	Y	N
Naghieh 2015 [318]	Organisational interventions for improving wellbeing and reducing work-related stress in teachers	Teachers	Work environment	Living and working conditions	4	0	N	N	Y	N
Norhayati 2017 [319]	Influenza vaccines for preventing acute otitis media in infants and children	Infants/Children	Health care services		11	11	N	N	Y	N
Ojha 2020 [320]	Education of family members to support weaning to solids and nutrition in later infancy in term-born infants	Parents/guardians/families	Education	Individual lifestyle factors	21	21	Y	N	Y	N
O'Mahony 2017 [321]	Interventions for raising breast cancer awareness in women	Women	Health care services		2	0	N	N	Y	N
Oringanje 2016 [322]	Interventions for preventing unintended pregnancies among adolescents	Adolescents	Individual lifestyle factors	Education	53	53	Y	N	Y	N

Study	Title	Population type	Determinants of health		N studies included		Mention inequity/ inequality/social patterning	Focus on vulnerable group	Sub-group analysis	
			Primary	Secondary	Total	Quant			Plan	Complete
Orton 2016 [323]	School-based education programmes for the prevention of unintentional injuries in children and young people	Children/Adolescent s	Education		27	3	Y	N	Y	N
Padhani 2021 [324]	Vitamin C supplementation for prevention and treatment of pneumonia	Mixed populations	Individual lifestyle factors		5	0	Y	N	Y	N
Palmer 2020 [325]	Targeted client communication via mobile devices for improving maternal, neonatal, and child health	Pregnant women/Planning pregnancy/New mothers	Health care services		48	27	Y	N	Y	N
Palmer 2020 [326]	Targeted client communication via mobile devices for improving sexual and reproductive health	Mixed populations	Individual lifestyle factors		64	33	Y	N	Y	N
Parry 2019 [327]	Workplace interventions for increasing standing or walking for decreasing musculoskeletal symptoms in sedentary workers	Working adults	Work environment		10	8	Y	N	Y	N
Pega 2013 [32]	In-work tax credits for families and their impact on health status in adults	Working adults	General socio-economic		5	0	Y	N	Y	N
Petkovic 2021 [48]	Behavioural interventions delivered through interactive social media for health behaviour change, health outcomes, and health equity in the adult population	Adults	Individual lifestyle factors		88	80	Y	N	Y	N
Pfindern 2020 [33]	Taxation of unprocessed sugar or sugar-added foods for reducing their consumption and preventing obesity or other adverse health outcomes	Mixed populations	Agriculture and food production	Individual lifestyle factors	1	0	Y	N	Y	N
Pizarro 2022 [328]	Workplace interventions to reduce the risk of SARS-CoV-2 infection outside of healthcare settings	Adults	Work environment		1	1	Y	N	Y	N
Poorolajal 2016 [329]	Booster dose vaccination for preventing hepatitis B	Adults	Health care services		0	0	N	N	Y	N
Posadzki 2016 [330]	Automated telephone communication systems for preventive healthcare and management of long-term conditions	Adults	Health care services		132	132	Y	N	Y	N
Riggs 2019 [331]	Interventions with pregnant women, new mothers and other primary caregivers for preventing early childhood caries	Pregnant women/Planning pregnancy/New mothers	Health care services		17	14	Y	N	Y	N

Study	Title	Population type	Determinants of health		N studies included		Mention inequity/ inequality/social patterning	Focus on vulnerable group	Sub-group analysis	
			Primary	Secondary	Total	Quant			Plan	Complete
Sanchez 2016 [332]	Sun protection for preventing basal cell and squamous cell skin cancers	Mixed populations	Individual lifestyle factors		1	0	N	N	Y	N
Salam 2015 [333]	Impact of Haemophilus influenzae type B (Hib) and viral influenza vaccinations in pregnancy for improving maternal, neonatal and infant health outcomes	Pregnant women/Planning pregnancy/New mothers	Health care services		2	2	Y	N	Y	N
Schmidt 2020 [334]	Screening strategies for hypertension	Mixed populations	Health care services		0	0	Y	N	Y	N
Shepherd 2017 [335]	Combined diet and exercise interventions for preventing gestational diabetes mellitus	Pregnant women/Planning pregnancy/New mothers	Individual lifestyle factors		23	23	N	N	Y	N
Siegfried 2014 [336]	Restricting or banning alcohol advertising to reduce alcohol consumption in adults and adolescents	Mixed populations	Living and working conditions	Individual lifestyle factors	4	4	Y	N	Y	N
Silva 2022 [337]	Collective leadership to improve professional practice, healthcare outcomes and staff well-being	Healthcare professionals	Health care services		3	3	Y	N	Y	N
Slanger 2016 [338]	Person-directed, non-pharmacological interventions for sleepiness at work and sleep disturbances caused by shift work	Adults	Work environment	Living and working conditions	72	13	N	N	Y	N
Smith 2016 [339]	Early additional food and fluids for healthy breastfed full-term infants	Pregnant women/Planning pregnancy/New mothers	Individual lifestyle factors		11	11	y	N	Y	N
Steed 2019 [340]	Community pharmacy interventions for health promotion: effects on professional practice and health outcomes	Healthcare professionals	Health care services		58	25	Y	N	Y	N
Strobel 2022 [341]	Family-centred interventions for Indigenous early childhood well-being by primary healthcare services	Parents/guardians/families	Health care services	Social and community networks	19	11	Y	N	Y	N
Takahashi 2017 [342]	Fluoride supplementation (with tablets, drops, lozenges or chewing gum) in pregnant women for preventing dental caries in the primary teeth of their children	Pregnant women/Planning pregnancy/New mothers	Individual lifestyle factors	Other	1	1	Y	N	Y	N

Study	Title	Population type	Determinants of health		N studies included		Mention inequity/ inequality/social patterning	Focus on vulnerable group	Sub-group analysis	
			Primary	Secondary	Total	Quant			Plan	Complete
Tasnim 2020 [343]	Effect of alcohol on blood pressure	Adults	Individual lifestyle factors		32	32	Y	N	Y	N
Thomson 2013 [344]	Housing improvements for health and associated socio-economic outcomes	Vulnerable population - Substandard housing	Housing		39	19	Y	Y	Y	N
Tieu 2017 [345]	Dietary advice interventions in pregnancy for preventing gestational diabetes mellitus	Pregnant women/Planning pregnancy/New mothers	Individual lifestyle factors		11	11	Y	N	Y	N
Treanor 2019 [346]	Psychosocial interventions for informal caregivers of people living with cancer	Vulnerable population - Caregivers	Other		19	15	Y	Y	Y	N
Valentine 2019 [347]	Families and Schools Together (FAST) for improving outcomes for children and their families	Parents/guardians/families	Education	Social and community networks	10	9	Y	N	Y	N
van der Molen 2018 [348]	Interventions to prevent injuries in construction workers	Working adults	Work environment		17	9	Y	N	Y	N
van Urk 2014 [349]	Centre-based day care for children younger than five years of age in high-income countries	Infants/Children	Education		1	0	Y	N	Y	N
Vaona 2017 [350]	Training interventions for improving telephone consultation skills in clinicians	Healthcare professionals	Health care services		1	0	N	N	Y	N
Vijayaraghavan 2020 [351]	Interventions to reduce tobacco use in people experiencing homelessness	Vulnerable population - Homeless	Individual lifestyle factors		10	10	Y	Y	Y	N
Walsh 2015 [352]	School-based education programmes for the prevention of child sexual abuse	Children/Adolescents	Education		24	18	N	N	Y	N
Ward 2020 [353]	Mobility management to prevent, reduce, or delay driving a car in teenagers	Adolescents	Individual lifestyle factors	Other	2	2	N	N	Y	N
Winokur 2014 [354]	Kinship care for the safety, permanency, and well-being of children removed from the home for maltreatment	Vulnerable population - Experience of abuse	Social and community networks		46	46	N	Y	Y	N
Worthington 2019 [355]	Home use of interdental cleaning devices, in addition to toothbrushing, for preventing and controlling	Mixed populations	Other		36	8	Y	N	Y	N

Study	Title	Population type	Determinants of health		N studies included		Mention inequity/ inequality/social patterning	Focus on vulnerable group	Sub-group analysis	
			Primary	Secondary	Total	Quant			Plan	Complete
	periodontal diseases and dental caries									
Yakoob 2016 [356]	Vitamin D supplementation for preventing infections in children under five years of age	Infants/Children	Individual lifestyle factors		4	4	Y	N	Y	N
Yang 2015 [357]	Screening for nasopharyngeal cancer	Mixed populations	Health care services		0	0	N	N	Y	N
Yeung 2015 [358]	Fluoridated milk for preventing dental caries	Mixed populations	Agriculture and food production		1	1	Y	N	Y	N
**Completed subgroup analysis by 1 or more PROGRESS-Plus indicator**										
Abdelhamid 2018 [359]	Polyunsaturated fatty acids for the primary and secondary prevention of cardiovascular disease	Adults	Individual lifestyle factors		49	48	N	N	Y	Y
Allaf 2021 [360]	Intermittent fasting for the prevention of cardiovascular disease	Adults	Individual lifestyle factors		18	18	N	N	Y	Y
Arbyn 2018 [361]	Prophylactic vaccination against human papillomaviruses to prevent cervical cancer and its precursors	Women	Health care services		26	26	Y	N	Y	Y
Arditi 2017 [362]	Computer-generated reminders delivered on paper to healthcare professionals: effects on professional practice and healthcare outcomes	Healthcare professionals	Health care services		35	34	N	N	Y	Y
Avenell 2014 [363]	Vitamin D and vitamin D analogues for preventing fractures in post-menopausal women and older men	Older adults	Individual lifestyle factors	Health care services	53	52	N	N	Y	Y
Balogun 2016 [364]	Interventions for promoting the initiation of breastfeeding	Women	Education	Individual lifestyle factors	28	28	Y	N	Y	Y
Bauza 2023 [365]	Interventions to improve sanitation for preventing diarrhoea	Mixed populations	Water and sanitation		51	50	Y	N	Y	Y
Behbod 2018 [366]	Family and carer smoking control programmes for reducing children's exposure to environmental tobacco smoke	Parents/guardians/families	Individual lifestyle factors	Living and working conditions	78	0	N	N	Y	Y
Brand 2022 [367]	Replacing salt with low-sodium salt substitutes (LSSS) for cardiovascular health in adults, children and pregnant women	Adults	Individual lifestyle factors		26	25	N	N	Y	Y

Study	Title	Population type	Determinants of health		N studies included		Mention inequity/ inequality/social patterning	Focus on vulnerable group	Sub-group analysis	
			Primary	Secondary	Total	Quant			Plan	Complete
Brown 2019 [34]	Interventions for preventing obesity in children	Children/Adolescents	Individual lifestyle factors		153	153	Y	N	Y	Y
Chamberlain 2017 [39]	Psychosocial interventions for supporting women to stop smoking in pregnancy	Pregnant women/Planning pregnancy/New mothers	Individual lifestyle factors		102	88	Y	N	Y	Y
Clasen 2015 [368]	Interventions to improve water quality for preventing diarrhoea	Mixed populations	Water and sanitation		55	52	N	N	Y	Y
Cormick 2022 [369]	Calcium supplementation for prevention of primary hypertension	Mixed populations	Individual lifestyle factors		20	18	Y	N	Y	Y
da Silva Lopes 2021 [370]	Nutrition-specific interventions for preventing and controlling anaemia throughout the life cycle: an overview of systematic reviews	Mixed populations	Individual lifestyle factors				Y	N	Y	Y
Di Pietrantonj 2021 [371]	Vaccines for measles, mumps, rubella, and varicella in children	Children	Health care services		138	138	N	N	Y	Y
Ejemot-Nwadiaro 2021 [372]	Hand-washing promotion for preventing diarrhoea	Mixed populations	Other		29	20	Y	N	Y	Y
Gavine 2022 [373]	Support for healthy breastfeeding mothers with healthy term babies	Pregnant women/Planning pregnancy/New mothers	Health care services		116	103	Y	N	Y	Y
Gilligan 2019 [374]	Family-based prevention programmes for alcohol use in young people	Parents/guardians/families	Individual lifestyle factors	Social and community networks	46	31	Y	N	Y	Y
Graudal 2020 [375]	Effects of low sodium diet versus high sodium diet on blood pressure, renin, aldosterone, catecholamines, cholesterol, and triglyceride	Mixed populations	Individual lifestyle factors		195	195	N	N	Y	Y
Griffith 2020 [376]	Interventions to prevent women from developing gestational diabetes mellitus: an overview of Cochrane Reviews	Pregnant women/Planning pregnancy/New mothers	Health care services	Individual lifestyle factors	22	11	Y	N	Y	Y
He 2013 [377]	Effect of longer-term modest salt reduction on blood pressure	Adults	Individual lifestyle factors		34	34	Y	N	Y	Y
Hodder 2020 [378]	Interventions for increasing fruit and vegetable consumption in children aged five years and under	Mixed populations	Individual lifestyle factors		80	38	Y	N	Y	Y

Study	Title	Population type	Determinants of health		N studies included		Mention inequity/ inequality/social patterning	Focus on vulnerable group	Sub-group analysis	
			Primary	Secondary	Total	Quant			Plan	Complete
Hollands 2015 [379]	Portion, package or tableware size for changing selection and consumption of food, alcohol and tobacco	Mixed populations	Individual lifestyle factors		83	70	Y	N	Y	Y
Hollands 2019 [380]	Altering the availability or proximity of food, alcohol, and tobacco products to change their selection and consumption	Mixed populations	Living and working conditions	Individual lifestyle factors	24	23	Y	N	Y	Y
Hombali 2019 [30]	Fortification of staple foods with vitamin A for vitamin A deficiency	Mixed populations	Agriculture and food production		10	10	Y	N	Y	Y
Hooper 2015 [381]	Effects of total fat intake on body weight	Mixed populations	Individual lifestyle factors		31	29	Y	N	Y	Y
Hooper 2018 [382]	Omega-6 fats for the primary and secondary prevention of cardiovascular disease	Adults	Individual lifestyle factors		19	17	Y	N	Y	Y
Hooper 2020 [383]	Reduction in saturated fat intake for cardiovascular disease	Adults	Individual lifestyle factors		15	15	Y	N	Y	Y
Hooper 2020 [384]	Effects of total fat intake on body fatness in adults	Adults	Individual lifestyle factors		37	37	Y	N	Y	Y
Imdad 2022 [385]	Vitamin A supplementation for preventing morbidity and mortality in children from six months to five years of age	Infants/Children	Health care services		47	42	Y	N	Y	Y
Jefferson 2018 [386]	Vaccines for preventing influenza in healthy children	Infants/Children	Health care services		77	42	N	N	Y	Y
Jefferson 2023 [387]	Physical interventions to interrupt or reduce the spread of respiratory viruses	Mixed populations	Other		78	43	N	N	Y	Y
Kristjansson 2015 [388]	Food supplementation for improving the physical and psychosocial health of socio-economically disadvantaged children aged three months to five years	Children	Individual lifestyle factors		32	26	Y	N	Y	Y
Lassi 2020 [389]	Zinc supplementation for the promotion of growth and prevention of infections in infants less than six months of age	Infants/Children	Health care services		8	5	Y	N	Y	Y

Study	Title	Population type	Determinants of health		N studies included		Mention inequity/ inequality/social patterning	Focus on vulnerable group	Sub-group analysis	
			Primary	Secondary	Total	Quant			Plan	Complete
Lee 2021 [390]	Walking for hypertension	Adults	Individual lifestyle factors		73	73	N	N	Y	Y
Low 2016 [391]	Daily iron supplementation for improving anaemia, iron status and health in menstruating women	Women	Individual lifestyle factors		67	67	Y	N	Y	Y
Low 2016 [392]	Screening for genital chlamydia infection	Mixed populations	Health care services		10	6	Y	N	Y	Y
Majorin 2019 [393]	Interventions to improve disposal of child faeces for preventing diarrhoea and soil-transmitted helminth infection	Mixed populations	Living and working conditions		63	63	N	N	Y	Y
Mason-Jones 2016 [394]	School-based interventions for preventing HIV, sexually transmitted infections, and pregnancy in adolescents	Adolescents	Education	Individual lifestyle factors	8	8	N	N	Y	Y
Mayo-Wilson 2014 [395]	Zinc supplementation for preventing mortality, morbidity, and growth failure in children aged 6 months to 12 years of age	Infants/Children	Individual lifestyle factors		96	90	N	N	Y	Y
Mbuagbaw 2015 [396]	Health system and community level interventions for improving antenatal care coverage and health outcomes	Adults	Health care services		34	32	N	N	Y	Y
Moberley 2013 [397]	Vaccines for preventing pneumococcal infection in adults	Adults	Health care services		25	25	N	N	Y	Y
Moran 2016 [398]	Systematic screening for the detection of atrial fibrillation	Adults	Health care services		1	1	Y	N	Y	Y
Naude 2022 [399]	Low-carbohydrate versus balanced-carbohydrate diets for reducing weight and cardiovascular risk	Adults	Individual lifestyle factors		61	58	Y	N	Y	Y
Neil-Sztramko 2021 [400]	School-based physical activity programs for promoting physical activity and fitness in children and adolescents aged 6 to 18	Children/Adolescent s	Education	Individual lifestyle factors	89	77	N	N	Y	Y
O'Hara 2019 [54]	Video feedback for parental sensitivity and attachment security in children under five years	Vulnerable population - families with attachment issues	Other		22	20	Y	Y	Y	Y
Ota 2015 [401]	Antenatal dietary education and supplementation to increase energy and protein intake	Pregnant women/Planning	Education	Individual lifestyle factors	17	17	Y	N	Y	Y

Study	Title	Population type	Determinants of health		N studies included		Mention inequity/ inequality/social patterning	Focus on vulnerable group	Sub-group analysis	
			Primary	Secondary	Total	Quant			Plan	Complete
		pregnancy/New mothers								
Palacios 2019 [402]	Vitamin D supplementation for women during pregnancy	Pregnant women/Planning pregnancy/New mothers	Health care services	Individual lifestyle factors	30	30	Y	N	Y	Y
Pena-Rosas 2019 [47]	Fortification of rice with vitamins and minerals for addressing micronutrient malnutrition	Mixed populations	Agriculture and food production		17	12	Y	N	Y	Y
Qureshi 2021 [403]	Strategies for screening for familial hypercholesterolaemia in primary care and other community settings	Mixed populations	Health care services		0	0	N	N	Y	Y
Rees 2013 [404]	Dietary advice for reducing cardiovascular risk	Adults	Individual lifestyle factors	Education	7	5	N	N	Y	Y
Ried 2017 [405]	Effect of cocoa on blood pressure	Adults	Individual lifestyle factors		35	35	N	N	Y	Y
Ruotsalainen 2015 [406]	Preventing occupational stress in healthcare workers	Healthcare professionals	Work environment	Health care services	58	49	N	N	Y	Y
Santos 2019 [407]	Iodine fortification of foods and condiments, other than salt, for preventing iodine deficiency disorders	Mixed populations	Agriculture and food production		11	11	Y	N	Y	Y
Shah 2016 [49]	Fortification of staple foods with zinc for improving zinc status and other health outcomes in the general population	Mixed populations	Agriculture and food production		8	8	Y	N	Y	Y
Sherrington 2019 [408]	Exercise for preventing falls in older people living in the community	Adults	Individual lifestyle factors		108	102	N	N	Y	Y
Smith 2015 [409]	Mobile phone-based interventions for improving contraception use	Adults	Individual lifestyle factors		23	20	Y	N	Y	Y
Taylor 2017 [410]	Internet-based interventions for smoking cessation	Mixed populations	Individual lifestyle factors		67	34	Y	N	Y	Y
Vinceti 2018 [411]	Selenium for preventing cancer	Adults	Individual lifestyle factors	Health care services	83	83	Y	N	Y	Y

Study	Title	Population type	Determinants of health		N studies included		Mention inequity/ inequality/social patterning	Focus on vulnerable group	Sub-group analysis	
			Primary	Secondary	Total	Quant			Plan	Complete
von Philipsborn 2019 [50]	Environmental interventions to reduce the consumption of sugar-sweetened beverages and their effects on health	Mixed populations	Living and working conditions	Individual lifestyle factors	118	3	Y	N	Y	Y
Willcox 2020 [412]	Death audits and reviews for reducing maternal, perinatal and child mortality	Pregnant women/Planning pregnancy/New mothers	Health care services		2	0	Y	N	Y	Y
Wolfenden 2022 [413]	Strategies for enhancing the implementation of school-based policies or practices targeting diet, physical activity, obesity, tobacco or alcohol use	School stakeholders	Education	Individual lifestyle factors	38	14	N	N	Y	Y
