# Supplementary material for: Systematic Evaluation of How Indicators of Inequity and Disadvantage Are Measured and Reported in Population Health Evidence Syntheses
Source: Int J Environ Res Public Health. 2025 May 29;22(6):851. doi: 10.3390/ijerph22060851 (PMC12192879; doi:10.3390/ijerph22060851)
Supplement: Supplementary file 1 [file ijerph-22-00851-s001.zip › Suppl file S6 - Excluded reviews.pdf]

Supplementary file S6. Excluded reviews (n=33)

Review	Title	Exclusion	
		Category	Detail
Kaner 2017 [414]	Personalised digital interventions for reducing hazardous and harmful alcohol consumption in community-dwelling populations	Specific population	Participants defined by hazardous alcohol consumption
Dennis 2013 [415]	Psychosocial and psychological interventions for preventing postpartum depression	1-to-1 delivery	Limited to individual orientated interventions
Liddle 2015 [416]	Interventions for preventing and treating low-back and pelvic pain during pregnancy	Clinical population	Participants were pregnant women deemed at risk of/already with condition
Moe-Byrne 2016 [417]	Glutamine supplementation to prevent morbidity and mortality in preterm infants	Clinical population	Preterm infants were treated as a population with an existing condition (preterm)
Hemmingsen 2017 [418]	Diet, physical activity or both for prevention or delay of type 2 diabetes mellitus and its associated complications in people at increased risk of developing type 2 diabetes mellitus	Clinical population	Participants were diagnosed with intermediate hyperglycaemia or 'prediabetes'.
Sosa 2015 [419]	Bed rest in singleton pregnancies for preventing preterm birth	Clinical population	Preterm infants were treated as a population with an existing condition (preterm)
Kaner 2018 [420]	Effectiveness of brief alcohol interventions in primary care populations	Clinical population	Participants defined by presentation in healthcare setting
Brocklehurst 2013 [421]	Screening programmes for the early detection and prevention of oral cancer	LMIC	All studies conducted in LMIC (1 RCT in India)
Lamont 2018 [422]	Routine scale and polish for periodontal health in adults	Clinical intervention	Dental procedure taking place in healthcare setting
Smith 2017 [423]	Shared care across the interface between primary and specialty care in management of long term conditions	Clinical population	Participants were defined by having specified chronic disease(s) and being enrolled shared care service (primary and specialty care)
Amorim Adegboye 2013 [424]	Diet or exercise, or both, for weight reduction in women after childbirth	Specific population	Participants were women who had given birth to a singleton healthy term infant, and were overweight or obese, or had gained excessive weight during pregnancy, or both
Oliveira 2016 [425]	Vitamin A supplementation for postpartum women	LMIC	All studies conducted in LMIC (India, Bangladesh, Indonesia, Tanzania, Gambia, Zimbabwe, Kenya, Ghana, Peru, Brazil)
Lassi 2016 [426]	Zinc supplementation for the prevention of pneumonia in children aged 2 months to 59 months	LMIC	All studies conducted in LMIC (Bangladesh, Indi, Peru, South Africa)
Karmali 2017 [427]	Risk scoring for the primary prevention of cardiovascular disease	Clinical intervention	Intervention took place in primary care setting (systematic provision of multivariable CVD risk score by clinician, healthcare professional or system versus usual care)

Review	Title	Exclusion	
		Category	Detail
van Vilsteren 2015 [428]	Workplace interventions to prevent work disability in workers on sick leave	Clinical population	Participants defined by already having a particular condition which lead to them being on sick leave (back pain, MSK, mental health problems, cancer)
Imdad 2016 [429]	Vitamin A supplementation for the prevention of morbidity and mortality in infants one to six months of age	LMIC	All studies conducted in LMIC (Bangladesh, Nepal, Indonesia, Turkey, Ghana, Kenya, India, Peru)
Tzortziou Brown 2016 [430]	Professional interventions for general practitioners on the management of musculoskeletal conditions	Clinical population	Intervention related to primary care-based management of patients with MSK
McCauley 2015 [431]	Vitamin A supplementation during pregnancy for maternal and newborn outcomes	LMIC	All studies conducted in LMIC (Malawi, South Africa, Ghana, Tanzania, Indonesia, Bangladesh, Nepal, China, India)
Boyle 2014 [432]	Use of electronic health records to support smoking cessation	Clinical population	Intervention for patients presenting in healthcare setting
Schwenger 2015 [433]	Probiotics for preventing urinary tract infections in adults and children	Clinical population	Participants were those identified as susceptible to UTI
Lavender 2013 [434]	Telephone support for women during pregnancy and the first six weeks postpartum	1-to-1 delivery	Telephone support limited to individual-level
De-Regil 2017 [435]	Point-of-use fortification of foods with micronutrient powders containing iron in children of preschool and school-age	LMIC	All studies conducted in LMIC (India, Indonesia, Lao People's Democratic Republic, Kyrgyz Republic, China, South Africa, Kenya, Honduras, Colombia
Thomas 2017 [436]	System change interventions for smoking cessation	Clinical population	Participants were smokers receiving care in a healthcare setting (or the staff supporting those patients)
Suchdev 2015 [437]	Multiple micronutrient powders for home (point-of-use) fortification of foods in pregnant women	LMIC	All studies conducted in LMIC (Bangladesh, Mexico)
Lindson 2021 [438]	Strategies to improve smoking cessation rates in primary care	Clinical population	Participants were primary healthcare patients
Fair 2019 [439]	Interventions for supporting the initiation and continuation of breastfeeding among women who are overweight or obese	Specific population	Women who were breastfeeding and were overweight/obese
Garn 2022 [440]	Interventions to improve water, sanitation, and hygiene for preventing soil-transmitted helminth infection	LMIC	All studies conducted in LMIC
Grev 2018 [441]	Maternal probiotic supplementation for prevention of morbidity and mortality in preterm infants	Clinical population	Preterm infants were treated as a population with an existing condition (perterm)
Das 2018 [442]	Lipid-based nutrient supplements for maternal, birth, and infant developmental outcomes	LMIC	All studies conducted in LMIC (Ghana, Malawi, Burkina Faso, Bangladesh)
Das 2019 [443]	Preventive lipid-based nutrient supplements given with complementary foods to infants and young children 6 to 23 months of age for health, nutrition, and developmental outcomes	LMIC	All studies conducted in LMIC (Malawi, Bangladesh, Ghana, Burkina Faso, Chad, Congo, Guatemala, Haiti, Honduras, Kenya, Peru)

Review	Title	Exclusion	
		**Category**	**Detail**
Baxter 2022 [444]	Fortification of salt with iron and iodine versus fortification of salt with iodine alone for improving iron and iodine status	LMIC	All studies conducted in LMIC (India, Morocco, Cote d'Ivoire, Ghana, Sri Lanka)
Vonasek 2021 [445]	Screening tests for active pulmonary tuberculosis in children	Diagnostic accuracy	Objectives related to sensitivity and specificity of the screening methods
Viswanathan 2020 [446]	Universal screening for SARS-CoV2 infection: a rapid review	Diagnostic accuracy	Objectives related to effectiveness and accuracy of the screening methods
