# Supplementary material for: Systematic Evaluation of How Indicators of Inequity and Disadvantage Are Measured and Reported in Population Health Evidence Syntheses
Source: Int J Environ Res Public Health. 2025 May 29;22(6):851. doi: 10.3390/ijerph22060851 (PMC12192879; doi:10.3390/ijerph22060851)
Supplement: Supplementary file 1 [file ijerph-22-00851-s001.zip › Suppl file S7 - Distribution by wider determinant categories.pdf]

Supplementary file S7. Number of reviews mapped to determinants of health categories (primary/secondary)

Determinants of health category	Wider determinant	Primary category		Secondary category		Total
		n	%	n	%	n
Agriculture and food production	Y	14	4.0	0	0.0	14
Education	Y	30	8.6	3	0.9	33
General socio-economic	Y	2	0.6	0	0.0	2
Health care services	N	97	27.7	19	5.4	116
Housing	Y	2	0.6	0	0.0	2
Individual lifestyle factors	N	155	44.3	29	8.3	184
Living and working conditions	Y	8	2.3	8	2.3	16
Other	N	24	6.9	4	1.1	28
Social and community networks	N	4	1.1	7	2.0	11
Unemployment	Y	1	0.3	0	0.0	1
Water and sanitation	Y	4	1.1	0	0.0	4
Work environment	Y	22	6.3	1	0.3	23
**Total**		**363**		**71**		**434**

% derived as proportion of the 363 reviews
