# Supplementary material for: Systematic Evaluation of How Indicators of Inequity and Disadvantage Are Measured and Reported in Population Health Evidence Syntheses
Source: Int J Environ Res Public Health. 2025 May 29;22(6):851. doi: 10.3390/ijerph22060851 (PMC12192879; doi:10.3390/ijerph22060851)
Supplement: Supplementary file 1 [file ijerph-22-00851-s001.zip › Suppl file S8 - Breakdown of equity indicators (Place, SES).pdf]

Supplementary file S8. Breakdown of Place and SES categories planned for subgroup analysis

*Number of reviews that planned subgroup analysis by different **Age** indicators*

Type of age category	n	%
Specific years	46	50.5
School year/stage	5	5.5
Life stage	17	18.7
Not specified	23	25.3
**Total**	**91**	

*Number of reviews that planned subgroup analysis by different **Gender/Sex** indicators*

Type of category		n	%
Sex at birth		22	31.9
Gendered labels		18	26.1
	Male, female	22	
	Boys, girls	4	
	Men, women	7	
	Men, women, mixed populations	5	
	Mothers, fathers, mixed populations	1	
	Women, men, mixed	1	
Not specified		29	42.0
**Total**		**69**	

*Number of reviews that planned subgroup analysis by different **Place** indicators*

**Place indicators**<br>**Indicator**	**Categories**	**Number of reviews**	**% of reviews**
Community water supply	Available water supply, Scare water supply	1	2.1
Community dwelling	Residential vs. community	1	2.1
Country	Not specified	1	2.1
Country income level	LMIC vs. HIC; Low vs. middle vs. high	37	78.7
Latitude	Between tropics of Cancer and Capricorn, North of the Tropic of Cancer or South of the Tropic of Capricorn	1	2.1
Malaria endemicity	Endemic vs. malaria-free area	1	2.1
Place of residence	Not specified	1	2.1
Urban/rural	Urban vs. rural	7	14.9
Geographical region	Not specified; Africa, Asia, Caribbean, Central America, Europe, North America, Oceania, and South America	2	4.3
**Total use of indicator**		**52**	

% derived as proportion of 47 reviews (5 reviews used two Place indicators, giving a total of 52); LMIC, low-middle-income country; HIC, high-income country

*Number of reviews that planned subgroup analysis by different **SES** indicators*

**Type of socio-economic indicator**	**Categories (Number of reviews in parentheses)**	**Number of reviews**	**% of reviews**
Income			
- Area-level	High vs. low income (n=1)	2	5.4
- Personal/household level	High vs. low income (n=1); High vs. middle vs. low income (n=1)	5	13.5
Disadvantage, social disadvantage, or deprivation	Disadvantaged vs. not disadvantaged (n=3); more vs. less socially deprived (n=1); low vs. high deprivation- (n=1); social disadvantage vs. not (n=1); proxies (poor/less poor, under-/well-nourished) (n=1); not specified (n=2)	9	24.3
Socio-economic status	High vs. low (2); Low vs. any (1); Low vs. not low (n=1); Low vs. mixed vs. any (n=1); Not specified (n=14)	19	51.4
Other	Low-income or minority ethnic vs. general population (n=1); Unspecified community socio-economic contextual factors (n=1)	2	5.4
**Total**		**37**	

% derived as proportion of 35 reviews
