# Supplementary figures and images for: Systematic Evaluation of How Indicators of Inequity and Disadvantage Are Measured and Reported in Population Health Evidence Syntheses
Source: Int J Environ Res Public Health. 2025 May 29;22(6):851. doi: 10.3390/ijerph22060851 (PMC12192879; doi:10.3390/ijerph22060851)

#### Supplementary file S4. Summary of descriptive analysis of included reviews

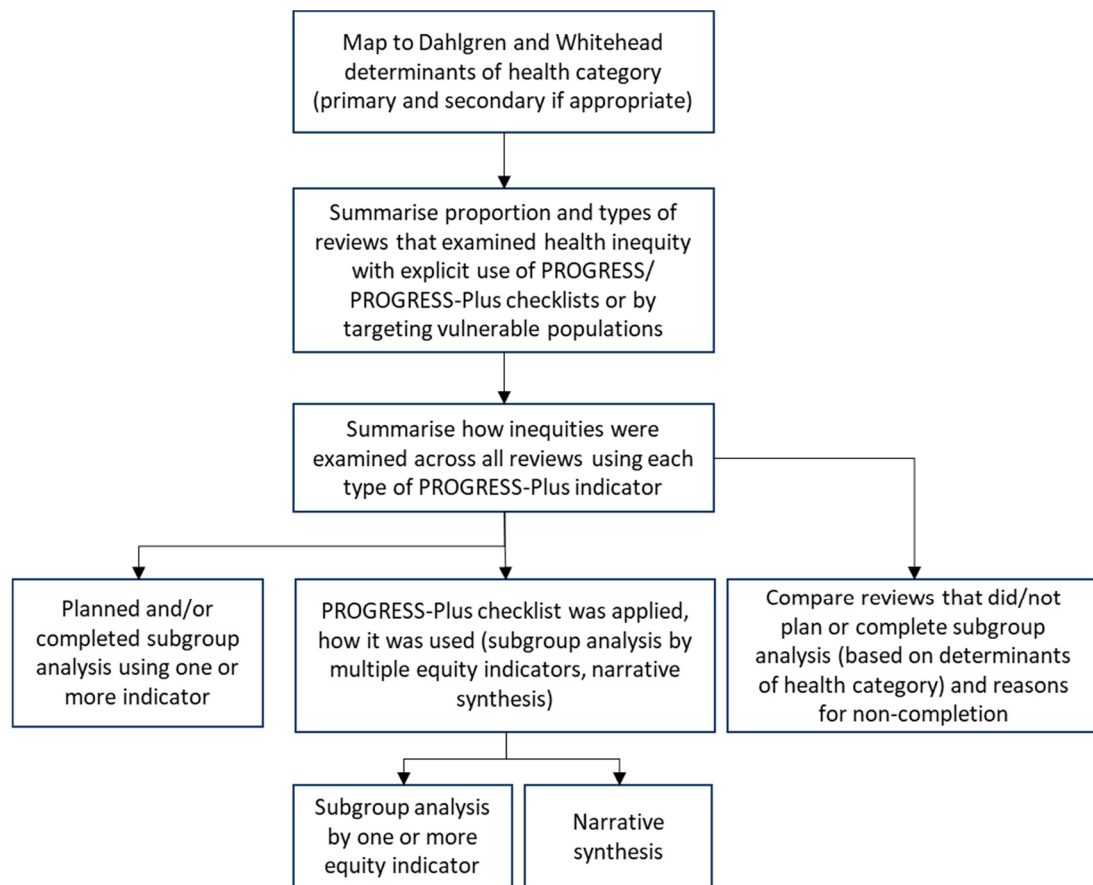

Supplement: Supplementary file 1 [file ijerph-22-00851-s001.zip › Suppl file S4 - Summary of analysis.pdf]
